# Integrative genetics-metabolomics analysis of infant bronchiolitis-childhood asthma link: A multicenter prospective study

**DOI:** 10.3389/fimmu.2022.1111723

**Published:** 2023-02-02

**Authors:** Tadao Ooka, Zhaozhong Zhu, Liming Liang, Juan C. Celedon, Brennan Harmon, Andrea Hahn, Eugene P. Rhee, Robert J. Freishtat, Carlos A. Camargo, Kohei Hasegawa

**Affiliations:** ^1^ Department of Emergency Medicine, Massachusetts General Hospital, Harvard Medical School, Boston, MA, United States; ^2^ Department of Health Science, University of Yamanashi, Chuo, Yamanashi, Japan; ^3^ Program in Genetic Epidemiology and Statistical Genetics, Harvard T. H. Chan School of Public Health, Boston, MA, United States; ^4^ Department of Biostatistics, Harvard T. H. Chan School of Public Health, Boston, MA, United States; ^5^ Division of Pediatric Pulmonary Medicine, UPMC Children’s Hospital of Pittsburgh, University of Pittsburgh, Pittsburgh, PA, United States; ^6^ Center for Genetic Medicine Research, Children’s National Hospital, Washington, DC, United States; ^7^ Department of Pediatrics, George Washington University School of Medicine and Health Sciences, Washington, DC, United States; ^8^ Division of Infectious Diseases, Children’s National Hospital, Washington, DC, United States; ^9^ Division of Nephrology, Department of Medicine, Massachusetts General Hospital, Harvard Medical School, Boston, MA, United States; ^10^ Division of Emergency Medicine, Children’s National Hospital, Washington, DC, United States

**Keywords:** asthma, bronchiolitis, childhood asthma, genetics, integrated-omics, metabolomics, phosphatidylglycerol, sphingolipids

## Abstract

**Background:**

Infants with bronchiolitis are at high risk for developing childhood asthma. While genome-wide association studies suggest common genetic susceptibilities between these conditions, the mechanisms underlying the link remain unclear.

**Objective:**

Through integrated genetics-metabolomics analysis in this high-risk population, we sought to identify genetically driven metabolites associated with asthma development and genetic loci associated with both these metabolites and asthma susceptibility.

**Methods:**

In a multicenter prospective cohort study of infants hospitalized for bronchiolitis, we profiled the nasopharyngeal metabolome and genotyped the whole genome at hospitalization. We identified asthma-related metabolites from 283 measured compounds and conducted metabolite quantitative trait loci (mtQTL) analyses. We further examined the mtQTL associations by testing shared genetic loci for metabolites and asthma using colocalization analysis and the concordance between the loci and known asthma-susceptibility genes.

**Results:**

In 744 infants hospitalized with bronchiolitis, 28 metabolites (e.g., docosapentaenoate [DPA], 1,2-dioleoyl-sn-glycero-3-phosphoglycerol, sphingomyelin) were associated with asthma risk. A total of 349 loci were associated with these metabolites—161 for non-Hispanic white, 120 for non-Hispanic black, and 68 for Hispanics. Of these, there was evidence for 30 shared loci between 16 metabolites and asthma risk (colocalization posterior probability ≥0.5). The significant SNPs within loci were aligned with known asthma-susceptibility genes (e.g., *ADORA1*, *MUC16*).

**Conclusion:**

The integrated genetics-metabolomics analysis identified genetically driven metabolites during infancy that are associated with asthma development and genetic loci associated with both these metabolites and asthma susceptibility. Identifying these metabolites and genetic loci should advance research into the functional mechanisms of the infant bronchiolitis-childhood asthma link.

## Introduction

Bronchiolitis is the leading cause of infant hospitalization in the U.S., accounting for 110,000 hospitalizations each year (1). Its chronic morbidity is also substantial. Among infants hospitalized with bronchiolitis (i.e., severe bronchiolitis), ~30% subsequently develop childhood asthma (2–6). Yet, the mechanisms underlying the bronchiolitis-asthma link remain unclear. Our limited understanding has hindered the development of asthma prevention strategies.

Asthma is a complex syndrome that is influenced by both genetic and environmental factors (e.g., early-life virus infection) (7). Metabolomics systematically profiles small molecules in a biological system, which represent the downstream functional products of these genetic and environmental interactions. Studies have suggested metabolites involved in asthma pathobiology—e.g., sphingolipids (e.g., sphingomyelins) (8, 9), phospholipids (e.g., phosphatidylglycerol [PG]) (10), and fatty acids (e.g., docosapentaenoate [DPA]) (11). In addition to metabolomics, genome-wide association studies (GWASs) have identified genetic loci for childhood asthma susceptibility (12–17). For example, *ORMDL3* located at chromosome 17q21—a major regulator of sphingolipid metabolism—plays an important role in asthma development (12, 15). Metabolomics and genetics studies have *independently* attempted to elucidate the mechanisms underlying asthma pathobiology. However, no study has yet examined the integrated relationship of genetics, airway metabolome, and asthma development in children—let alone in infants at high risk for asthma development.

To address this knowledge gap, we applied an integrative genetics-metabolomics approach to data from a multicenter prospective cohort study of infants with severe bronchiolitis. We sought to identify the genetically driven metabolites and the genetic loci regulating those metabolites associated with the development of childhood asthma. 

## Materials and methods

### Study design, setting, and participants

We analyzed data from the 35th Multicenter Airway Research Collaboration (MARC-35) study—a multicenter prospective cohort study (18). Details of the study design, setting, participants, data collection, testing, and statistical analysis may be found in the **Supplementary Methods**. Briefly, investigators enrolled 1,016 infants (age <1 year) hospitalized with attending physician-diagnosis of bronchiolitis at 17 sites across 14 U.S. states (**Table S1**) in 2011–2014. The diagnosis of bronchiolitis was made according to the American Academy of Pediatrics bronchiolitis guidelines (19), defined as an acute respiratory illness with a combination of rhinitis, cough, tachypnea, wheezing, crackles, or chest retractions. We excluded infants with a known heart-lung disease, immunodeficiency, immunosuppression, or gestational age of <32 weeks. All patients were treated at the discretion of the treating physicians.

Of 1,016 infants enrolled in the MARC-35 cohort, the current analysis investigated 744 infants who underwent both genotyping and nasopharyngeal metabolome profiling (**Table S2**). The institutional review board at each participating hospital approved the study with written informed consent obtained from the parent or guardian.

### Data collection

Clinical data (patients’ demographic characteristics, medical, environmental, and family history, and details of the acute illness) were collected *via* structured interviews and chart reviews using a standardized protocol (8, 9). After the index hospitalization for bronchiolitis, trained interviewers began interviewing parents/legal guardians by telephone at 6-month intervals in addition to medical record review by physicians. All data were reviewed at the Emergency Medicine Network Coordinating Center at Massachusetts General Hospital (Boston, Massachusetts, USA) (18). By using a standardized protocol (8), investigators collected peripheral blood specimens (for genotyping) and nasopharyngeal specimens (for metabolome profiling) within 24 hours of hospitalization. We described the details of the data collection and measurement methods in the **Supplementary Methods**.

### Genotyping

We used the Illumina Multi-Ethnic Genotyping Array (San Diego, California) for genotyping. For genotype imputation, we used the TOPMed reference panel on the TOPMed Imputation Server (20) and removed variants with an imputation score of <0.6 from the imputed dataset. We also removed rare variants with a minor allele frequency of <0.01 from the dataset. Subsequently, we included a total of 10,852,874 autosomal variants for the downstream association study. We described the details of genotype imputation and quality control in the **Supplementary Methods**.

### Nasopharyngeal airway metabolome profiling

We profiled the nasopharyngeal metabolome using liquid chromatography with tandem mass spectrometry (LC-MS/MS) at Metabolon (Morrisville, North Carolina). The laboratory processed the blinded specimens in random order. Instrument variability was 4%, as determined by calculating the median relative standard deviation for the internal standards. The metabolome profiling identified 283 known metabolites from 76 sub-pathways within 7 super-pathways. We described the details of metabolome profiling in a previous study (21) and **Supplementary Methods**.

### Clinical outcome measure

The clinical outcome of interest is the development of asthma by age 6 years. Asthma was defined using a commonly used epidemiologic definition: physician-diagnosis of asthma, with either asthma medication use (e.g., albuterol, inhaled corticosteroids) or asthma-related symptoms (e.g., wheezing, nocturnal cough) in the preceding year (22).

### Statistical analysis

**Figure 1:**
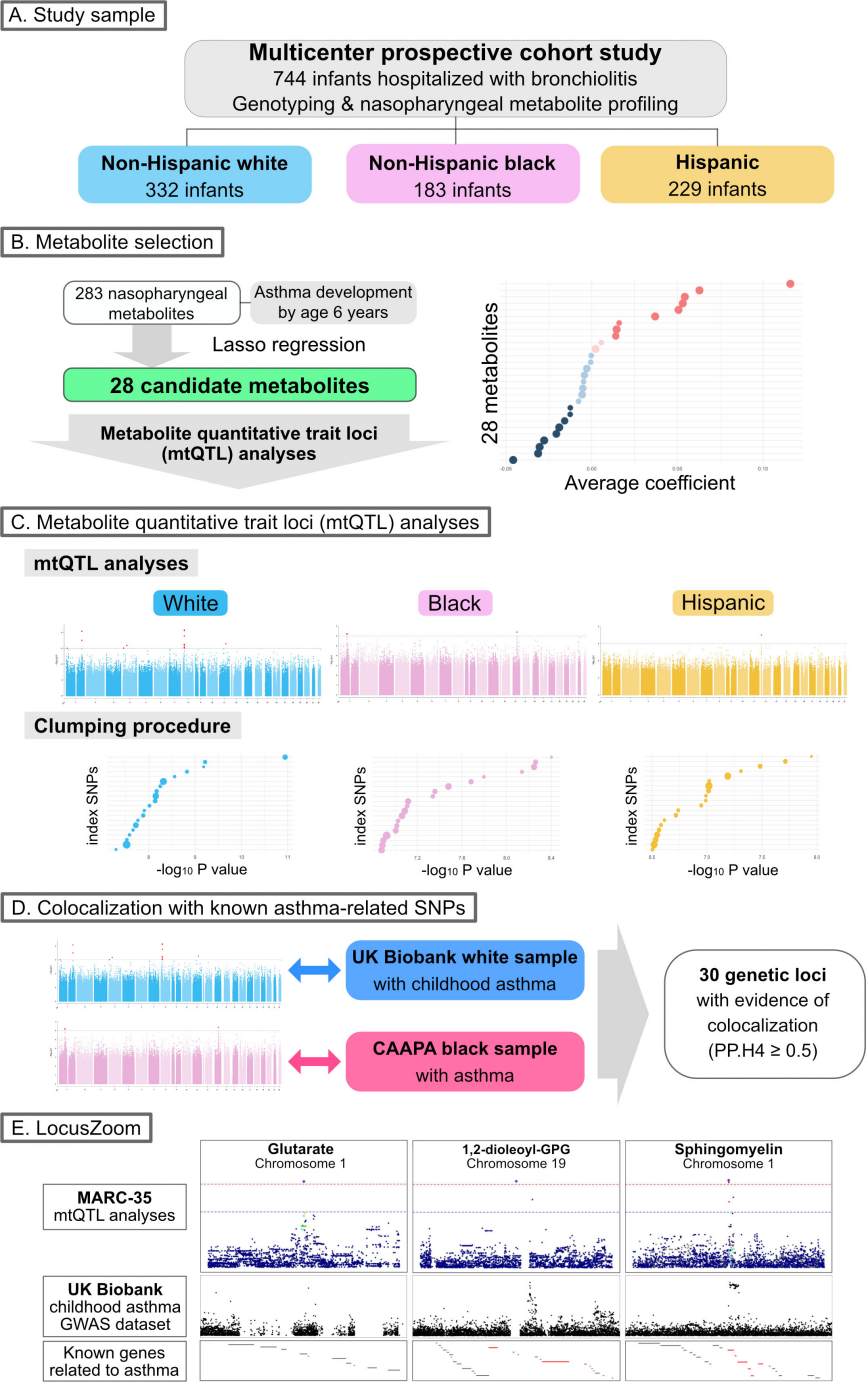
Analytic workflow of the study **(A)**. Of 744 infants (age <1 year) hospitalized with bronchiolitis, the current analysis investigated 744 infants—332 (45%) were non-Hispanic white, 183 (25%) were non-Hispanic black, and 229 (31%) were Hispanics—who underwent both genotyping and nasopharyngeal metabolome profiling. **(B)**. The association of 283 nasopharyngeal metabolites of the infants with asthma development by age 6 years was examined—by constructing Lasso regression models—to identify candidate metabolites for the downstream metabolite quantitative trait loci (mtQTL) analysis. **(C)**. The mtQTL analysis was performed to examine the association between 28 candidate metabolites and genotypes in each of the three major racial/ethnic subgroups of infants with bronchiolitis. The clumping procedure was also conducted to determine mtQTLs that are independent of each other. **(D)**. Bayesian colocalization analysis using summary statistics of the mtQTLs of MARC-35, UK Biobank (white sample with childhood asthma), and CAAPA (African-admixed sample with asthma) was conducted to identify shared genetic loci between the mtQTLs and the independent GWAS summary statistics for asthma. **(E)**. The association peaks of the 30 loci with colocalization evidence were compared between the GWAS summary statistics of the mtQTL analysis of MARC-35 and the UK Biobank. The association peaks of the three loci were aligned with those in the same genetic regions of UK Biobank summary statistics for childhood asthma, and there were concordances between the peaks of the two loci and known genes related to asthma. Abbreviations: CAAPA, Consortium on Asthma among African-ancestry Populations in the Americas; GPG, glycero-3-phosphoglycerol; GWAS, genome-wide association studies; MARC, multicenter airway research collaboration; PP.H4, posterior probability of hypothesis 4; SNP, single nucleotide polymorphism.

The objectives of the present study are (i) to identify genetically driven metabolites that are associated with the risk of developing asthma and (ii) to examine the genetic loci that are associated with both these metabolites and asthma susceptibility. The analytic workflow is summarized in **Figure 1**. The details of the statistical analysis may be found in the **Supplementary Methods**.

Briefly, we first constructed logistic regression models with Lasso regularization that examines the association of the nasopharyngeal metabolome with asthma development to identify candidate metabolites for the subsequent metabolite quantitative trait loci (mtQTL) analysis. Second, we performed the mtQTL analysis with an additive linear regression genetic association analysis adjusting for age, sex, and the first two ancestry principal components to examine the association between the genotypes and candidate metabolites in each of the three major racial/ethnic samples (non-Hispanic white, non-Hispanic black, and Hispanics) in the MARC-35 cohort. The significance threshold for these analyses was set to P<1×10^-6^. Third, we conducted clumping procedures to determine mtQTLs that are independent of each other. Fourth, we conducted pathway analyses to examine the biological significance of the genetic loci identified in the mtQTL analysis by using all genes within the clumped region of those loci. Fifth, we conducted Bayesian colocalization analyses to examine whether genetic loci for candidate metabolites are shared with those for asthma by using the GWAS summary statistics of the mtQTL analysis from MARC-35 and those of two independent datasets—the UK Biobank (white sample with childhood asthma (23–25)) and the Consortium on Asthma among African-ancestry Populations in the Americas (CAAPA; African-admixed sample with asthma (26)). We selected variants within 500 kb of the index SNP—the SNP with the smallest (i.e., most significant) P-value in each clumped region—at each of the shared loci in the non-Hispanic white and non-Hispanic black samples, and estimated the posterior probability that the two traits (i.e., each metabolite and asthma risk) share one common causal variant (a posterior probability of hypothesis 4 [PP.H4]). We considered loci with a posterior probability of ≥0.5 to colocalize. Lastly, we visualized the index SNPs colocalized with childhood asthma-risk loci from the UK Biobank GWAS summary statistics by using LocusZoom (27).

## Results

Of 1,016 infants enrolled in the MARC-35 cohort, the current study focused on 744 infants with severe bronchiolitis who underwent both genotyping and nasopharyngeal metabolome profiling. The analytic (n=744) and non-analytic (n=272) cohorts did not differ in the patient characteristics (P≥0.05; **Table S2**), except for the proportion of racial/ethnicity and rhinovirus infection. Of the infants in the analytic cohort, the median age was 3 (interquartile range [IQR], 2-6) months and 40% were female; 45% were non-Hispanic white, 25% were non-Hispanic black, and 31% were Hispanics (**Table 1**). Overall, 26% subsequently developed asthma by age 6 years (**Table S3**).

**Table 1:** Baseline characteristics and clinical course of 744 infants hospitalized with bronchiolitis, according to race/ethnicity.

	Overall	Non-Hispanic white	Non-Hispanic black	Hispanic	P value
Characteristics	(n=744; 100%)	(n=332; 45%)	(n=183; 25%)	(n=229; 31%)	
Demographics					
Age (month), median (IQR)	3 (2-6)	3 (2-6)	3 (2-6)	4 (2-6)	0.67
Female sex	300 (40)	134 (40)	80 (44)	86 (38)	0.45
Prematurity (32.0-36.9 weeks)	136 (18)	55 (17)	43 (24)	38 (17)	0.11
C-section delivery	250 (34)	118 (36)	60 (34)	72 (32)	0.57
Previous breathing problems (count)					0.76
0	587 (79)	268 (81)	142 (78)	177 (77)	
1	122 (16)	51 (15)	30 (16)	41 (18)	
2	35 (5)	13 (4)	11 (6)	11 (5)	
Previous ICU admission	12 (2)	4 (1)	2 (1)	6 (3)	0.35
History of eczema	111 (15)	45 (14)	39 (21)	27 (12)	0.02
Ever attended daycare	170 (23)	78 (24)	57 (31)	35 (15)	0.001
Parental history of asthma	246 (33)	107 (32)	73 (40)	66 (29)	0.04
Parental history of eczema	143 (19)	64 (19)	55 (30)	24 (11)	<0.001
Clinical presentation					
Weight (kg), median (IQR)	6 (5-8)	6 (5-8)	6 (5-8)	6 (5-8)	0.39
Respiratory rate (per minute), median (IQR)	48 (40-60)	48 (40-60)	52 (40-61)	48 (40-60)	0.08
Oxygen saturation					0.01
<90%	62 (9)	27 (8)	9 (5)	26 (12)	
90-93%	113 (16)	60 (19)	17 (9)	36 (16)	
≥94%	553 (76)	233 (73)	156 (86)	164 (73)	
Blood eosinophilia (≥4%)	70 (11)	32 (11)	17 (11)	21 (11)	0.99
IgE sensitization	155 (21)	52 (16)	54 (30)	49 (21)	0.001
Clinical course					
Positive pressure ventilation use*	39 (5)	20 (6)	5 (3)	14 (6)	0.21
Intensive treatment use†	114 (15)	50 (15)	23 (13)	41 (18)	0.32
Length-of-day (day), median (IQR)	2 (1-3)	2 (1-3)	2 (1-3)	2 (1-3)	0.03
Respiratory virus					
RSV only	421 (57)	210 (63)	93 (51)	118 (52)	0.004
RV only	52 (7)	17 (5)	17 (9)	18 (8)	0.17
RSV/RV coinfection	81 (11)	34 (10)	24 (13)	23 (10)	0.54
Other pathogen‡	179 (24)	72 (22)	45 (25)	62 (27)	0.33

ICU, intensive care unit; IgE, immunoglobulin E; IQR, interquartile range; RSV, respiratory syncytial virus; RV, rhinovirus.

Data are no. (%) of infants unless otherwise indicated. Percentages may not equal 100 because of rounding and missingness.

*Infants with bronchiolitis who underwent continuous positive airway ventilation and/or mechanical ventilation.

†Infants with bronchiolitis who were admitted to ICU and/or who underwent positive pressure ventilation.

‡Adenovirus, bocavirus, Bordetella pertussis, enterovirus, human coronavirus NL63, OC43, 229E, or HKU1, human metapneumovirus, influenza A or B virus, Mycoplasma pneumoniae, and parainfluenza virus 1-3.

### mtQTL analysis reveals nasopharyngeal airway metabolites at infant bronchiolitis that are genetically driven

**Figure 2:**
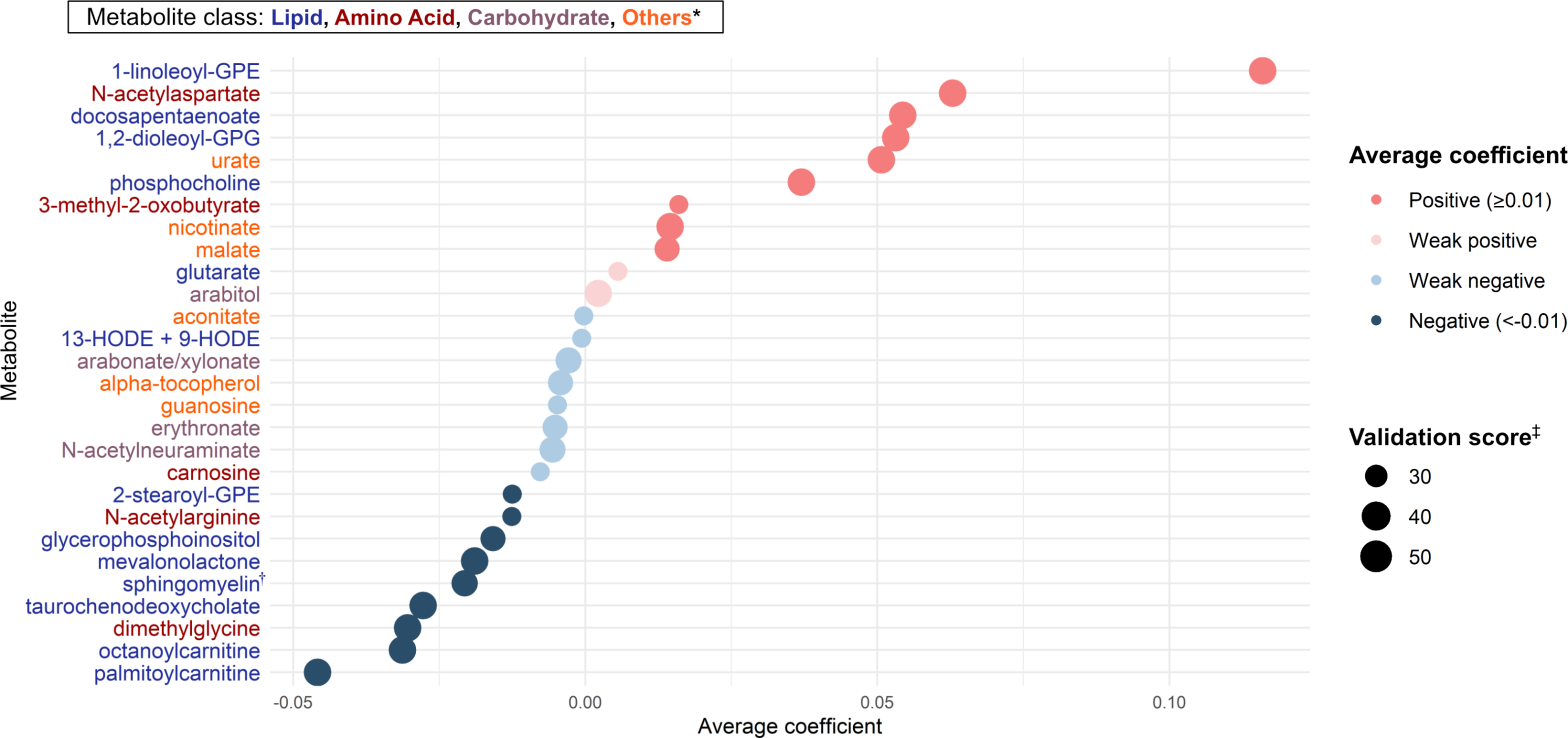
Metabolites selection for the mtQTL analyses By applying the Lasso regression models on nasopharyngeal metabolome (283 metabolites) data and the asthma outcome data of 744 infants hospitalized for bronchiolitis, we identified 28 candidate metabolites for the downstream mtQTL analyses. The average coefficient indicates the magnitude of the metabolite-outcome association averaged over times selected as a feature in 100 Lasso regression model executions. * Of 28 metabolites, 13 were lipids, 5 were amino acids, 4 were carbohydrates, and 6 were other classes (e.g., vitamins, nucleic acids) of metabolites. † Sphingomyelin (d17:1/16:0, d18:1/15:0, d16:1/17:0) ‡ The number of times selected as a feature of the Lasso regression model in 100 model executions. Abbreviations: GPE, glycerophosphorylethanolamine; GPG, glycero-3-phosphoglycerol; HODE, hydroxyoctadecadienoic acid.

Of 283 nasopharyngeal metabolites identified in infants with bronchiolitis, 28 candidate metabolites were associated with the risk of developing asthma based on the Lasso regression models (**Figure 2**). Of these 28 candidate metabolites, 13 were lipids (e.g., DPA, 1,2-dioleoyl-sn-glycero-3-phosphoglycerol [GPG], glutarate, sphingomyelin [d17:1/16:0, d18:1/15:0, d16:1/17:0]), 5 were amino acids (e.g., N-acetylarginine), 4 were carbohydrates (e.g., arabitol), and 6 were other classes of metabolites.

**Figure 3:**
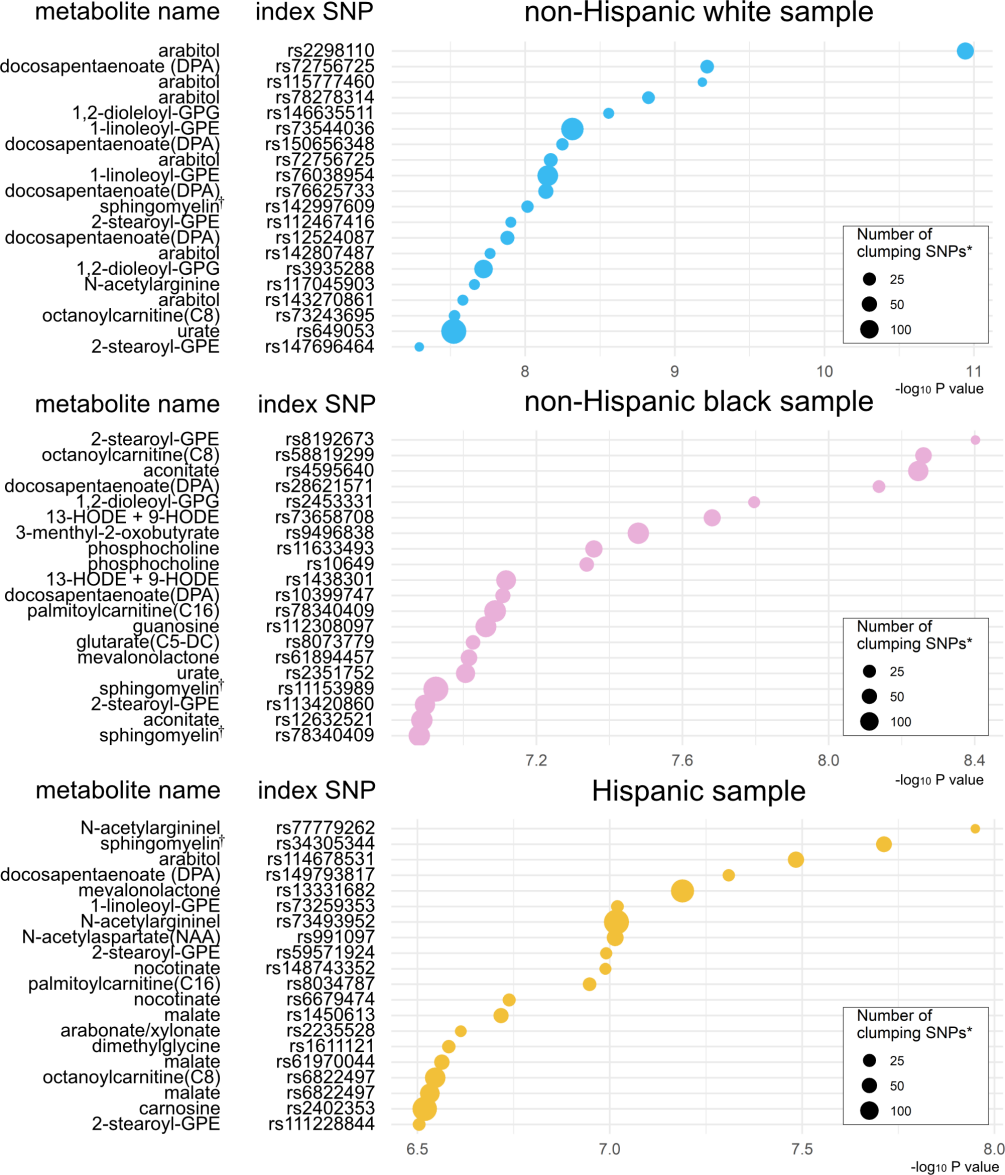
Significant index SNPs identified in metabolite quantitative trait loci (mtQTL) analyses The 20 index SNPs with the lowest P-values associated with the candidate metabolites were identified in the mtQTL analysis of each racial/ethnic sample after the clumping procedure. * Variants with a P value of <1 × 10^−3^, R^2^ of ≥0.2, and <500 kb away from the index SNP were assigned to the clump. † Sphingomyelin (d17:1/16:0, d18:1/15:0, d16:1/17:0) Abbreviations: GPE, glycerophosphorylethanolamine; GPG, glycero-3-phosphoglycerol; HODE, hydroxyoctadecadienoic acid.

Based on the mtQTL analysis for each of the 28 candidate metabolites, 900 SNPs were at a suggestive significance level (28) (P <1×10^-6^)—524 in non-Hispanic white, 259 in non-Hispanic black, and 117 in Hispanics (**Figure S1** and **Table S4**). The clumping procedure for these SNPs showed that 349 loci were independently associated with the candidate metabolites—161 loci associated with 26 metabolites in non-Hispanic white, 120 loci with 28 metabolites in non-Hispanic black, and 68 loci with 25 metabolites in Hispanics (**Figure 3**, **Tables S5**, **S6**). The pathway analysis showed the biological importance of these loci with significant pathways (FDR<0.05; **Figure S2**), which are relevant to both bronchiolitis and asthma development—e.g., interferon-α/β (29), tumor necrosis factor receptor-associated factor 6 (TRAF6) mediated interferon regulatory factor 7 (IRF7) activation pathways (30).

### Colocalization analysis demonstrates 30 genetic loci associated with both metabolites and asthma-susceptibility

To test for genetic loci that are in common between the candidate metabolites and asthma risk, colocalization analyses were performed for the 281 loci identified in the clumping procedure—161 in non-Hispanic white and 120 in non-Hispanic black—by comparing the GWAS summary statistics of the mtQTL analysis in MARC-35 to two independent datasets—the UK Biobank and CAAPA (**Table S5** and **Figure S3**). There was evidence for 30 shared genetic loci between 16 metabolites and asthma risk (PP.H4 ≥0.5; **Table 2**)—27 loci in UK Biobank (e.g., chromosome 6q26 with DPA, chromosome 1q21 with glutarate, chromosome 1q32 with sphingomyelin [d17:1/16:0, d18:1/15:0, d16:1/17:0]), 1 locus in CAAPA (chromosome 6q12 with N-acetylarginine), and 2 loci with both datasets (e.g., chromosome 14q31 with 1,2-dioleoyl-GPG).

**Table 2:** Summary of the 30 genetic loci associated with both the candidate metabolites and asthma risk.

Metabolite		Locus											
Metabolite class	Metabolite name	Index SNP^*^	N^†^	Chr	Index SNP^*^ position		Alt allele	P value			PP.H4^‡^		Known asthma genes within index SNP^*^ ± 500KB
								Non-Hispanic white	Non-Hispanic black		UKB white	CAAPA black	
Significant loci in the non-Hispanic white sample													
Lipid	1,2-dioleoyl-GPG (18:1/18:1)	rs116459436	1	1	7227920	T		5.0×10^-07^		0.009	0.513	0.267	
		rs1637750	16	7	2188176	G		9.3×10^-07^		0.740	0.656	0.259	*AMZ1*
		rs146635511	2	10	124772492	T		2.8×10^-09^		NA	0.651	0.235	*LHPP*
		rs142277549	3	14	88122695	T		2.1×10^-07^		0.109	0.691	0.695	
		rs113043905	1	19	8717141	G		6.0×10^-07^		0.453	0.580	0.251	*ADAMTS10,ACTL9,MUC16,OR1M1*
Lipid	docosapentaenoate (DPA; 22:5n3)	rs78388829	5	2	15120216	G		5.1×10^-07^		0.784	0.695	0.278	*FAM84A,DDX1*
		rs12524087	12	6	163619816	G		1.3×10^-08^		0.574	0.841	0.287	*QKI*
Lipid	glutarate (C5-DC: glutarylcarnitine)	rs2232187	3	1	147759651	A		6.5×10^-07^		0.656	0.768	0.277	
Lipid	mevalonolactone	rs4752744	11	11	1697036	G		2.7×10^-07^		0.869	0.967	0.237	*MUC5B*
Amino acid	N-acetylarginine	rs9363451	29	6	65822620	A		2.9×10^-07^		0.001	0.145	0.556	
		rs117045903	2	15	66627247	A		2.2×10^-08^		0.294	0.698	0.289	*SMAD3,SMAD6,LINC01169*
Carbohydrate	arabitol	rs2298110	34	1	19906558	G		1.1×10^-11^		0.077	0.936	0.398	
		rs113812800	1	2	3901708	C		2.4×10^-07^		0.053	0.738	0.383	*ALLC*
		rs143270861	2	4	182064534	A		2.6×10^-08^		0.707	0.698	0.258	*MGC45800*
		rs185153229	7	6	34748997	C		7.0×10^-08^		0.767	0.625	0.203	*TCP11,SCUBE3*
		rs115777460	1	13	52988125	A		6.6×10^-10^		0.817	0.728	0.381	
		rs77244206	7	15	31337658	G		7.6×10^-07^		0.610	0.957	0.377	
Carbohydrate	N-acetylneuraminate	rs10101380	119	8	5147537	C		9.1×10^-07^		0.760	0.586	0.187	*CSMD1*
Energy	aconitate	rs148027659	4	10	74609570	G		5.8×10^-07^		0.633	0.514	0.214	
Significant loci in the non-Hispanic black sample													
Lipid	2-stearoyl-GPE (18:0)	rs12494581	36	3	55257122	C		0.524		7.3×10^-07^	0.812	0.506	
Lipid	palmitoylcarnitine (C16)	rs79141561	4	2	105989161	G		NA		8.1×10^-07^	0.550	0.472	
Lipid	sphingomyelin (d17:1/16:0, d18:1/15:0, d16:1/17:0)	rs12752641	8	1	203053298	C		0.475		5.0×10^-07^	0.688	0.226	*PPFIA4,MYOG,ADORA1,MYBPH,CHI3L1,CHIT1*
		rs78340409	29	1	234553708	G		0.852		1.3×10^-07^	0.659	0.164	
Lipid	taurochenodeoxycholate	rs72816230	7	17	1219933	G		0.023		6.2×10^-07^	0.626	0.249	
Amino acid	dimethylglycine	rs8052562	1	16	3491830	T		0.436		7.2×10^-07^	0.686	0.268	*TRAP1*
Amino acid	N-acetylaspartate	rs11714340	64	3	27610676	C		0.211		5.0×10^-07^	0.581	0.220	
Carbohydrate	N-acetylneuraminate	rs76840346	84	13	53885705	C		0.063		3.5×10^-07^	0.511	0.272	
Cofactors and vitamins	alpha-tocopherol	rs12942941	13	17	80129214	G		0.476		9.0×10^-07^	0.652	0.239	
Energy	aconitate	rs12632521	29	3	32682166	A		0.920		1.3×10^-07^	0.667	0.219	*GLB1,TRIM71,TMPPE,CRTAP,SUSD5,CCR4*
Nucleotide	guanosine	rs112308097	27	2	230821995	T		NA		8.7×10^-08^	0.576	0.301	

*The SNP with the smallest (i.e., most significant) P value in each clumped region in the mtQTL analysis.

†The number of SNPs with a P value of < 1 × 10^-3^ in each clumped region in the mtQTL analysis.

‡We considered genetic loci with a posterior probability of H4 (PP.H4) of ≥0.5 to colocalize.

Alt, alternative; Chr, chromosome; CAAPA, consortium on asthma among African-ancestry populations in the Americas; DPA, docosapentaenoate; KB, kilobyte; GPE, glycerophosphorylethanolamine; GPG, glycerophosphoglycerol; PP.H4, posterior probability of H4; SNP, single nucleotide polymorphism; UKB, UK Biobank.

### Genetic loci are concordant to known asthma-susceptibility genes

**Figure 4:**
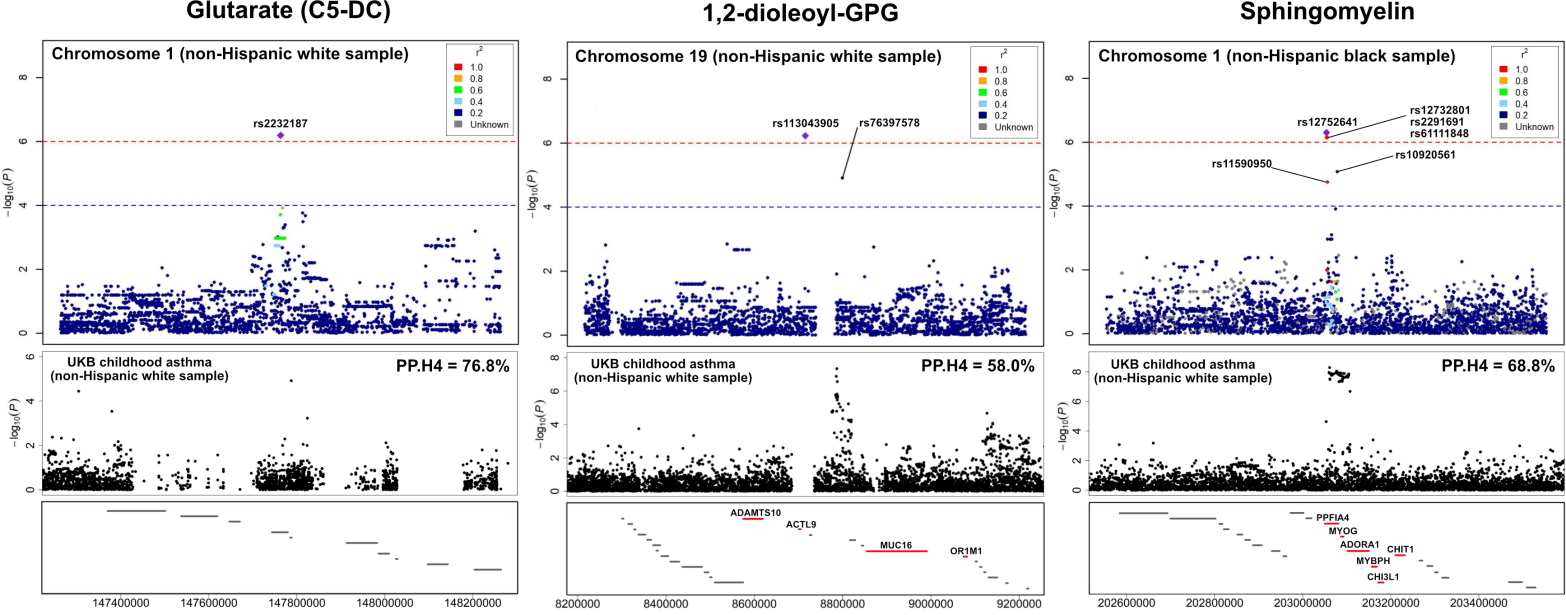
LocusZoom for the genetic loci with the UK Biobank summary statistics of childhood asthma To compare the association peaks of the loci between the mtQTL analysis in infants with bronchiolitis and the UK Biobank summary statistics for childhood asthma, we applied LocusZoom to glutarate (C5-DC: glutarylcarnitine) on chromosome 1q21, 1,2-dioleoyl-GPG on chromosome 19p13, and sphingomyelin [d17:1/16:0, d18:1/15:0, d16:1/17:0] on chromosome 1q32. The association peaks of these three loci were aligned with those in the same genetic regions of UK Biobank summary statistics for childhood asthma. The red lines at the bottom boxes represent the genetic location of the genes that are known to be related to asthma. There were concordances between the association peaks of two loci (i.e., chromosome 19p13 with 1,2-dioleoyl-GPG, chromosome 1q32 with sphingomyelin) and known asthma genes (e.g., *ADORA1*, *MUC16*). Abbreviations: GPG, glycero-3-phosphoglycerol; PP.H4, posterior probability of hypothesis 4; UKB, UK Biobank.

The association peaks for the 30 loci with colocalization evidence were compared between the mtQTL analysis and UK Biobank (**Figure S4**). For example, the association peaks of glutarate on chromosome 1q21 (e.g., rs2232187), 1,2-dioleoyl-GPG on chromosome 19p13 (e.g., rs113043905), and sphingomyelin [d17:1/16:0, d18:1/15:0, d16:1/17:0] on chromosome 1q32 (e.g., rs12752641) were aligned with the association peak in the same genetic region from the UK Biobank statistics of childhood asthma. Furthermore, there were apparent concordances between the association peaks for two loci (i.e., chromosome 19p13 with 1,2-dioleoyl-GPG, chromosome 1q32 with sphingomyelin [d17:1/16:0, d18:1/15:0, d16:1/17:0]) and genes that are known to be related to asthma (e.g., *MUC16*, *ADORA1*; **Figure 4**).  

## Discussion

By applying an integrated genetics-metabolomics approach to multicenter prospective cohort data of 744 infants with severe bronchiolitis, we identified 28 metabolites associated with asthma development and 349 independent genetic loci associated with these metabolites. Additionally, of these loci, colocalization analysis (with independent GWAS datasets for asthma) revealed 30 shared loci between 16 metabolites and asthma risk. Furthermore, the significant SNPs within two loci were aligned with known asthma-susceptibility genes (e.g., *ADORA1*, *MUC16*). To the best of our knowledge, this is the first study that has investigated infant bronchiolitis-childhood asthma link with an integrative genetics-metabolomics approach and demonstrated genetically driven metabolites and related genetic loci associated with asthma risk.

Childhood asthma is a heterogeneous syndrome that results from complex interactions between genetic and environmental factors in early childhood (7). Recent research has studied the mechanisms by applying high-throughput approaches to survey the metabolome—the downstream functional products of a child’s genetic make-up and environmental exposures (e.g., virus respiratory infection) (21, 31–40). Consistent with our findings, for example, recent studies of infants with acute respiratory infection have reported associations of the upper airway (21, 34–38), serum (36, 38), and urine (39) metabolome signature (e.g., altered sphingolipid, phospholipid, and fatty acid metabolism)—with the subsequent development of asthma. Independent from these metabolomics investigations, GWASs have identified genetic regions associated with respiratory syncytial virus (RSV) infection (41–43), severe bronchiolitis (41, 42, 44), and asthma risk (12–17, 43, 45–49). For example, multiple studies have identified *ORMDL3* as an asthma susceptibility gene (12, 15, 46, 48) with asthma risk. ORMDL3 is a major regulator of serine palmitoyltransferase—the rate-limiting enzyme of sphingolipid biosynthesis (50). While most research independently have applied genetics and metabolomics, few studies have examined the integrated relationships of genetic variants and altered metabolism with prevalent asthma in adults (51, 52). For example, Johnson et al. recently conducted an mtQTL analysis of 348 adults (59 with prevalent asthma) from Tangier Island and found several serum metabolites (e.g., linoleoyl ethanolamide) associated with asthma risk (51). Our multicenter prospective study—integrating the genome and nasopharyngeal airway metabolome data from high-risk infants—corroborates these earlier reports, and extends them by demonstrating genetic loci and genetically driven metabolites associated with the risk of developing asthma.

The exact mechanisms underlying the observed relationship of genetic loci and metabolites—e.g., genes within chromosome 1q32 (e.g., *ADORA1*, *PPFIA4*) and sphingomyelin (d17:1/16:0, d18:1/15:0, d16:1/17:0)—with asthma risk warrant further clarification. Studies have shown that genes on chromosome 1q32, including *ADORA1* and *PPFIA4* are associated with asthma risk (13, 53, 54), and *ADORA1* may interact with sphingolipids to enhance airway inflammation (55–59). For example, adenosine receptor A1 (ADORA1) encoded by the *ADORA1* gene contributes to bronchoconstriction, mucus secretion, and inflammation in bronchial epithelial cells through the sphingolipid signaling pathway (55, 56). Experimental studies have also shown that ADORA1 regulated cyclic adenosine monophosphate (cAMP) and sphingomyelin-derived lipids to mobilize intracellular calcium stores in bronchial smooth muscle cells, leading to the contraction of the bronchial smooth muscles and airway remodeling (57–59). Sphingolipids are not only integrated components of the human cell membrane (60) but also have molecular signaling functions with roles in the immune response to infections, inflammation, and cell proliferation, thereby contributing to asthma pathobiology (61). Accordingly, studies have suggested ADORA1 (62) and sphingolipids (63) as therapeutic targets for asthma.

In addition to sphingomyelins, we also observed a relationship between genes on chromosome 19p13 (e.g., *MUC16*), 1,2-dioleoyl-GPG, and asthma risk. 1,2-dioleoyl-GPG is one of the phosphatidylglycerols (PGs)—a main component of pulmonary surfactant (64). An experimental study has suggested that PG inhibits proinflammatory protein expression in alveolar macrophages through downregulation of NF-κB activation (65, 66). In contrast, the depletion of pulmonary surfactant PG leads to asthma-associated surfactant dysfunction (10). Another study has also reported that PG inhibits RSV infection by blocking viral attachment to epithelial cells (67). Besides, Mucin-16—also known as CA125 and encoded regulated by the *MUC16* gene—is the largest membrane-associated mucin synthesized in the endoplasmic reticulum (ER) of the bronchial epithelial cells (68–70). Research has demonstrated that the expression of mucin-16 was promoted by NF-κB activation and protects against ER stress (71). ER stress regulates proinflammatory signaling in epithelial cells through pulmonary surfactant dysfunction that is also induced by PG depletion (72). Accordingly, *MUC16* gene, mucin-16, and GPG jointly play roles in ER stress (73). Notwithstanding the complexity of these potential mechanisms, the identification of genetically driven metabolites associated with the development of childhood asthma is an important finding. Our data—in conjunction with the literature—should advance further research into the pathobiological mechanisms underlying the bronchiolitis-asthma link.

The current study has several potential limitations. First, the study did not have “healthy controls.” Yet, the objective of the study was not to identify the genetic loci and metabolites related to incident bronchiolitis (i.e., bronchiolitis yes vs. no) but to investigate the functional consequences of genetic risk factors for asthma (i.e., genetically driven metabolites) in this high-risk population. Second, compared to the non-analytic cohort, the analytic cohort had an overrepresentation of solo rhinovirus infection (3% vs. 7%), which might have led to selection bias. Third, it is possible that asthma diagnosis is misclassified and that some children are going to develop asthma at a later age. To address these potential limitations, the cohort is currently being followed up to age 9 years. In addition, in the current study, children who had asthma-related symptoms but did not receive asthma medication might have been over-diagnosed with asthma. Fourth, the sample size of the current analysis was relatively small, partially because of the stratified analysis across the different racial/ethnic subgroups. To accommodate the limited statistical power, we performed the mtQTL analyses with the significance threshold (P <1×10^-6^) proposed in a previous study (28). The statistical power of the analyses calculated using the GAS Power Calculator (74) was 0.72 in non-Hispanic white, 0.71 in non-Hispanic black, and 0.71 in Hispanics. Fifth, the CAAPA dataset provided summary statistics for an African-admixed population with asthma (26). Therefore, the sample from the other racial/ethnic groups might have affected the colocalization estimates of our non-Hispanic black sample due to population stratification (75). Sixth, the lack of publicly available asthma GWAS data in the Hispanic sample precluded us from conducting colocalization analysis for our Hispanic sample. Lastly, our inferences may not be generalizable to infants without severe bronchiolitis (i.e., infants with mild-to-moderate bronchiolitis). Nonetheless, our observations remain directly relevant to the 110,000 infants hospitalized annually in the U.S (1).—a large population with a substantial morbidity burden.

## Conclusions

By integrating the genetics and nasopharyngeal airway metabolomics data from a multicenter, prospective cohort study of infants hospitalized for bronchiolitis, we identified genetically driven metabolites (e.g., 1,2-dioleoyl-GPG, sphingomyelin) associated with asthma development and genetic loci associated with both these metabolites and asthma susceptibility genes (e.g., *ADORA1*, *MUC16*). These associations were also confirmed by colocalization analyses with independent GWAS datasets for asthma. Identifying these metabolites and genetic loci should advance research into the functional consequences of genetic risk factors for the development of asthma. Furthermore, these findings will, in turn, accelerate the understanding of the bronchiolitis-asthma link and the development of prevention strategies for childhood asthma. 

## Data availability statement

The data presented in the study are deposited in the ImmPort repository, accession number SDY2157.

## Ethics statement

The studies involving human participants were reviewed and approved by Alfred I. duPont Hospital for Children, Wilmington, DE; Arnold Palmer Hospital for Children, Orlando, FL; Boston Children’s Hospital, Boston, MA; Children’s Hospital of Los Angeles, Los Angeles, CA; Children’s Hospital of Philadelphia, Philadelphia, PA; Children’s Hospital of Pittsburgh, Pittsburgh, PA; The Children’s Hospital at St. Francis, Tulsa, OK; The Children’s Mercy Hospital & Clinics, Kansas City, MO; Children’s National Medical Center, Washington, D.C.; Cincinnati Children’s Hospital and Medical Center, Cincinnati, OH; Connecticut Children’s Medical Center, Hartford, CT; Dell Children’s Medical Center of Central Texas, Austin, TX; Norton Children’s Hospital, Louisville, KY; Massachusetts General Hospital, Boston, MA; Phoenix Children’s Hospital, Phoenix, AZ; Seattle Children’s Hospital, Seattle, WA; Texas Children’s Hospital, Houston, TX. Written informed consent to participate in this study was provided by the participants’ legal guardian/next of kin.

## Author contributions

TO carried out the main statistical analysis, drafted the initial manuscript, and approved the final manuscript as submitted. ZZ carried out the data processing, developed the methodology, analyzed and interpreted data, reviewed and revised the initial manuscript, and approved the final manuscript as submitted. LL, JC, BH, AH, ER, and RF collected the study data, reviewed and revised the initial manuscript, and approved the final manuscript as submitted. CC and KH conceptualized the study, obtained funding, supervised the statistical analysis, reviewed and revised the initial manuscript, and approved the final manuscript as submitted. All authors contributed to the article and approved the submitted version.

## References

[1] Fujiogi M Goto T Yasunaga H Fujishiro J Mansbach JM Camargo CA Jr. Trends in bronchiolitis hospitalizations in the united states: 2000-2016. Pediatrics (2019) 144:e20192614. doi: 10.1542/peds.2019-2614 31699829 PMC6889950

[2] Régnier SA Huels J. Association between respiratory syncytial virus hospitalizations in infants and respiratory sequelae: systematic review and meta-analysis. Pediatr Infect Dis J (2013) 32:820–6. doi: 10.1097/INF.0b013e31829061e8 23518824

[3] Koponen P Helminen M Paassilta M Luukkaala T Korppi M. Preschool asthma after bronchiolitis in infancy. Eur Respir J (2012) 39:76–80. doi: 10.1183/09031936.00040211 21700604

[4] Törmänen S Lauhkonen E Riikonen R Koponen P Huhtala H Helminen M. Risk factors for asthma after infant bronchiolitis. Allergy. (2018) 73:916–22. doi: 10.1111/all.13347 29105099

[5] Carroll KN Wu P Gebretsadik T Griffin MR Dupont WD Mitchel EF. The severity-dependent relationship of infant bronchiolitis on the risk and morbidity of early childhood asthma. J Allergy Clin Immunol (2009) 123:1055–61,1061.e1. doi: 10.1016/j.jaci.2009.02.021 19361850 PMC2703291

[6] Henderson J Hilliard TN Sherriff A Stalker D Al Shammari N Thomas HM. Hospitalization for RSV bronchiolitis before 12 months of age and subsequent asthma, atopy and wheeze: a longitudinal birth cohort study. Pediatr Allergy Immunol (2005) 16:386–92. doi: 10.1111/j.1399-3038.2005.00298.x 16101930

[7] Hernandez-Pacheco N Kere M Melén E. Gene-environment interactions in childhood asthma revisited; expanding the interaction concept. Pediatr Allergy Immunol (2022) 33:e13780. doi: 10.1111/pai.13780 35616899 PMC9325482

[8] Stewart CJ Mansbach JM Wong MC Ajami NJ Petrosino JF Camargo CA Jr. Associations of nasopharyngeal metabolome and microbiome with severity among infants with bronchiolitis. A multiomic analysis. Am J Respir Crit Care Med (2017) 196:882–91. doi: 10.1164/rccm.201701-0071OC PMC5649976 28530140

[9] Stewart CJ Mansbach JM Ajami NJ Petrosino JF Zhu Z Liang L. Serum metabolome is associated with the nasopharyngeal microbiota and disease severity among infants with bronchiolitis. J Infect Dis (2019) 219:2005–14. doi: 10.1093/infdis/jiz021 PMC6534192 30629185

[10] Hite RD Seeds MC Bowton DL Grier BL Safta AM Balkrishnan R. Surfactant phospholipid changes after antigen challenge: a role for phosphatidylglycerol in dysfunction. Am J Physiol Lung Cell Mol Physiol (2005) 288:L610–7. doi: 10.1152/ajplung.00273.2004 15347567

[11] Adams S Lopata AL Smuts CM Baatjies R Jeebhay MF. Relationship between serum omega-3 fatty acid and asthma endpoints. Int J Environ Res Public Health (2019) 16(1):43. doi: 10.3390/ijerph16010043 PMC6338947 30585204

[12] Moffatt MF Kabesch M Liang L Dixon AL Strachan D Heath S. Genetic variants regulating ORMDL3 expression contribute to the risk of childhood asthma. Nature. (2007) 448:470–3. doi: 10.1038/nature06014 17611496

[13] Pividori M Schoettler N Nicolae DL Ober C Im HK. Shared and distinct genetic risk factors for childhood-onset and adult-onset asthma: genome-wide and transcriptome-wide studies. Lancet Respir Med (2019) 7:509–22. doi: 10.1016/S2213-2600(19)30055-4 PMC6534440 31036433

[14] Cookson W Moffatt M Strachan DP. Genetic risks and childhood-onset asthma. J Allergy Clin Immunol (2011) 128:266–72. doi: 10.1016/j.jaci.2011.06.026 21807248

[15] Ferreira MAR Mathur R Vonk JM Szwajda A Brumpton B Granell R. Genetic architectures of childhood- and adult-onset asthma are partly distinct. Am J Hum Genet (2019) 104:665–84. doi: 10.1016/j.ajhg.2019.02.022 PMC6451732 30929738

[16] Bønnelykke K Sleiman P Nielsen K Kreiner-Møller E Mercader JM Belgrave D. A genome-wide association study identifies CDHR3 as a susceptibility locus for early childhood asthma with severe exacerbations. Nat Genet (2014) 46:51–5. doi: 10.1038/ng.2830 24241537

[17] Eliasen AU Pedersen CET Rasmussen MA Wang N Soverini M Fritz A. Genome-wide study of early and severe childhood asthma identifies interaction between CDHR3 and GSDMB. J Allergy Clin Immunol (2022) 150(3):622–30. doi: 10.1016/j.jaci.2022.03.019 35381269

[18] Hasegawa K Mansbach JM Bochkov YA Gern JE Piedra PA Bauer CS. Association of rhinovirus c bronchiolitis and immunoglobulin e sensitization during infancy with development of recurrent wheeze. JAMA Pediatr (2019) 173:544–52. doi: 10.1001/jamapediatrics.2019.0384 PMC6547078 30933255

[19] Ralston SL Lieberthal AS Meissner HC Alverson BK Baley JE Gadomski AM. Clinical practice guideline: the diagnosis, management, and prevention of bronchiolitis. Pediatrics. (2014) 134:e1474–502. doi: 10.1542/peds.2014-2742 25349312

[20] Taliun D Harris DN Kessler MD Carlson J Szpiech ZA Torres R. Sequencing of 53,831 diverse genomes from the NHLBI TOPMed program. Nature. (2021) 590:290–9. doi: 10.1038/s41586-021-03205-y PMC7875770 33568819

[21] Zhu Z Camargo CA Raita Y Fujiogi M Liang L Rhee EP. Metabolome subtyping of severe bronchiolitis in infancy and risk of childhood asthma. J Allergy Clin Immunol (2022) 149:102–12. doi: 10.1016/j.jaci.2021.05.036 PMC8660920 34119532

[22] Camargo CA Jr Ingham T Wickens K Thadhani R Silvers KM Epton MJ. Cord-blood 25-hydroxyvitamin d levels and risk of respiratory infection, wheezing, and asthma. Pediatrics. (2011) 127:e180–7. doi: 10.1542/peds.2010-0442 21187313

[23] Zhu Z Lee PH Chaffin MD Chung W Loh PR Lu Q. A genome-wide cross-trait analysis from UK biobank highlights the shared genetic architecture of asthma and allergic diseases. Nat Genet (2018) 50:857–64. doi: 10.1038/s41588-018-0121-0 PMC5980765 29785011

[24] Zhu Z Zhu X Liu CL Shi H Shen S Yang Y. Shared genetics of asthma and mental health disorders: a large-scale genome-wide cross-trait analysis. Eur Respir J (2019) 54:1901507. doi: 10.1183/13993003.01507-2019 31619474

[25] Zhu Z Guo Y Shi H Liu CL Panganiban RA Chung W. Shared genetic and experimental links between obesity-related traits and asthma subtypes in UK biobank. J Allergy Clin Immunol (2020) 145:537–49. doi: 10.1016/j.jaci.2019.09.035 PMC7010560 31669095

[26] Daya M Rafaels N Brunetti TM Chavan S Levin AM Shetty A. Association study in African-admixed populations across the americas recapitulates asthma risk loci in non-African populations. Nat Commun (2019) 10:880. doi: 10.1038/s41467-019-08469-7 30787307 PMC6382865

[27] Pruim RJ Welch RP Sanna S Teslovich TM Chines PS Gliedt TP. LocusZoom: regional visualization of genome-wide association scan results. Bioinformatics. (2010) 26:2336–7. doi: 10.1093/bioinformatics/btq419 PMC2935401 20634204

[28] Duggal P Gillanders EM Holmes TN Bailey-Wilson JE. Establishing an adjusted p-value threshold to control the family-wide type 1 error in genome wide association studies. BMC Genomics (2008) 9:516. doi: 10.1186/1471-2164-9-516 18976480 PMC2621212

[29] Makrinioti H Bush A Gern J Johnston SL Papadopoulos N Feleszko W. The role of interferons in driving susceptibility to asthma following bronchiolitis: controversies and research gaps. Front Immunol (2021) 12:761660. doi: 10.3389/fimmu.2021.761660 34925333 PMC8677668

[30] Read JF Bosco A. Decoding susceptibility to respiratory viral infections and asthma inception in children. Int J Mol Sci (2020) 21:6372. doi: 10.3390/ijms21176372 32887352 PMC7503410

[31] Zhu Z Camargo CA Jr Hasegawa K. Metabolomics in the prevention and management of asthma. Expert Rev Respir Med (2019) 13:1135–8. doi: 10.1080/17476348.2019.1674650 PMC6854291 31561725

[32] Kelly RS Dahlin A McGeachie MJ Qiu W Sordillo J Wan ES. Asthma metabolomics and the potential for integrative omics in research and the clinic. Chest. (2017) 151:262–77. doi: 10.1016/j.chest.2016.10.008 PMC5310123 27776981

[33] Rago D Pedersen CET Huang M Kelly RS Gürdeniz G Brustad N. Characteristics and mechanisms of a sphingolipid-associated childhood asthma endotype. Am J Respir Crit Care Med (2021) 203:853–63. doi: 10.1164/rccm.202008-3206OC PMC8017574 33535020

[34] Raita Y Pérez-Losada M Freishtat RJ Harmon B Mansbach JM Piedra PA. Integrated omics endotyping of infants with respiratory syncytial virus bronchiolitis and risk of childhood asthma. Nat Commun (2021) 12:3601. doi: 10.1038/s41467-021-23859-6 34127671 PMC8203688

[35] Raita Y Camargo CA Jr Bochkov YA Celedón JC Gern JE Mansbach JM. Integrated-omics endotyping of infants with rhinovirus bronchiolitis and risk of childhood asthma. J Allergy Clin Immunol (2021) 147:2108–17. doi: 10.1016/j.jaci.2020.11.002 PMC8116357 33197460

[36] Fujiogi M Camargo CA Jr Raita Y Zhu Z Celedón JC Mansbach JM. Integrated associations of nasopharyngeal and serum metabolome with bronchiolitis severity and asthma: A multicenter prospective cohort study. Pediatr Allergy Immunol (2021) 32:905–16. doi: 10.1111/pai.13466 PMC8269431 33559342

[37] Fujiogi M Zhu Z Raita Y Ooka T Celedon JC Freishtat R. Nasopharyngeal lipidomic endotypes of infants with bronchiolitis and risk of childhood asthma: a multicentre prospective study. Thorax (2022) 77:1059–69. doi: 10.1136/thorax-2022-219016. thoraxjnl-2022-219016. PMC10329482 35907638

[38] Kyo M Zhu Z Nanishi M Shibata R Ooka T Freishtat RJ. Association of nasopharyngeal and serum glutathione metabolism with bronchiolitis severity and asthma risk: A prospective multicenter cohort study. Metabolites (2022) 12:674. doi: 10.3390/metabo12080674 35893241 PMC9394245

[39] Turi KN Romick-Rosendale L Gebretsadik T Watanabe M Brunwasser S Anderson LJ. Using urine metabolomics to understand the pathogenesis of infant respiratory syncytial virus (RSV) infection and its role in childhood wheezing. Metabolomics. (2018) 14:135. doi: 10.1007/s11306-018-1431-z 30830453 PMC6557166

[40] Carraro S Bozzetto S Giordano G El Mazloum D Stocchero M Pirillo P. Wheezing preschool children with early-onset asthma reveal a specific metabolomic profile. Pediatr Allergy Immunol (2018) 29:375–82. doi: 10.1111/pai.12879 29468750

[41] Janssen R Bont L Siezen CLE Hodemaekers HM Ermers MJ Doornbos G. Genetic susceptibility to respiratory syncytial virus bronchiolitis is predominantly associated with innate immune genes. J Infect Dis (2007) 196:826–34. doi: 10.1086/520886 17703412

[42] Forton JT Rowlands K Rockett K Hanchard N Herbert M Kwiatkowski DP. Genetic association study for RSV bronchiolitis in infancy at the 5q31 cytokine cluster. Thorax. (2009) 64:345–52. doi: 10.1136/thx.2008.102111 PMC3015100 19131452

[43] Larkin EK Hartert TV. Genes associated with RSV lower respiratory tract infection and asthma: the application of genetic epidemiological methods to understand causality. Future Virol (2015) 10:883–97. doi: 10.2217/fvl.15.55 PMC4603287 26478738

[44] Pasanen A Karjalainen MK Bont L Piippo-Savolainen E Ruotsalainen M Goksör E. Genome-wide association study of polymorphisms predisposing to bronchiolitis. Sci Rep (2017) 7:41653. doi: 10.1038/srep41653 28139761 PMC5282585

[45] Moffatt MF Gut IG Demenais F Strachan DP Bouzigon E Heath S. A large-scale, consortium-based genomewide association study of asthma. N Engl J Med (2010) 363:1211–21. doi: 10.1056/NEJMoa0906312 PMC4260321 20860503

[46] Demenais F Margaritte-Jeannin P Barnes KC Cookson WOC Altmüller J Ang W. Multiancestry association study identifies new asthma risk loci that colocalize with immune-cell enhancer marks. Nat Genet (2018) 50:42–53. doi: 10.1038/s41588-017-0014-7 29273806 PMC5901974

[47] Miller RL Grayson MH Strothman K. Advances in asthma: New understandings of asthma’s natural history, risk factors, underlying mechanisms, and clinical management. J Allergy Clin Immunol (2021) 148:1430–41. doi: 10.1016/j.jaci.2021.10.001 34655640

[48] Galanter J Choudhry S Eng C Nazario S Rodríguez-Santana JR Casal J. ORMDL3 gene is associated with asthma in three ethnically diverse populations. Am J Respir Crit Care Med (2008) 177:1194–200. doi: 10.1164/rccm.200711-1644OC PMC2408437 18310477

[49] Chang X March M Mentch F Qu H Liu Y Glessner J. Genetic architecture of asthma in African American patients. J Allergy Clin Immunol (2022). doi: 10.1016/j.jaci.2022.09.001 PMC9992439 36089080

[50] James B Milstien S Spiegel S. ORMDL3 and allergic asthma: From physiology to pathology. J Allergy Clin Immunol (2019) 144:634–40. doi: 10.1016/j.jaci.2019.07.023 PMC6910079 31376405

[51] Johnson RK Brunetti T Quinn K Doenges K Campbell M Arehart C. Discovering metabolite quantitative trait loci in asthma using an isolated population. J Allergy Clin Immunol (2022) 149:1807–1811.e16. doi: 10.1016/j.jaci.2021.11.002 34780848 PMC9081120

[52] Ried JS Baurecht H Stückler F Krumsiek J Gieger C Heinrich J. Integrative genetic and metabolite profiling analysis suggests altered phosphatidylcholine metabolism in asthma. Allergy. (2013) 68:629–36. doi: 10.1111/all.12110 23452035

[53] Rathcke CN Holmkvist J Husmoen LLN Hansen T Pedersen O Vestergaard H. Association of polymorphisms of the CHI3L1 gene with asthma and atopy: a populations-based study of 6514 Danish adults. PloS One (2009) 4:e6106. doi: 10.1371/journal.pone.0006106 19568425 PMC2699472

[54] March ME Sleiman PM Hakonarson H. Genetic polymorphisms and associated susceptibility to asthma. Int J Gen Med (2013) 6:253–65. doi: 10.2147/IJGM.S28156 PMC3636804 23637549

[55] Kanehisa M Goto S. KEGG: kyoto encyclopedia of genes and genomes. Nucleic Acids Res (2000) 28:27–30. doi: 10.1093/nar/28.1.27 10592173 PMC102409

[56] Brown RA Spina D Page CP. Adenosine receptors and asthma. Br J Pharmacol (2008) 153 Suppl 1:S446–56. doi: 10.1038/bjp.2008.22 PMC2268070 18311158

[57] Pyne S Pyne NJ. The differential regulation of cyclic AMP by sphingomyelin-derived lipids and the modulation of sphingolipid-stimulated extracellular signal regulated kinase-2 in airway smooth muscle. Biochem J (1996) 315:917–23. doi: 10.1042/bj3150917 PMC1217294 8645177

[58] Billington CK Ojo OO Penn RB Ito S. cAMP regulation of airway smooth muscle function. Pulm Pharmacol Ther (2013) 26:112–20. doi: 10.1016/j.pupt.2012.05.007 PMC3574867 22634112

[59] Ethier MF Madison JM. Adenosine A1 receptors mediate mobilization of calcium in human bronchial smooth muscle cells. Am J Respir Cell Mol Biol (2006) 35:496–502. doi: 10.1165/rcmb.2005-0290OC 16709961 PMC2065849

[60] Gault CR Obeid LM Hannun YA. An overview of sphingolipid metabolism: from synthesis to breakdown. Adv Exp Med Biol (2010) 688:1–23. doi: 10.1007/978-1-4419-6741-1_1 20919643 PMC3069696

[61] Maceyka M Spiegel S. Sphingolipid metabolites in inflammatory disease. Nature. (2014) 510:58–67. doi: 10.1038/nature13475 24899305 PMC4320971

[62] Adikusuma W Chou WH Lin MR Ting J Irham LM Perwitasari DA. Identification of druggable genes for asthma by integrated genomic network analysis. Biomedicines. (2022) 10(1):113. doi: 10.3390/biomedicines10010113 35052792 PMC8773254

[63] Sturgill JL. Sphingolipids and their enigmatic role in asthma. Adv Biol Regul (2018) 70:74–81. doi: 10.1016/j.jbior.2018.09.001 30197277 PMC6560640

[64] Liu Q Guan J Song R Zhang X Mao S. Physicochemical properties of nanoparticles affecting their fate and the physiological function of pulmonary surfactants. Acta Biomater. (2022) 140:76–87. doi: 10.1016/j.actbio.2021.11.034 34843949

[65] Choudhary V Uaratanawong R Patel RR Patel H Bao W Hartney B. Phosphatidylglycerol inhibits toll-like receptor-mediated inflammation by danger-associated molecular patterns. J Invest Dermatol (2019) 139:868–77. doi: 10.1016/j.jid.2018.10.021 PMC7309510 30391260

[66] Wu YZ Medjane S Chabot S Kubrusly FS Raw I Chignard M. Surfactant protein-a and phosphatidylglycerol suppress type IIA phospholipase A2 synthesis *via* nuclear factor-kappaB. Am J Respir Crit Care Med (2003) 168:692–9. doi: 10.1164/rccm.200304-467OC 12882758

[67] Numata M Nagashima Y Moore ML Berry KZ Chan M Kandasamy P. Phosphatidylglycerol provides short-term prophylaxis against respiratory syncytial virus infection. J Lipid Res (2013) 54:2133–43. doi: 10.1194/jlr.M037077 PMC3708363 23749985

[68] Blalock TD Spurr-Michaud SJ Tisdale AS Heimer SR Gilmore MS Ramesh V. Functions of MUC16 in corneal epithelial cells. Invest Ophthalmol Vis Sci (2007) 48:4509–18. doi: 10.1167/iovs.07-0430 17898272

[69] Davies JR Kirkham S Svitacheva N Thornton DJ Carlstedt I. MUC16 is produced in tracheal surface epithelium and submucosal glands and is present in secretions from normal human airway and cultured bronchial epithelial cells. Int J Biochem Cell Biol (2007) 39:1943–54. doi: 10.1016/j.biocel.2007.05.013 17604678

[70] Das S Batra SK. Understanding the unique attributes of MUC16 (CA125): Potential implications in targeted therapy. Cancer Res (2015) 75:4669–74. doi: 10.1158/0008-5472.CAN-15-1050 PMC4651718 26527287

[71] Lei Y Zang R Lu Z Zhang G Huang J Liu C. ERO1L promotes IL6/sIL6R signaling and regulates MUC16 expression to promote CA125 secretion and the metastasis of lung cancer cells. Cell Death Dis (2020) 11:853. doi: 10.1038/s41419-020-03067-8 33056994 PMC7560734

[72] Maguire JA Mulugeta S Beers MF. Endoplasmic reticulum stress induced by surfactant protein c BRICHOS mutants promotes proinflammatory signaling by epithelial cells. Am J Respir Cell Mol Biol (2011) 44:404–14. doi: 10.1165/rcmb.2009-0382OC PMC3095939 20463293

[73] Kim SR Kim DI Kang MR Lee KS Park SY Jeong JS. Endoplasmic reticulum stress influences bronchial asthma pathogenesis by modulating nuclear factor κB activation. J Allergy Clin Immunol (2013) 132:1397–408. doi: 10.1016/j.jaci.2013.08.041 24161747

[74] Johnson JL Abecasis GR. GAS power calculator: web-based power calculator for genetic association studies. BioRxiv (2017) 2017:164343. doi: 10.1101/164343

[75] Zhu Z Hasegawa K Camargo CA Liang L. Investigating asthma heterogeneity through shared and distinct genetics: Insights from genome-wide cross-trait analysis. J Allergy Clin Immunol (2021) 147:796–807. doi: 10.1016/j.jaci.2020.07.004 32693092 PMC7368660

