# Supplementary material for: Integrative genetics-metabolomics analysis of infant bronchiolitis-childhood asthma link: A multicenter prospective study
Source: Front Immunol. 2023 Feb 2;13:1111723. doi: 10.3389/fimmu.2022.1111723 (PMC9936313; doi:10.3389/fimmu.2022.1111723)
Supplement: Supplementary file 1 [file DataSheet_1.pdf]

SUPPLEMENTARY MATERIAL

Integrative genetics-metabolomics analysis of infant bronchiolitis-childhood asthma link: A multicenter prospective study

Tadao Ooka, MD, PhD; Zhaozhong Zhu, ScD; Liming Liang, PhD; Juan C. Celedon, MD, DrPH, FAAAAI; Brennan Harmon, MA; Andrea Hahn, MD, MS; Eugene P. Rhee, MD; Robert J. Freishtat, MD, MPH; Carlos A. Camargo Jr., MD, DrPH, FAAAAI; and Kohei Hasegawa, MD, MPH, PhD

Contents

Supplementary Methods 2

Supplementary Reference 9

Table S1. 12

Table S2. 13

Table S3. 15

Table S4. 17

Table S5. 40

Table S6. 52

Figure S1. 56

Figure S2. 74

Figure S3. 75

Figure S4. 76

SUPPLEMENTAL METHODS

Study Design, Setting, and Participants

We analyzed data from the 35th Multicenter Airway Research Collaboration (MARC-35)—a multicentre prospective cohort study of infants hospitalized with bronchiolitis (i.e., severe bronchiolitis).[1] MARC-35 is coordinated by the Emergency Medicine Network (EMNet), a collaboration of 247 participating hospitals.[2] Investigators enrolled infants (age <1 year) hospitalized with bronchiolitis at 17 sites across 14 U.S. states (**Table S1**) using a standardized protocol during three consecutive bronchiolitis seasons (from November 1 through April 30) during 2011-2014. Bronchiolitis was defined by the American Academy of Pediatrics guidelines[3] as an acute respiratory illness with some combination of rhinitis, cough, tachypnoea, wheezing, crackles, and retractions[3] and was diagnosed by an attending physician. We excluded infants who were transferred to a participating hospital >24 hours after the original hospitalization, those who consented >24 hours after hospitalization, or those with a known heart-lung disease, immunodeficiency, immunosuppression, or gestational age of <32 weeks. All patients were treated at the discretion of the treating physicians.

Of 1,016 infants enrolled in the MARC-35 cohort, the current analysis investigated 744 infants who were selected both for genotyping and nasopharyngeal metabolites profiling (**Table S2**). The institutional review board at each participating hospital approved the study with written informed consent obtained from the parent or guardian.

Data Collection

Clinical data (parents' demographic characteristics, medical, environmental, and family history, and details of the acute illness) were collected via structured interviews and chart reviews using a standardized

protocol.[4] All data were reviewed at the EMNet Coordinating Center (Boston, Massachusetts, USA), and site investigators were queried about missing data and discrepancies identified by manual data checks.

In addition to the clinical data, investigators also collected nasopharyngeal specimens for metabolome profiling within 24 hours of hospitalization by using standardized protocols.[4,5] All sites used the same collection equipment and collected the samples within 24 hours of a child's arrival in the medical ward or intensive care unit. Frozen nasopharyngeal specimens were shipped in batches to Baylor College of Medicine (Houston, Texas, USA) where they were tested for respiratory viruses using real-time polymerase chain reaction (RT-PCR).[4–7] Besides, whole blood specimens for genotyping were collected at age 1 year.

Respiratory Virus Testing

Nasopharyngeal samples were tested for 17 respiratory viruses (including respiratory syncytial virus [RSV] and rhinovirus [RV]) by using RT-PCR assays.[7] For RV detection, complementary DNA was generated using virus-specific primers for RV and singleplex RT-PCR was used. The details of the RV primers and probes have been described elsewhere.[8]

Genotype imputation and quality control

We used the TOPMed reference panel[9] on the Michigan Imputation Server for genotype imputation.[10] More details about the imputation can be found here: https://imputationserver.readthedocs.io/en/latest/pipeline/. We applied the following steps to convert the genotype data into the format required for the imputation process:

1. Create variant call format (VCF) files, with genotypes split by each individual autosomal chromosome, strand ambiguous and monomorphic SNPs removed, and hg19 coordinates converted to hg38 coordinates. Upload these VCF files to the imputation server for quality control (QC) checks against the TOPMed reference panel.

2. Update the VCF files from step 1 by flipping the strands of variants identified by the imputation server QC checks as strand flipped. Upload these files to the imputation server for imputation against the TOPMed reference panel.

Before association testing, we removed the variants with Rsq (estimated r2, specific to each SNP) of <0.6 were removed from the imputed data set. Since the minor allele frequency (MAF) provided in the imputed data set does not account for relatedness between individuals, we recalculated the MAFs of variants by running a dummy association analysis using the R Bioconductor GENESIS package. We removed rare variants with a reestimated MAF of <0.01 from the dataset before association testing. The MAF was calculated based on the full cohort (i.e., all combined racial/ethnic samples) to remove rare variants (i.e., MAF<0.01). In addition, we performed QC standards for retained variants. We used PLINK to check the sex discrepancy between the phenotype and genetic data. We found five samples whose sex are potentially mismatched using PLINK default settings (F estimates of <0.2 yield female calls, and values of >0.8 yield male calls). We removed these five samples from the genetic analyses. We also selected variants that did not deviate from Hardy-Weinberg equilibrium (HWE) ($P > 1\times10^{-6}$), per variant missing call rates of <5%, and per-sample missing rate of <40%. Quantile-quantile plots were produced and checked for each phenotype. We retained imputed autosomal allele dosages of 10,852,874 variants with high imputation accuracy (Rsq ≥0.6) and appreciable MAFs (≥0.01) and used them for association testing.

Nasopharyngeal Airway Metabolome Profiling

Metabolome profiling used 125 μl of nasopharyngeal airway sample. All samples were blinded to Metabolon and processed in random order. The metabolic profiling used ACQUITY ultra-high performance liquid chromatography (UPLC) (Waters, Milford, MA, USA) and Q-Exactive high resolution/accurate mass spectrometry (MS) interfaced with a heated electrospray ionization (HESI-II) source and Orbitrap mass analyzer operated at 35,000 mass resolution (Thermo Fisher Scientific, Waltham, MA, USA).

Sample preparation was carried out as described previously.[11] In brief, recovery standards were added prior to the first step in the extraction process for quality control purposes. Proteins were precipitated with methanol under vigorous shaking for two minutes (Glen Mills Genogrinder 2000; Clifton, NJ, USA) followed by centrifugation. The resulting extract was divided into four fractions. The first aliquot was analyzed using acidic positive ion conditions, chromatographically optimized for more hydrophilic compounds. The second aliquot was also analyzed using acidic positive ion conditions and was chromatographically optimized for more hydrophobic compounds. The third aliquot was analyzed with basic negative ion optimized conditions using a separate dedicated C18 column. The fourth aliquot was analyzed via negative ionization following elution from a HILIC column (Waters UPLC BEH Amide). The MS analysis alternated between MS and data-dependent MS^n scans using dynamic exclusion.

Metabolites were identified by automated comparison of the ion features in the experimental samples to a reference library of chemical standard entries that include retention time, molecular weight (m/z), preferred adducts, and in-source fragments as well as associated MS spectra, and curated by visual inspection for quality control using QUICS software.[12] Identification of known chemical entities was based on comparisons to metabolomic library entries of >3,000 purified standards. Peaks were quantified using area-under-the-curve. The raw area counts for each metabolite in each sample were normalized to correct for variation due to instrument inter-day tuning differences by the median value for each run-day, setting the median to 1.0 for each run. Missing values were imputed with the observed minimum for that particular compound.

Four types of quality controls were analyzed in concert with the specimens: 1) samples generated from a pool from a small portion of each experimental specimen that served as technical replicate; 2) extracted water samples that served as process blanks; 3) samples of solvent used in extraction; and 4) a cocktail of standards spiked into every analyzed specimen that allowed instrument performance monitoring. The median relative standard deviation (RSD) for the standards that are added to each sample—a measure of instrument variability—was <5%.

Clinical outcome measure

The development of asthma by age 6 years was used as a clinical outcome in the analysis to identify asthma-related metabolites. Asthma was defined using a commonly used epidemiologic definition: physician-diagnosis of asthma, with either asthma medication use (e.g., albuterol inhaler, inhaled corticosteroids) or asthma-related symptoms (e.g., wheezing, nocturnal cough) in the preceding year.[13]

Statistical Analysis

Metabolites selection

Before the main analyses, we adjusted the metabolome data for potential batch effect by using empirical Bayes models (ComBat method)[14] and examined the performance of eight normalization methods (i.e., log-transformation, quantile[15], upper quartile[16], total sum scaling[17], trimmed mean of the M-values[18], relative log expression[19], cumulative sum scaling[17], probabilistic quotient normalization [PQN][20]) by comparing the coefficient of variation in QC samples after normalization. On the basis of this analysis, we selected PQN[20] as the best normalization method for the metabolites selection analysis. Besides, considering the characteristics of each normalization method, we adopted the rank based inverse transformation (INT)[21] in the metabolitequantitative trait loci (mtQTL) analysis.

As part of MARC-35, the current study analyzed the data of 744 infants who underwent both genotyping and nasopharyngeal metabolome profiling. We identified metabolite candidates for the mtQTL analysis by applying Lasso regression[22] on 283 nasopharyngeal metabolomes (features) and asthma outcome (label) data of the infants. We repeated the model 100 times and adopted metabolite as a candidate if the metabolite was selected as a feature of the Lasso regression model more than 30 times in total. We conducted 10-fold cross validation in each regression procedure, and selected the optimal lambda value by *cv.glmnet* function of *glmnet* package.

Metabolite quantitative trait loci (mtQTL) analysis

We performed additive logistic regression genetic association analyses adjusting for age, sex and two ancestry principal components using PLINK 1.90[23] to examine the association between the 28 candidate metabolites and genotypes in each racial/ethnic group. After the association analysis, we applied the PLINK clumping function (parameters: --clump-p1 1e-6 --clump-p2 1e-3 --clump-r2 0.2 --clump-kb 500) to determine mtQTLs that are independent of each other—i.e., variants with a P value of $<1 \times 10^{-3}$, R2 of ≥0.2 and <500 kb away from the peak were assigned to that peak's clump. To accommodate the limited statistical power, we have set the first p-threshold (--clump-p1) as 1×10^{-6} with reference to the significant level of the current study ($P<1\times10^{-6}$), and we set the second p-threshold (--clump-p2) as 1×10^{-3} according to the magnitude difference in the PLINK default setting. We used the GWAS Catalog (https://www.ebi.ac.uk/gwas, search date: August 22, 2022) to identify previously reported genes by the GWAS literature and to select the known genes related to asthma risk. We used WebGestalt (WEB-based Gene SeT AnaLysis Toolkit; http://www.webgestalt.org/, search data: July 1, 2022) for a functional enrichment (pathway) analysis.[24] We conducted over-representation analyses with the Reactome pathway enrichment category by using all genes within a clumped region in the mtQTL analysis in each of the three racial/ethnic subgroups.

Colocalization analyses

We carried forward the significant loci from the mtQTL analysis for Bayesian colocalization analysis with asthma using the *coloc R* package.[25] We conducted the Bayesian colocalization analysis by comparing the GWAS summary statistics between the mtQTL analysis in MARC-35, the UK Biobank (Non-Hispanic white sample with childhood asthma[26–28]), and the Consortium on Asthma among African-ancestry Populations in the Americas (CAAPA; Non-Hispanic black sample with a history of asthma[29]). We used the UK Biobank dataset for examining the statistics of the non-Hispanic white samples and used the CAAPA dataset for the non-Hispanic black sample. We extracted summary association data for variants within 500 kb of the index SNP at each of the 281 shared loci and estimated the probability that the two traits (i.e., metabolites and asthma) share

one common causal variant (PP.H4). Loci with a probability of ≥0.5 were considered to colocalize. The prior probability that an SNP was associated with both traits was set at $P=10^{-3}$. We also calculated pairwise linkage disequilibrium (LD) between the peak SNP and other SNPs in the loci with colocalization evidence (i.e., PP.H4 ≥0.5) for plotting locus zoom by using the PLINK LD calculation function.[23]

SUPPLEMENTARY REFERENCES

1. Hasegawa K, Mansbach JM, Bochkov YA, Gern JE, Piedra PA, Bauer CS, et al. Association of rhinovirus C bronchiolitis and immunoglobulin E sensitization during infancy with development of recurrent wheeze. *JAMA Pediatr*. 2019;173:544–52.
2. Emergency Medicine Network [Internet]. [cited 2022 Jul 1]. Available from: http://www.emnet-usa.org/
3. Ralston SL, Lieberthal AS, Meissner HC, Alverson BK, Baley JE, Gadomski AM, et al. Clinical practice guideline: the diagnosis, management, and prevention of bronchiolitis. *Pediatrics*. 2014;134:e1474-502.
4. Hasegawa K, Mansbach JM, Ajami NJ, Espinola JA, Henke DM, Petrosino JF, et al. Association of nasopharyngeal microbiota profiles with bronchiolitis severity in infants hospitalised for bronchiolitis. *Eur Respir J.* 2016;48:1329–39.
5. Hasegawa K, Jartti T, Mansbach JM, Laham FR, Jewell AM, Espinola JA, et al. Respiratory syncytial virus genomic load and disease severity among children hospitalized with bronchiolitis: multicenter cohort studies in the United States and Finland. *J Infect Dis.* 2015;211:1550–9.
6. Mansbach JM, Piedra PA, Teach SJ, Sullivan AF, Forgey T, Clark S, et al. Prospective multicenter study of viral etiology and hospital length of stay in children with severe bronchiolitis. *Arch Pediatr Adolesc Med.* 2012;166:700–6.
7. Mansbach JM, Piedra PA, Stevenson MD, Sullivan AF, Forgey TF, Clark S, et al. Prospective multicenter study of children with bronchiolitis requiring mechanical ventilation. *Pediatrics.* 2012;130:e492-500.
8. Lu X, Holloway B, Dare RK, Kuypers J, Yagi S, Williams JV, et al. Real-time reverse transcription-PCR assay for comprehensive detection of human rhinoviruses. *J Clin Microbiol.* 2008;46:533–9.
9. Taliun D, Harris DN, Kessler MD, Carlson J, Szpiech ZA, Torres R, et al. Sequencing of 53,831 diverse genomes from the NHLBI TOPMed Program. *Nature.* 2021;590:290–9.
10. Das S, Forer L, Schönherr S, Sidore C, Locke AE, Kwong A, et al. Next-generation genotype imputation service and methods. *Nat Genet.* 2016;48:1284–7.

11. Evans AM, DeHaven CD, Barrett T, Mitchell M, Milgram E. Integrated, nontargeted ultrahigh performance liquid chromatography/electrospray ionization tandem mass spectrometry platform for the identification and relative quantification of the small-molecule complement of biological systems. *Anal Chem.* 2009;81:6656–67.
12. Dehaven CD, Evans AM, Dai H, Lawton KA. Organization of GC/MS and LC/MS metabolomics data into chemical libraries. *J Cheminform.* 2010;2:9.
13. Camargo CA Jr, Ingham T, Wickens K, Thadhani R, Silvers KM, Epton MJ, et al. Cord-blood 25-hydroxyvitamin D levels and risk of respiratory infection, wheezing, and asthma. *Pediatrics.* 2011;127:e180-7.
14. Leek JT, Johnson WE, Parker HS, Jaffe AE, Storey JD. The sva package for removing batch effects and other unwanted variation in high-throughput experiments. *Bioinformatics.* 2012;28:882–3.
15. Zhao Y, Wong L, Goh WWB. How to do quantile normalization correctly for gene expression data analyses. *Sci Rep.* 2020;10:15534.
16. Evans C, Hardin J, Stoebel DM. Selecting between-sample RNA-Seq normalization methods from the perspective of their assumptions. *Brief Bioinform.* 2018;19:776–92.
17. Paulson JN, Stine OC, Bravo HC, Pop M. Differential abundance analysis for microbial marker-gene surveys. *Nat Methods.* 2013;10:1200–2.
18. Robinson MD, Oshlack A. A scaling normalization method for differential expression analysis of RNA-seq data. *Genome Biol.* 2010;11:R25.
19. Anders S, Huber W. Differential expression analysis for sequence count data. *Genome Biol.* 2010;11:R106.
20. Dieterle F, Ross A, Schlotterbeck G, Senn H. Probabilistic quotient normalization as robust method to account for dilution of complex biological mixtures. Application in 1H NMR metabonomics. *Anal Chem.* 2006;78:4281–90.

21. McCaw ZR, Lane JM, Saxena R, Redline S, Lin X. Operating characteristics of the rank-based inverse normal transformation for quantitative trait analysis in genome-wide association studies. *Biometrics.* 2020;76:1262–72.
22. Ranstam J, Cook JA. LASSO regression. *Br J Surg.* 2018;105:1348–1348.
23. Purcell S, Neale B, Todd-Brown K, Thomas L, Ferreira MAR, Bender D, et al. PLINK: a tool set for whole-genome association and population-based linkage analyses. *Am J Hum Genet.* 2007;81:559–75.
24. Liao Y, Wang J, Jaehnig EJ, Shi Z, Zhang B. WebGestalt 2019: gene set analysis toolkit with revamped UIs and APIs. *Nucleic Acids Res.* 2019;47:W199–205.
25. Giambartolomei C, Vukcevic D, Schadt EE, Franke L, Hingorani AD, Wallace C, et al. Bayesian test for colocalisation between pairs of genetic association studies using summary statistics. *PLoS Genet.* 2014;10:e1004383.
26. Zhu Z, Lee PH, Chaffin MD, Chung W, Loh PR, Lu Q, et al. A genome-wide cross-trait analysis from UK Biobank highlights the shared genetic architecture of asthma and allergic diseases. *Nat Genet.* 2018;50:857–64.
27. Zhu Z, Zhu X, Liu CL, Shi H, Shen S, Yang Y, et al. Shared genetics of asthma and mental health disorders: a large-scale genome-wide cross-trait analysis. *Eur Respir J.* 2019;54(6):1901507.
28. Zhu Z, Guo Y, Shi H, Liu CL, Panganiban RA, Chung W, et al. Shared genetic and experimental links between obesity-related traits and asthma subtypes in UK Biobank. *J Allergy Clin Immunol.* 2020;145:537–49.
29. Daya M, Rafaels N, Brunetti TM, Chavan S, Levin AM, Shetty A, et al. Association study in African-admixed populations across the Americas recapitulates asthma risk loci in non-African populations. *Nat Commun.* 2019;10:880.

Table S1. Principal investigators at the 17 participating sites in MARC-35

Amy D. Thompson, MD	Alfred I. duPont Hospital for Children, Wilmington, DE
Federico R. Laham, MD, MS	Arnold Palmer Hospital for Children, Orlando, FL
Jonathan M. Mansbach, MD, MPH	Boston Children's Hospital, Boston, MA
Vincent J. Wang, MD, MHA and Susan Wu, MD	Children's Hospital of Los Angeles, Los Angeles, CA
Michelle B. Dunn, MD and Jonathan M.Spergel, MD, PhD	Children's Hospital of Philadelphia, Philadelphia, PA
Juan C. Celedón, MD, DrPH	Children's Hospital of Pittsburgh, Pittsburgh, PA
Michael R. Gomez, MD, MS-HCA and Nancy Inhofe, MD	The Children's Hospital at St. Francis, Tulsa, OK
Brian M. Pate, MD and Henry T. Puls, MD	The Children's Mercy Hospital & Clinics, Kansas City, MO
Stephen J. Teach, MD, MPH	Children's National Medical Center, Washington, D.C.
Richard T. Strait, MD and Stephen C. Porter, MD, MSc, MPH	Cincinnati Children's Hospital and Medical Center, Cincinnati, OH
Ilana Y. Waynik, MD	Connecticut Children's Medical Center, Hartford, CT
Sujit Iyer, MD	Dell Children's Medical Center of Central Texas, Austin, TX
Michelle D. Stevenson, MD, MS	Norton Children's Hospital, Louisville, KY
Margaret Samuels-Kalow, MD, MPhil, Wayne G. Shreffler, MD, PhD, and Ari R. Cohen, MD	Massachusetts General Hospital, Boston, MA
Anne K. Beasley, MD and Cindy S. Bauer, MD	Phoenix Children's Hospital, Phoenix, AZ
Thida Ong, MD and Markus Boos, MD, PhD	Seattle Children's Hospital, Seattle, WA
Charles G. Macias, MD, MPH	Texas Children's Hospital, Houston, TX

Table S2. Comparisons between analytic and non-analytic cohorts in MARC-35

Characteristics	Analytic cohort (n=744; 73%)	Non-analytic cohort (n=272; 27%)	P value
**Demographics**			
Age (month), median (IQR)	3 (2-6)	3 (2-6)	0.38
Female sex	300 (40)	106 (39)	0.75
Race/ethnicity			<0.001
Non-Hispanic white	332 (45)	98 (36)	
Non-Hispanic black	183 (25)	56 (21)	
Hispanic	229 (31)	79 (29)	
Other or unknown	0 (0)	39 (14)	
Prematurity (32.0-36.9 weeks)	136 (18)	50 (18)	0.99
C-section delivery	250 (34)	98 (36)	0.58
Previous breathing problems (count)			0.56
0	587 (79)	223 (82)	
1	122 (16)	38 (14)	
2	35 (5)	11 (4)	
Previous ICU admission	12 (2)	5 (2)	0.99
History of eczema	111 (15)	38 (14)	0.78
Ever attended daycare	170 (23)	64 (24)	0.89
Parental history of asthma	246 (33)	99 (37)	0.35
Parental history of eczema	143 (19)	55 (20)	0.78
**Clinical presentation**			
Weight (kg), median (IQR)	6 (5-8)	6 (5-8)	0.22
Respiratory rate (per minute), median (IQR)	48 (40-60)	50 (40-60)	0.06
Oxygen saturation			0.68
<90%	62 (9)	29 (11)	
90-93%	113 (16)	42 (16)	
≥94%	553 (76)	196 (73)	
Blood eosinophilia (≥4%)	70 (11)	26 (11)	0.99
IgE sensitization	155 (21)	49 (18)	0.37
**Clinical course**			
Positive pressure ventilation use*	39 (5)	16 (6)	0.81
Intensive treatment use†	114 (15)	49 (18)	0.35
Length-of-day (day), median (IQR)	2 (1-3)	2 (1-3)	0.68
Corticosteroid use	92 (12)	40 (15)	0.38
**Respiratory virus**			
RSV only	421 (57)	165 (61)	0.28

RV only	52 (7)	8 (3)	0.02
RSV/RV coinfection	81 (11)	41 (15)	0.09
Other pathogen‡	179 (24)	58 (21)	0.41

Abbreviations: ICU, intensive care unit; IgE, immunoglobulin E; IQR, interquartile range; RSV, respiratory syncytial virus; RV, rhinovirus.

Data are no. (%) of infants unless otherwise indicated. Percentages may not equal 100 because of rounding and missingness.

* Infants with bronchiolitis who underwent continuous positive airway ventilation and/or mechanical ventilation.

† Infants with bronchiolitis who were admitted to ICU and/or who underwent positive pressure ventilation.

‡ Adenovirus, bocavirus, *Bordetella pertussis*, enterovirus, human coronavirus NL63, OC43, 229E, or HKU1, human metapneumovirus, influenza A or B virus, *Mycoplasma pneumoniae*, and parainfluenza virus 1-3.

Table S3. Baseline characteristics and clinical course of infants, according to childhood asthma development by age 6 years

Characteristics	Asthma development (n=164; 26%)	No asthma development (n=475; 74%)	P value
**Demographics**			
Age (month), median (IQR)	5 (2-6)	4 (2-6)	0.001
Female sex	57 (35)	202 (43)	0.098
Race/ethnicity			0.001
Non-Hispanic white	62 (38)	238 (50)	
Non-Hispanic black	56 (34)	94 (20)	
Hispanic	46 (28)	143 (30)	
Prematurity (32.0-36.9 weeks)	80 (17)	35 (21)	0.24
C-section delivery	61 (38)	149 (32)	
Previous breathing problems (count)			<0.001
0	108 (66)	403 (85)	
1	43 (26)	57 (12)	
2	13 (8)	15 (3)	
Previous ICU admission	3 (2)	7 (2)	0.99
History of eczema	41 (25)	52 (11)	<0.001
Ever attended daycare	39 (24)	108 (23)	0.87
Parental history of asthma	77 (47)	128 (27)	<0.001
Parental history of eczema	50 (31)	73 (15)	<0.001
**Clinical presentation**			
Weight (kg), median (IQR)	7 (5-8)	6 (5-8)	0.001
Respiratory rate (per minute), median (IQR)	51 (40-60)	49 (40-60)	0.043
Oxygen saturation			0.43
<90%	12 (8)	43 (9)	
90-93%	20 (13)	74 (16)	
≥94%	127 (80)	349 (75)	
Blood eosinophilia (≥4%)	15 (11)	44 (11)	0.99
IgE sensitization	46 (28)	88 (19)	0.013
**Clinical course**			
Positive pressure ventilation use*	10 (6)	25 (5)	0.84
Intensive treatment use†	25 (15)	70 (15)	0.98
Length-of-day (day), median (IQR)	3 (1-3)	3 (1-3)	0.96
Corticosteroid use	31 (19)	46 (10)	0.003
**Respiratory virus**			
RSV only	72 (44)	304 (64)	<0.001
RV only	22 (13)	21 (4)	<0.001
RSV/RV coinfection	19 (12)	51 (11)	0.88
Other pathogen‡	49 (30)	94 (20)	0.01

Abbreviations: ICU, intensive care unit; IgE, immunoglobulin E; IQR, interquartile range; RSV, respiratory syncytial virus; RV, rhinovirus.

Data are no. (%) of infants unless otherwise indicated. Percentages may not equal 100, because of rounding and missingness.

* Infants with bronchiolitis who underwent continuous positive airway ventilation and/or mechanical ventilation.

† Infants with bronchiolitis who were admitted to ICU and/or who underwent positive pressure ventilation.
‡ Adenovirus, bocavirus, Bordetella pertussis, enterovirus, human coronavirus NL63, OC43, 229E, or HKU1, human metapneumovirus, influenza A or B virus, Mycoplasma pneumoniae, and parainfluenza virus 1-3.

Table S4. Summary of the 900 SNPs associated with each of the candidate metabolites

Metabolite name	SNP	Chr	BP	Ref. allel	MAF	P-value	Beta	SE
**Non-Hispanic white sample**								
13-HODE + 9-HODE	rs1384643	11	18884376	C	0.209	5.41E-07	-0.445	0.087
	rs1384644	11	18885362	A	0.208	8.98E-07	-0.437	0.087
	rs1979489	11	18879541	A	0.215	6.80E-07	-0.440	0.087
	rs56146952	6	21487688	A	0.033	7.40E-07	0.981	0.194
	rs7112140	11	18886000	A	0.208	8.98E-07	-0.437	0.087
2-stearoyl-GPE (18:0)	rs111896185	2	32343684	A	0.005	5.93E-07	2.228	0.437
	rs112137751	2	32343842	A	0.005	5.93E-07	2.228	0.437
	rs112467416	8	124264806	A	0.005	1.25E-08	2.526	0.432
	rs112792411	2	32551527	T	0.005	5.93E-07	2.228	0.437
	rs112943681	2	32592266	G	0.005	5.93E-07	2.228	0.437
	rs113193228	2	32485161	T	0.005	5.93E-07	2.228	0.437
	rs113582564	2	32343406	A	0.005	5.93E-07	2.228	0.437
	rs117141467	8	124193221	A	0.027	3.90E-07	0.850	0.164
	rs117166109	16	74504681	T	0.032	1.15E-07	0.987	0.182
	rs117587778	8	124131546	C	0.027	3.90E-07	0.850	0.164
	rs118066943	16	74531782	A	0.032	1.15E-07	0.987	0.182
	rs12118747	1	68185261	T	0.066	5.51E-08	0.686	0.123
	rs12133637	1	68182171	A	0.066	5.51E-08	0.686	0.123
	rs12142889	1	68192586	C	0.068	9.22E-07	0.600	0.12
	rs12145412	1	68190016	G	0.071	2.64E-07	0.618	0.117
	rs12486303	3	70251324	T	0.03	5.45E-07	0.894	0.175
	rs12723245	1	68196136	T	0.068	9.22E-07	0.600	0.12
	rs12741237	1	68187137	T	0.071	7.11E-07	0.610	0.121
	rs138709795	4	115974344	T	0.021	6.48E-07	0.975	0.192
	rs141347512	16	74637004	T	0.03	2.08E-07	0.993	0.187
	rs141616663	7	69010200	G	0.003	2.85E-07	2.798	0.533
	rs144105348	16	74489429	A	0.032	1.15E-07	0.987	0.182
	rs144150283	5	63094042	A	0.018	7.14E-07	1.119	0.221
	rs147696464	4	54554080	A	0.015	5.12E-08	1.343	0.241
	rs148416765	16	74456834	C	0.032	1.15E-07	0.987	0.182
	rs16912839	9	108282566	T	0.003	9.97E-07	2.665	0.534
	rs189396899	5	63197934	T	0.021	8.52E-07	1.034	0.206
	rs35307984	1	68197816	A	0.071	2.64E-07	0.618	0.117
	rs542894513	2	32536368	G	0.005	5.93E-07	2.228	0.437
	rs562563198	2	32536369	A	0.005	5.93E-07	2.228	0.437

	rs576151215	2	32536371	A	0.005	5.93E-07	2.228	0.437
	rs62378780	5	123314348	C	0.02	8.48E-07	1.071	0.213
	rs7198425	16	86781553	C	0.006	8.88E-07	1.897	0.378
	rs72756725	5	62984752	C	0.018	7.14E-07	1.119	0.221
	rs72855966	2	32512657	C	0.005	5.93E-07	2.228	0.437
	rs72855982	2	32525174	C	0.005	5.93E-07	2.228	0.437
	rs72867217	2	32361573	G	0.005	5.93E-07	2.228	0.437
	rs72867248	2	32417611	G	0.005	5.93E-07	2.228	0.437
	rs72867249	2	32417730	C	0.005	5.93E-07	2.228	0.437
	rs72867267	2	32458238	T	0.005	5.93E-07	2.228	0.437
	rs76707409	3	70250566	T	0.03	5.45E-07	0.894	0.175
	rs78313593	2	32579888	G	0.005	5.93E-07	2.228	0.437
	rs78774212	3	70254524	C	0.03	5.45E-07	0.894	0.175
	rs78835427	1	68193650	A	0.068	9.22E-07	0.600	0.12
	rs78972438	2	32476618	T	0.005	5.93E-07	2.228	0.437
	rs79172049	2	32433873	A	0.005	5.93E-07	2.228	0.437
	rs79802815	8	29611345	T	0.017	8.43E-07	1.215	0.242
	rs79834450	2	32600504	C	0.005	5.93E-07	2.228	0.437
	rs80082678	8	124206017	G	0.027	3.90E-07	0.850	0.164
arabonate/xylonate	rs6506040	18	3007418	A	0.105	2.74E-07	-0.635	0.121
sphingomyelin (d17:1/16:0, d18:1/15:0, d16:1/17:0)	rs10906537	10	13903714	A	0.265	8.31E-08	0.453	0.082
	rs117541335	7	51330112	G	0.02	2.78E-07	1.406	0.268
	rs138929727	3	36867300	T	0.029	6.08E-07	1.175	0.231
	rs141454309	7	51320188	T	0.021	1.25E-07	1.395	0.258
	rs142997609	1	166499198	T	0.03	9.66E-09	1.274	0.216
	rs146020378	2	161888149	A	0.017	9.80E-07	1.463	0.293
	rs149833699	1	166630611	T	0.026	2.57E-08	1.336	0.234
	rs4776813	15	66654768	T	0.425	2.88E-07	0.422	0.08
arabitol	rs111637674	3	51676169	T	0.041	1.22E-07	0.742	0.137
	rs111685767	12	111738726	T	0.026	5.92E-07	0.892	0.175
	rs111807267	3	51341991	T	0.044	9.25E-07	0.667	0.133
	rs112060328	12	112366683	T	0.026	5.92E-07	0.892	0.175
	rs112502212	12	112008036	C	0.026	5.92E-07	0.892	0.175
	rs112953992	2	101585155	C	0.023	4.89E-07	0.866	0.169
	rs113361773	1	19827490	C	0.015	1.40E-08	1.273	0.219
	rs113536369	1	19827456	T	0.015	1.40E-08	1.273	0.219
	rs113710104	12	111588867	T	0.026	5.92E-07	0.892	0.175
	rs113812800	2	3901708	C	0.051	2.38E-07	0.656	0.124
	rs114004761	2	32670379	T	0.005	1.17E-07	2.138	0.394
	rs114093045	5	8350506	T	0.041	7.46E-09	0.772	0.13
	rs114278957	5	50657115	G	0.02	1.07E-07	0.972	0.179

rs114424030	4	87418455	T	0.02	6.42E-07	1.024	0.202
rs114592831	2	97791822	A	0.02	1.03E-07	1.050	0.193
rs115078050	1	19824596	T	0.015	1.40E-08	1.273	0.219
rs115097356	1	19824070	A	0.014	6.19E-07	1.185	0.233
rs115759315	1	19827378	T	0.015	1.40E-08	1.273	0.219
rs115777460	13	52988125	A	0.003	6.56E-10	3.028	0.475
rs115807631	8	11942323	A	0.003	1.99E-07	2.570	0.483
rs115977651	1	19827212	G	0.015	1.40E-08	1.273	0.219
rs116083656	8	11936679	C	0.003	1.99E-07	2.570	0.483
rs116138295	1	19832735	G	0.015	1.40E-08	1.273	0.219
rs116469118	2	32765976	A	0.005	1.17E-07	2.138	0.394
rs116575346	1	19828552	A	0.015	1.40E-08	1.273	0.219
rs116623810	5	45413703	A	0.021	4.42E-07	0.899	0.174
rs117086652	9	124602927	A	0.023	6.62E-07	0.916	0.181
rs117099074	18	11041900	T	0.02	6.18E-07	0.982	0.193
rs11786235	8	15760604	C	0.018	3.61E-07	1.089	0.21
rs1227556524	18	73606273	T	0.008	1.27E-07	1.672	0.309
rs12401938	1	19870736	C	0.023	9.74E-10	1.177	0.187
rs12403258	1	19908860	G	0.023	1.14E-11	1.307	0.185
rs12406639	1	160467623	A	0.003	1.42E-07	2.599	0.483
rs12469533	2	32604961	T	0.003	1.24E-07	2.611	0.483
rs12555626	9	38598373	T	0.038	2.53E-07	0.760	0.144
rs138274331	3	51732516	A	0.042	3.38E-07	0.706	0.135
rs138603619	17	78945892	T	0.018	7.93E-07	1.011	0.201
rs138617060	1	19827937	T	0.015	1.40E-08	1.273	0.219
rs139431743	1	19829814	A	0.015	1.40E-08	1.273	0.219
rs139703115	2	32587709	A	0.005	1.17E-07	2.138	0.394
rs140445141	16	58342404	A	0.024	3.85E-07	0.907	0.175
rs141539894	8	11931617	T	0.003	1.99E-07	2.570	0.483
rs141861849	5	50734969	G	0.02	1.07E-07	0.972	0.179
rs142444541	6	169050781	C	0.005	8.26E-07	1.994	0.397
rs142635845	5	45288903	A	0.021	4.42E-07	0.899	0.174
rs142807487	8	86517435	G	0.018	1.72E-08	1.149	0.199
rs143208057	1	19831046	T	0.015	1.40E-08	1.273	0.219
rs143270861	4	182064534	A	0.015	2.61E-08	1.238	0.217
rs143401161	8	11924998	C	0.003	1.99E-07	2.570	0.483
rs143881342	1	19829837	A	0.015	1.40E-08	1.273	0.219
rs144150283	5	63094042	A	0.018	6.77E-09	1.177	0.198
rs144188035	8	86471333	T	0.02	1.33E-07	1.039	0.193
rs144256089	2	32665651	G	0.005	1.17E-07	2.138	0.394
rs144874884	5	45616438	G	0.021	5.04E-07	0.894	0.174

rs146725162	9	124615316	A	0.023	6.62E-07	0.916	0.181
rs146986595	5	137006796	T	0.018	4.24E-07	1.043	0.202
rs147883964	2	32497930	G	0.005	1.17E-07	2.138	0.394
rs149111614	1	19830894	G	0.015	1.40E-08	1.273	0.219
rs149754947	4	21024602	A	0.029	8.09E-07	0.811	0.161
rs150550457	1	19827913	T	0.015	1.40E-08	1.273	0.219
rs150822141	3	51647972	T	0.041	1.22E-07	0.742	0.137
rs151089927	5	39162522	C	0.017	7.34E-07	1.056	0.209
rs1629996	5	166339322	A	0.039	6.73E-08	0.735	0.133
rs16823095	1	19908537	T	0.023	1.14E-11	1.307	0.185
rs182786224	2	32754869	T	0.005	1.17E-07	2.138	0.394
rs184036761	9	1933947	G	0.003	8.11E-08	2.638	0.48
rs185153229	6	34748997	C	0.039	7.02E-08	0.709	0.128
rs188436982	8	11922754	T	0.003	1.99E-07	2.570	0.483
rs188898054	1	19833629	C	0.015	1.40E-08	1.273	0.219
rs189396899	5	63197934	T	0.021	3.77E-07	0.966	0.186
rs190930717	1	19827935	C	0.015	1.40E-08	1.273	0.219
rs192005306	2	32321366	T	0.005	1.17E-07	2.138	0.394
rs192830962	2	32427179	T	0.005	1.17E-07	2.138	0.394
rs192871044	1	19898509	G	0.024	1.61E-10	1.194	0.181
rs2298110	1	19906558	G	0.023	1.14E-11	1.307	0.185
rs2315057	1	19854919	T	0.023	9.74E-10	1.177	0.187
rs2315058	1	19855908	C	0.023	9.74E-10	1.177	0.187
rs2315059	1	19885299	G	0.024	1.61E-10	1.194	0.181
rs2679227	3	102139808	G	0.015	1.30E-07	1.241	0.23
rs35864749	5	77411613	T	0.13	1.93E-07	0.431	0.081
rs4687770	3	51721049	C	0.131	4.35E-07	0.395	0.077
rs4687771	3	51721096	A	0.131	4.35E-07	0.395	0.077
rs4687775	3	51735190	T	0.13	1.99E-07	0.413	0.078
rs540292551	5	45241358	A	0.021	4.42E-07	0.899	0.174
rs546529454	12	111704148	T	0.026	5.92E-07	0.892	0.175
rs575200228	12	111957553	G	0.029	5.14E-07	0.851	0.166
rs57579391	9	134280592	C	0.074	6.75E-07	0.536	0.106
rs58412051	3	51731491	T	0.107	4.27E-07	0.445	0.086
rs6445949	3	51733958	G	0.145	4.12E-07	0.387	0.075
rs6445958	3	51738281	G	0.13	2.01E-07	0.413	0.078
rs6445959	3	51738331	G	0.13	2.01E-07	0.413	0.078
rs66524851	3	51722626	T	0.13	2.03E-07	0.412	0.078
rs6778196	3	51736413	T	0.107	4.47E-07	0.444	0.086
rs72727620	1	175744164	G	0.014	8.76E-08	1.254	0.229
rs72727621	1	175748947	G	0.014	8.76E-08	1.254	0.229

	rs72756725	5	62984752	C	0.018	6.77E-09	1.177	0.198
	rs736577	4	138443464	A	0.015	7.98E-07	1.116	0.222
	rs74105457	13	95195987	G	0.005	1.22E-07	2.142	0.396
	rs74428042	12	111618876	C	0.026	5.92E-07	0.892	0.175
	rs74882332	6	169002626	T	0.005	8.26E-07	1.994	0.397
	rs74952634	1	19864935	T	0.023	9.74E-10	1.177	0.187
	rs75302586	12	111999206	C	0.026	5.92E-07	0.892	0.175
	rs76203979	6	168985885	C	0.005	8.26E-07	1.994	0.397
	rs76379349	8	11888466	C	0.003	1.99E-07	2.570	0.483
	rs76753577	1	19860477	C	0.023	9.74E-10	1.177	0.187
	rs76821272	12	111571337	G	0.026	5.92E-07	0.892	0.175
	rs77060617	12	4630227	G	0.009	3.59E-07	1.473	0.283
	rs77070824	12	111492836	C	0.026	5.92E-07	0.892	0.175
	rs77244206	15	31337658	G	0.018	7.57E-07	1.090	0.216
	rs77838113	12	111527721	G	0.027	1.33E-07	0.913	0.169
	rs78278314	5	8380739	G	0.06	1.50E-09	0.667	0.107
	rs78852962	8	15790678	A	0.018	3.61E-07	1.089	0.21
	rs78908125	13	37460454	G	0.014	2.16E-07	1.097	0.207
	rs79258786	1	175745880	T	0.014	8.76E-08	1.254	0.229
	rs79408798	4	20951073	G	0.024	7.13E-07	0.885	0.175
	rs79413114	1	19890825	T	0.024	1.61E-10	1.194	0.181
	rs9839297	3	51737360	C	0.13	2.01E-07	0.413	0.078
	rs9839502	3	51737516	A	0.13	2.01E-07	0.413	0.078
	rs9839728	3	51737812	G	0.13	2.01E-07	0.413	0.078
	rs9876970	3	51737540	G	0.13	2.01E-07	0.413	0.078
guanosine	rs10857843	1	110998913	C	0.218	5.07E-07	0.435	0.085
	rs11072489	15	74497063	A	0.133	7.17E-07	0.505	0.1
	rs76439278	7	9104576	A	0.011	1.34E-07	1.742	0.323
alpha-tocopherol	rs10787475	10	113132709	T	0.363	7.16E-07	0.404	0.08
	rs142997609	1	166499198	T	0.03	3.47E-07	1.186	0.228
	rs1467576	10	113128969	G	0.381	7.96E-07	0.401	0.08
	rs6585209	10	113127429	G	0.377	5.33E-07	0.406	0.079
	rs7906315	10	113127459	A	0.377	5.33E-07	0.406	0.079
urate	rs10757215	9	21397346	C	0.166	7.47E-07	-0.502	0.099
	rs10757216	9	21397348	T	0.163	7.61E-07	-0.505	0.1
	rs10757217	9	21397826	A	0.166	7.47E-07	-0.502	0.099
	rs10757218	9	21398148	A	0.166	7.47E-07	-0.502	0.099
	rs10964979	9	21398025	C	0.163	7.61E-07	-0.505	0.1
	rs10964980	9	21404644	G	0.167	5.14E-07	-0.508	0.099
	rs11076512	16	50097943	C	0.218	5.91E-07	0.469	0.092
	rs11640627	16	50103990	G	0.218	5.91E-07	0.469	0.092

	rs11642579	16	50098169	C	0.218	5.91E-07	0.469	0.092
	rs12934889	16	50107946	A	0.221	8.42E-07	0.462	0.092
	rs1558813	16	50105888	T	0.218	5.91E-07	0.469	0.092
	rs2058813	16	50095543	T	0.218	5.91E-07	0.469	0.092
	rs2990141	9	21389579	G	0.104	9.36E-07	-0.593	0.119
	rs3904254	13	69729260	A	0.414	9.83E-07	-0.361	0.072
	rs4785381	16	50099878	C	0.218	5.91E-07	0.469	0.092
	rs4978113	9	21401107	C	0.166	4.26E-07	-0.514	0.1
	rs614647	9	21377613	A	0.262	2.39E-07	-0.445	0.084
	rs624704	9	21383899	G	0.268	8.58E-08	-0.461	0.084
	rs632941	9	21388713	A	0.267	1.54E-07	-0.452	0.084
	rs649053	9	21387419	G	0.24	3.01E-08	-0.488	0.086
	rs8047421	16	50095354	G	0.218	5.91E-07	0.469	0.092
	rs8052350	16	50105374	G	0.218	5.91E-07	0.469	0.092
	rs8062151	16	50096601	T	0.218	5.91E-07	0.469	0.092
	rs9542062	13	69731604	G	0.414	9.83E-07	-0.361	0.072
N-acetylneuraminate	rs10101380	8	5147537	C	0.249	9.05E-07	0.424	0.085
	rs13240649	7	3217341	A	0.405	8.70E-07	0.407	0.081
	rs1377884	8	5148069	A	0.249	9.05E-07	0.424	0.085
	rs2463076	19	24285479	T	0.057	6.93E-07	-0.826	0.163
1,2-dioleoyl-GPG (18:1/18:1)	rs10483989	14	88138044	G	0.014	2.07E-07	1.356	0.255
	rs111438609	1	21945696	A	0.005	3.21E-07	2.181	0.418
	rs112489638	1	21948188	C	0.005	3.21E-07	2.181	0.418
	rs113043905	19	8717141	G	0.059	5.95E-07	0.593	0.116
	rs113612090	2	149966077	A	0.012	3.66E-07	1.330	0.256
	rs113898699	1	21946421	T	0.005	3.21E-07	2.181	0.418
	rs116459436	1	7227920	T	0.014	4.96E-07	1.276	0.249
	rs117541335	7	51330112	G	0.02	4.80E-07	1.042	0.203
	rs11805717	1	244020635	A	0.032	4.81E-07	0.831	0.162
	rs11829496	12	56676978	T	0.02	1.99E-07	1.143	0.215
	rs11831054	12	56630330	C	0.02	1.99E-07	1.143	0.215
	rs11838315	12	56676863	A	0.02	1.99E-07	1.143	0.215
	rs142277549	14	88122695	T	0.014	2.07E-07	1.356	0.255
	rs142746752	7	97787347	A	0.005	3.64E-07	2.169	0.417
	rs142997609	1	166499198	T	0.03	4.38E-07	0.853	0.165
	rs143289895	15	51641243	G	0.017	6.48E-07	1.121	0.221
	rs144882212	15	51677020	T	0.017	6.48E-07	1.121	0.221
	rs146533672	10	124551875	T	0.009	2.83E-07	1.647	0.314
	rs146635511	10	124772492	T	0.009	2.77E-09	1.894	0.31
	rs146900373	12	56669262	A	0.02	1.99E-07	1.143	0.215
	rs147298806	3	126306723	T	0.027	6.37E-08	0.959	0.173

rs148239997	1	21944098	A	0.005	3.21E-07	2.181	0.418
rs149274803	12	56651405	T	0.02	1.99E-07	1.143	0.215
rs1494445	7	146691851	A	0.012	3.39E-07	1.444	0.277
rs1494451	7	146698699	G	0.014	1.36E-07	1.445	0.268
rs150042936	12	56659486	A	0.02	1.99E-07	1.143	0.215
rs150871583	15	51492980	A	0.018	2.86E-07	1.105	0.211
rs1587045	7	146696349	A	0.014	1.36E-07	1.445	0.268
rs1587046	7	146695626	T	0.014	1.36E-07	1.445	0.268
rs1637750	7	2188176	G	0.44	9.35E-07	0.284	0.057
rs16826053	1	21929191	C	0.005	3.21E-07	2.181	0.418
rs16826058	1	21933257	A	0.005	3.21E-07	2.181	0.418
rs17118901	12	56668854	A	0.02	1.99E-07	1.143	0.215
rs1800638	12	56639542	A	0.02	1.99E-07	1.143	0.215
rs184332240	1	21937484	A	0.005	3.21E-07	2.181	0.418
rs2533101	7	146698198	A	0.014	1.36E-07	1.445	0.268
rs2533104	7	146695911	T	0.014	1.36E-07	1.445	0.268
rs3935288	1	178565682	T	0.127	1.90E-08	0.462	0.08
rs4262757	12	56682054	T	0.02	1.99E-07	1.143	0.215
rs4329480	1	21979700	T	0.005	3.21E-07	2.181	0.418
rs4394606	1	21953322	T	0.005	3.21E-07	2.181	0.418
rs4585955	1	21963542	A	0.005	3.21E-07	2.181	0.418
rs4654774	1	21945146	T	0.005	3.21E-07	2.181	0.418
rs4654775	1	21958644	G	0.005	3.21E-07	2.181	0.418
rs4654776	1	21959967	T	0.005	3.21E-07	2.181	0.418
rs4988924	1	21975262	A	0.005	3.21E-07	2.181	0.418
rs55736705	1	178557304	G	0.107	2.63E-08	0.498	0.087
rs56311661	6	78642444	T	0.017	2.56E-07	1.213	0.23
rs56411229	1	178568582	G	0.127	1.90E-08	0.462	0.08
rs566235379	15	51506615	A	0.018	2.86E-07	1.105	0.211
rs57406430	12	56673712	A	0.02	1.99E-07	1.143	0.215
rs57881068	16	85523695	A	0.009	3.46E-07	1.535	0.295
rs60312497	1	21959317	G	0.005	3.21E-07	2.181	0.418
rs60574031	12	56680661	T	0.02	1.99E-07	1.143	0.215
rs6660523	1	21968440	G	0.005	3.21E-07	2.181	0.418
rs6980130	7	146700933	G	0.033	1.98E-07	0.925	0.174
rs7090719	10	125270520	T	0.009	6.55E-07	1.642	0.323
rs7136370	12	56658703	C	0.02	1.99E-07	1.143	0.215
rs72707006	1	178299092	A	0.101	3.66E-07	0.475	0.091
rs7307573	12	56681620	A	0.02	1.99E-07	1.143	0.215
rs74091637	12	56646323	A	0.02	1.99E-07	1.143	0.215
rs7527325	1	21970933	T	0.005	3.21E-07	2.181	0.418

	rs7531732	1	21949203	A	0.005	3.21E-07	2.181	0.418
	rs7554644	1	21948957	G	0.005	3.21E-07	2.181	0.418
	rs76632518	3	188632276	G	0.048	2.64E-07	0.701	0.133
	rs77795265	1	21949265	T	0.005	3.21E-07	2.181	0.418
	rs78860243	12	56671403	A	0.02	1.99E-07	1.143	0.215
	rs79277901	12	56657509	T	0.02	1.99E-07	1.143	0.215
	rs79429192	1	21947410	G	0.005	3.21E-07	2.181	0.418
	rs7954160	12	56654352	T	0.02	1.99E-07	1.143	0.215
	rs7956952	12	56637306	C	0.018	7.59E-07	1.124	0.223
	rs7958515	12	56655681	T	0.02	1.99E-07	1.143	0.215
	rs7977632	12	56659979	G	0.02	1.99E-07	1.143	0.215
	rs9738534	12	56647074	T	0.02	1.99E-07	1.143	0.215
taurochenodeoxycholate	rs10120987	9	83220251	T	0.407	6.83E-08	0.386	0.07
	rs10867955	9	83232010	G	0.419	1.90E-07	0.367	0.069
	rs112306900	9	83227670	G	0.419	1.90E-07	0.367	0.069
	rs11794740	9	83221402	T	0.42	2.68E-07	0.361	0.069
	rs12237222	9	83219828	G	0.428	6.03E-07	-0.366	0.072
	rs1408103	9	83219702	A	0.426	6.84E-07	-0.366	0.072
	rs148476836	9	83227074	A	0.419	1.90E-07	0.367	0.069
	rs28772597	9	83228556	A	0.42	2.68E-07	0.361	0.069
	rs28829626	9	83228393	T	0.42	2.68E-07	0.361	0.069
	rs28840703	9	83223016	T	0.419	1.72E-07	0.368	0.069
	rs28890897	9	83227878	C	0.42	2.68E-07	0.361	0.069
	rs3003432	1	17272654	G	0.413	1.07E-07	0.385	0.071
	rs34051324	1	17305315	G	0.389	3.93E-07	0.374	0.072
	rs6586517	1	17303702	T	0.389	6.57E-07	0.365	0.072
	rs6657142	1	17298429	T	0.386	5.15E-07	0.368	0.072
	rs7046601	9	83229547	C	0.419	1.90E-07	0.367	0.069
	rs73606754	19	54420809	G	0.045	4.87E-07	0.857	0.167
	rs7469571	9	83227721	T	0.42	2.68E-07	0.361	0.069
	rs7548900	1	17296159	A	0.387	3.32E-07	0.374	0.072
	rs77227240	9	83227806	A	0.417	2.60E-07	0.362	0.069
	rs7863072	9	83230069	C	0.429	3.57E-07	0.357	0.069
carnosine	rs12075516	1	240025649	A	0.14	7.82E-07	-0.541	0.107
	rs12096705	1	240043527	T	0.143	6.10E-07	-0.543	0.107
	rs12742786	1	240031658	A	0.143	6.10E-07	-0.543	0.107
	rs1489955	4	177293157	T	0.035	5.90E-07	-0.993	0.195
	rs34752899	1	240031695	G	0.143	6.10E-07	-0.543	0.107
	rs34798713	1	240021314	A	0.143	9.40E-08	-0.580	0.106
	rs35177311	1	240045199	C	0.143	6.10E-07	-0.543	0.107
	rs35659437	2	149221140	G	0.029	9.68E-07	1.114	0.223

	rs61828734	1	240060963	A	0.172	5.45E-07	-0.498	0.097
	rs6429176	1	240062083	A	0.414	2.50E-07	-0.398	0.075
	rs71646803	1	240021795	T	0.143	9.40E-08	-0.580	0.106
	rs71646810	1	240037949	T	0.143	6.10E-07	-0.543	0.107
	rs71646811	1	240058726	A	0.172	8.36E-07	-0.496	0.099
glutarate (C5-DC)	rs10099268	8	133330350	G	0.098	2.92E-07	0.649	0.124
	rs1311081086	14	23920363	G	0.014	7.93E-07	1.624	0.322
	rs2232187	1	147759651	A	0.011	6.48E-07	1.904	0.375
	rs4631442	8	133328812	G	0.098	2.92E-07	0.649	0.124
	rs74744988	14	23904826	C	0.014	7.93E-07	1.624	0.322
	rs76134657	14	23880000	T	0.014	7.93E-07	1.624	0.322
	rs78575271	5	22221275	A	0.018	7.90E-07	1.545	0.307
mevalonolactone	rs115354992	5	153802891	C	0.054	8.74E-07	-0.812	0.162
	rs117176367	11	1702693	T	0.083	3.64E-07	0.722	0.139
	rs13427590	2	197652097	T	0.479	6.32E-07	-0.386	0.076
	rs142115089	5	153852236	A	0.054	8.74E-07	-0.812	0.162
	rs17115211	5	153776697	T	0.054	8.74E-07	-0.812	0.162
	rs17356099	5	153763488	C	0.054	8.74E-07	-0.812	0.162
	rs4752744	11	1697036	G	0.081	2.71E-07	0.740	0.141
	rs76082460	11	1703201	A	0.083	3.64E-07	0.722	0.139
	rs77745430	5	153829655	A	0.054	8.74E-07	-0.812	0.162
	rs79456580	11	1702888	G	0.083	3.64E-07	0.722	0.139
malate	rs111269517	8	102494130	A	0.02	6.66E-07	-1.443	0.284
	rs145755236	8	102496732	C	0.02	6.66E-07	-1.443	0.284
	rs2987533	13	48986378	A	0.137	3.93E-07	-0.598	0.115
	rs9806917	16	23379535	T	0.009	2.64E-07	-2.247	0.427
palmitoylcarnitine (C16)	rs10787475	10	113132709	T	0.363	8.32E-07	0.379	0.075
	rs142997609	1	166499198	T	0.03	4.33E-07	1.111	0.215
	rs1467576	10	113128969	G	0.381	9.41E-07	0.376	0.075
	rs149833699	1	166630611	T	0.026	9.69E-07	1.163	0.233
	rs1960491	21	27296936	A	0.181	6.84E-07	-0.485	0.096
	rs2019758	21	27287894	T	0.176	2.52E-07	-0.503	0.095
	rs2830836	21	27291519	G	0.173	7.39E-07	-0.486	0.096
	rs2830837	21	27291666	A	0.173	7.39E-07	-0.486	0.096
	rs2830839	21	27292824	T	0.178	7.71E-07	-0.483	0.096
	rs2830842	21	27297861	C	0.178	2.76E-07	-0.501	0.095
	rs6585209	10	113127429	G	0.377	3.12E-07	0.391	0.075
	rs7906315	10	113127459	A	0.377	3.12E-07	0.391	0.075
	rs8132126	21	27305533	G	0.187	6.46E-07	-0.472	0.093
	rs9982998	21	27295123	G	0.175	3.69E-07	-0.495	0.095
nicotinate	rs17692499	2	211291536	A	0.018	9.20E-07	1.397	0.279

dimethylglycine	rs10915298	1	4952241	A	0.045	9.92E-07	0.892	0.179
	rs17732220	10	115992822	C	0.041	1.34E-07	-0.954	0.177
	rs75465543	1	4953501	A	0.045	9.92E-07	0.892	0.179
	rs78856887	1	4953497	A	0.045	9.92E-07	0.892	0.179
N-acetylaspartate (NAA)	rs28675771	4	670119	A	0.345	2.76E-07	0.455	0.087
docosapentaenoate (DPA; 22:5n3)	rs10512800	3	131144157	C	0.009	5.19E-08	1.672	0.3
	rs10806776	6	163620438	A	0.024	1.32E-08	1.111	0.19
	rs10945911	6	163626478	T	0.024	1.32E-08	1.111	0.19
	rs112068670	1	60249374	C	0.018	4.68E-07	1.095	0.213
	rs112467416	8	124264806	A	0.005	1.03E-07	2.278	0.418
	rs112478987	8	89722922	A	0.003	9.03E-07	2.578	0.515
	rs113667790	4	122486051	G	0.009	5.56E-07	1.525	0.298
	rs115052250	5	180813754	T	0.047	1.23E-08	0.792	0.135
	rs11579040	1	239009594	T	0.053	7.85E-07	0.636	0.126
	rs117588399	19	2685617	T	0.012	1.93E-07	1.226	0.23
	rs12202535	6	163618921	A	0.026	9.20E-08	1.014	0.185
	rs12203381	6	163627518	G	0.024	1.32E-08	1.111	0.19
	rs12524087	6	163619816	G	0.024	1.32E-08	1.111	0.19
	rs12618447	2	15131598	T	0.011	5.11E-07	1.427	0.278
	rs13156573	5	29530917	G	0.104	8.71E-07	0.481	0.096
	rs13176764	5	29568581	T	0.121	4.75E-07	0.472	0.092
	rs137949601	4	81851732	G	0.006	7.72E-07	1.840	0.365
	rs138219310	4	177916559	C	0.015	5.09E-07	1.260	0.246
	rs141616663	7	69010200	G	0.003	5.78E-07	2.623	0.514
	rs142002493	19	2683786	C	0.012	1.93E-07	1.226	0.23
	rs143843271	20	55369802	A	0.05	8.55E-07	0.664	0.132
	rs144150283	5	63094042	A	0.018	6.07E-10	1.330	0.208
	rs144267578	11	67290254	G	0.021	6.54E-08	1.087	0.196
	rs145001636	11	67014533	G	0.023	6.69E-07	1.006	0.198
	rs146934712	11	67225007	G	0.02	5.99E-07	1.043	0.205
	rs150656348	12	64394726	T	0.035	5.64E-09	0.944	0.158
	rs182130953	4	177971540	G	0.015	5.09E-07	1.260	0.246
	rs189396899	5	63197934	T	0.021	8.25E-08	1.079	0.197
	rs45533837	11	119359048	C	0.02	1.87E-07	1.092	0.205
	rs571920132	8	89713232	G	0.003	9.03E-07	2.578	0.515
	rs58603854	2	15125184	T	0.011	5.11E-07	1.427	0.278
	rs62090744	18	32980924	G	0.024	2.37E-07	0.982	0.186
	rs6800326	3	130569097	A	0.035	5.84E-07	0.834	0.163
	rs6901862	6	163615109	A	0.029	2.13E-07	0.948	0.179
	rs7198425	16	86781553	C	0.006	1.99E-07	1.926	0.362
	rs72754650	5	62858480	C	0.026	6.26E-07	0.917	0.18

	rs72754657	5	62862174	G	0.024	1.40E-07	0.995	0.185
	rs72756725	5	62984752	C	0.018	6.07E-10	1.330	0.208
	rs72758773	5	63217690	T	0.02	9.29E-08	1.113	0.204
	rs72769140	5	62502275	T	0.021	6.14E-07	1.007	0.198
	rs73784498	6	163616586	T	0.027	1.48E-07	0.971	0.181
	rs74608789	8	89707449	T	0.003	9.03E-07	2.578	0.515
	rs74727314	1	60306871	A	0.018	4.68E-07	1.095	0.213
	rs74906027	4	177926616	T	0.015	5.09E-07	1.260	0.246
	rs75360525	8	89768395	G	0.003	9.03E-07	2.578	0.515
	rs75448494	4	177969859	A	0.015	5.09E-07	1.260	0.246
	rs7580440	2	29754943	T	0.009	5.75E-07	1.683	0.33
	rs75872265	8	89776370	T	0.003	9.03E-07	2.578	0.515
	rs75926607	8	89762169	T	0.003	9.03E-07	2.578	0.515
	rs76625733	5	180816549	A	0.045	7.29E-09	0.815	0.137
	rs77989946	8	89697155	C	0.003	9.03E-07	2.578	0.515
	rs78388829	2	15120216	G	0.011	5.11E-07	1.427	0.278
	rs78522856	8	89745312	A	0.003	9.03E-07	2.578	0.515
	rs78673538	4	177932848	A	0.015	5.09E-07	1.260	0.246
	rs78929526	8	89763815	G	0.003	9.03E-07	2.578	0.515
	rs78948244	12	64470880	C	0.032	6.01E-09	0.986	0.165
	rs79917991	5	180814057	T	0.047	1.23E-08	0.792	0.135
	rs9346966	6	163617407	T	0.029	2.13E-07	0.948	0.179
	rs9355432	6	163612617	C	0.027	1.48E-07	0.971	0.181
	rs9356127	6	163617542	C	0.03	9.98E-07	0.873	0.175
	rs9456871	6	163617269	C	0.029	2.13E-07	0.948	0.179
	rs9456872	6	163617422	T	0.029	2.13E-07	0.948	0.179
octanoylcarnitine (C8)	rs73243695	4	24246161	C	0.105	2.97E-08	0.678	0.119
	rs9385987	6	142105107	T	0.256	1.73E-07	0.477	0.089
N-acetylarginine	rs117045903	15	66627247	A	0.062	2.19E-08	-0.903	0.157
	rs1938060	6	65835763	T	0.205	9.02E-07	0.450	0.09
	rs4452626	6	65836128	C	0.205	9.02E-07	0.450	0.09
	rs4493733	6	65826644	G	0.205	9.02E-07	0.450	0.09
	rs4901529	14	54685333	A	0.078	7.85E-07	0.701	0.139
	rs8028010	15	94184482	A	0.03	9.14E-07	-1.153	0.23
	rs9351522	6	65834393	T	0.205	9.02E-07	0.450	0.09
	rs9363451	6	65822620	A	0.28	2.93E-07	0.433	0.083
	rs9453487	6	65906182	A	0.232	6.40E-07	0.451	0.089
erythronate	rs1013347	13	49236745	T	0.256	3.29E-07	-0.503	0.096
	rs12184794	13	49224602	G	0.268	4.72E-07	-0.492	0.096
	rs12871418	13	49241565	C	0.261	3.96E-07	-0.496	0.096
	rs6561506	13	49223896	T	0.265	5.31E-07	-0.490	0.096

	rs6561507	13	49229212	C	0.268	4.72E-07	-0.492	0.096
	rs7296992	12	100671461	G	0.486	8.79E-07	0.412	0.082
	rs7338304	13	49231354	T	0.268	4.72E-07	-0.492	0.096
	rs8002911	13	49224255	C	0.27	6.27E-07	-0.484	0.095
	rs9526531	13	49239474	A	0.259	1.18E-07	-0.516	0.095
	rs9526532	13	49247108	C	0.256	3.29E-07	-0.503	0.096
	rs9535170	13	49241269	A	0.262	7.85E-07	-0.480	0.095
	rs9535172	13	49246203	C	0.256	3.29E-07	-0.503	0.096
	rs9568169	13	49220514	A	0.268	4.72E-07	-0.492	0.096
	rs9568171	13	49227368	C	0.268	4.72E-07	-0.492	0.096
	rs9568172	13	49238662	C	0.262	2.78E-07	-0.498	0.095
	rs9568173	13	49241875	T	0.256	3.29E-07	-0.503	0.096
	rs9596073	13	49223424	G	0.268	4.72E-07	-0.492	0.096
aconitate [cis or trans]	rs148027659	10	74609570	G	0.018	5.80E-07	1.359	0.266
1-linoleoyl-GPE (18:2)	rs10089838	8	22448539	C	0.083	7.87E-07	0.571	0.113
	rs10108972	8	22448578	A	0.083	7.87E-07	0.571	0.113
	rs11120217	1	213805016	G	0.024	9.64E-08	1.230	0.225
	rs11235012	11	87206512	T	0.036	2.77E-07	0.916	0.174
	rs112827618	1	213805434	A	0.024	9.64E-08	1.230	0.225
	rs11800931	1	213798304	G	0.023	7.09E-09	1.382	0.232
	rs11805961	1	213804041	T	0.024	9.64E-08	1.230	0.225
	rs11989798	8	22413139	A	0.072	9.40E-07	0.607	0.121
	rs11990553	8	22413284	T	0.072	9.40E-07	0.607	0.121
	rs11992035	8	22443610	G	0.083	7.87E-07	0.571	0.113
	rs12023908	1	213797058	G	0.023	7.09E-09	1.382	0.232
	rs12025802	1	213804606	T	0.024	9.64E-08	1.230	0.225
	rs12027606	1	213811682	G	0.024	9.64E-08	1.230	0.225
	rs12027781	1	213806264	G	0.024	9.64E-08	1.230	0.225
	rs12028443	1	213812081	G	0.024	9.64E-08	1.230	0.225
	rs12028562	1	213806380	C	0.024	9.64E-08	1.230	0.225
	rs12029128	1	213804699	C	0.024	9.64E-08	1.230	0.225
	rs12029144	1	213798766	A	0.023	7.09E-09	1.382	0.232
	rs12029630	1	213807608	C	0.024	9.64E-08	1.230	0.225
	rs12061908	1	213796934	A	0.023	7.09E-09	1.382	0.232
	rs12546391	8	22437715	C	0.081	3.59E-07	0.603	0.116
	rs138048035	4	9140678	A	0.009	9.48E-07	1.673	0.335
	rs139001058	4	9374848	C	0.009	9.48E-07	1.673	0.335
	rs139168984	4	9379728	T	0.009	9.48E-07	1.673	0.335
	rs141131810	4	9138391	C	0.009	9.48E-07	1.673	0.335
	rs144513322	8	22404243	G	0.053	2.86E-08	0.827	0.145
	rs147529784	4	9141061	C	0.009	9.48E-07	1.673	0.335

	rs147696464	4	54554080	A	0.015	4.48E-07	1.341	0.26
	rs1522248	8	22442411	A	0.083	7.87E-07	0.571	0.113
	rs1996811	8	22443283	G	0.083	7.87E-07	0.571	0.113
	rs2068643	1	213800700	A	0.023	7.09E-09	1.382	0.232
	rs2149502	1	14478988	T	0.044	1.00E-06	0.789	0.158
	rs2272080	8	22441213	G	0.081	3.59E-07	0.603	0.116
	rs28654839	8	22441708	A	0.083	4.15E-07	0.594	0.115
	rs35860879	8	22438258	A	0.081	3.59E-07	0.603	0.116
	rs56840890	8	22401902	A	0.054	2.85E-08	0.823	0.145
	rs56939496	8	22401932	A	0.057	4.85E-09	0.846	0.141
	rs57009280	8	22446278	C	0.083	7.87E-07	0.571	0.113
	rs73544036	8	22401462	G	0.057	4.85E-09	0.846	0.141
	rs73670409	8	22369256	G	0.074	7.12E-07	0.627	0.124
	rs73670410	8	22374182	A	0.074	7.12E-07	0.627	0.124
	rs75324395	8	22430545	T	0.041	3.23E-07	0.857	0.164
	rs76038954	1	213796838	G	0.023	7.09E-09	1.382	0.232
	rs7821470	8	22447988	C	0.083	7.87E-07	0.571	0.113
	rs7831649	8	22440981	A	0.081	3.59E-07	0.603	0.116
	rs7839390	8	22409300	T	0.054	2.85E-08	0.823	0.145
	rs7843761	8	22448286	C	0.083	7.87E-07	0.571	0.113
	rs870215	8	22400624	A	0.059	5.73E-09	0.830	0.139
	rs9785086	8	22444449	T	0.083	7.87E-07	0.571	0.113
	rs9785088	8	22444760	G	0.083	7.87E-07	0.571	0.113
	rs9785089	8	22444800	G	0.083	7.87E-07	0.571	0.113
	rs9886589	8	22441326	G	0.081	3.59E-07	0.603	0.116
	rs9886594	8	22440959	G	0.081	3.59E-07	0.603	0.116
glycerophosphoinositol	rs1009909	7	132988761	A	0.441	8.18E-07	0.377	0.075
	rs10264205	7	133006865	C	0.417	1.97E-07	0.395	0.074
	rs10267851	7	133006774	A	0.408	6.12E-08	0.418	0.075
	rs10269489	7	132987569	A	0.432	4.16E-07	0.391	0.076
	rs1035294	7	132999627	C	0.432	4.16E-07	0.391	0.076
	rs11014515	10	18499351	G	0.036	7.17E-07	1.013	0.2
	rs12707063	7	132972113	C	0.432	4.16E-07	0.391	0.076
	rs13290694	9	130169549	T	0.054	7.84E-07	0.836	0.166
	rs2342810	7	132784249	A	0.476	7.97E-07	-0.364	0.072
	rs6508149	18	24103708	T	0.006	3.17E-07	2.452	0.469
	rs73404299	18	24105673	G	0.006	3.17E-07	2.452	0.469
	rs7809527	7	132998287	T	0.441	6.01E-07	0.381	0.075
**Non-Hispanic black sample**								
13-HODE + 9-	rs11576652	1	226098419	C	0.112	8.09E-07	0.844	0.165

HODE	rs1438301	5	12931189	T	0.484	7.65E-08	-0.51	0.091
	rs34930211	5	12932414	C	0.407	1.31E-07	0.521	0.095
	rs73658708	9	116883635	T	0.038	2.08E-08	1.441	0.245
2-stearoyl-GPE (18:0)	rs112534057	15	60266023	T	0.038	6.59E-07	1.186	0.23
	rs113420860	15	78959141	C	0.107	1.27E-07	0.752	0.136
	rs12210578	6	23982014	A	0.071	9.67E-07	0.913	0.18
	rs12494581	3	55257122	C	0.298	7.25E-07	-0.5	0.098
	rs183022724	3	88153092	A	0.022	4.43E-07	1.631	0.311
	rs2002832	15	78950397	T	0.134	7.73E-07	0.665	0.13
	rs2032022	1	48922950	T	0.454	7.40E-07	-0.43	0.084
	rs3121522	1	48894201	T	0.374	8.46E-07	-0.45	0.088
	rs3127565	1	48900036	C	0.377	5.27E-07	-0.46	0.088
	rs36072781	6	23972206	A	0.071	9.67E-07	0.913	0.18
	rs404915	3	55254986	A	0.314	9.72E-07	-0.5	0.098
	rs55916950	4	181855793	T	0.046	5.09E-07	1.096	0.21
	rs73419214	15	60265218	T	0.038	6.59E-07	1.186	0.23
	rs8192673	2	164497202	C	0.309	3.97E-09	-0.57	0.091
arabonate/xylonate	rs114848830	5	25375408	A	0.033	7.71E-07	1.49	0.29
	rs12123801	1	166592258	G	0.128	8.04E-07	-0.85	0.166
	rs35514775	1	183573732	G	0.066	7.36E-07	1.207	0.235
	rs546847929	5	25368397	G	0.033	7.71E-07	1.49	0.29
	rs549351270	5	25380160	G	0.033	7.71E-07	1.49	0.29
	rs58973984	3	60129706	C	0.123	3.52E-07	0.818	0.154
	rs72882789	3	60129587	A	0.191	4.81E-07	0.705	0.135
sphingomyelin (d17:1/16:0, d18:1/15:0, d16:1/17:0)	rs11153989	6	121017082	T	0.374	1.19E-07	-0.59	0.106
	rs12732801	1	203053519	T	0.24	7.40E-07	0.596	0.116
	rs12752641	1	203053298	C	0.246	5.04E-07	0.595	0.114
	rs13047185	21	34126118	C	0.019	7.04E-07	1.872	0.363
	rs2291691	1	203053994	C	0.249	6.90E-07	0.588	0.114
	rs61111848	1	203054943	C	0.24	7.40E-07	0.596	0.116
	rs77499805	13	98753079	A	0.156	4.09E-07	-0.74	0.141
	rs77666895	1	234584691	A	0.06	7.99E-07	1.014	0.198
	rs78340409	1	234553708	G	0.044	1.32E-07	1.272	0.231
	rs8107764	19	7722032	A	0.475	6.54E-07	-0.54	0.105
arabitol	rs111672193	10	34060697	A	0.033	6.12E-07	1.105	0.213
	rs12436940	14	31741232	G	0.426	9.79E-07	-0.38	0.075
	rs12886260	14	31751914	C	0.426	9.79E-07	-0.38	0.075
	rs1400275	3	145351415	A	0.074	3.68E-07	-0.77	0.145
	rs2216946	2	217271551	C	0.137	4.66E-07	-0.61	0.117
	rs344971	3	145360822	A	0.074	3.68E-07	-0.77	0.145
	rs344977	3	145416227	T	0.074	3.68E-07	-0.77	0.145

	rs344995	3	145424869	G	0.074	3.68E-07	-0.77	0.145
	rs345029	3	145406164	G	0.074	3.68E-07	-0.77	0.145
	rs345058	3	145388183	G	0.074	3.68E-07	-0.77	0.145
	rs7494358	14	31757034	C	0.426	9.79E-07	-0.38	0.075
	rs76921328	10	34065978	C	0.033	6.12E-07	1.105	0.213
guanosine	rs11132851	4	170418046	T	0.071	4.70E-07	0.917	0.175
	rs112308097	2	230821995	T	0.041	8.67E-08	1.326	0.237
	rs1406516	4	170414249	G	0.074	7.24E-07	0.889	0.173
	rs1534656	4	170416250	A	0.071	4.70E-07	0.917	0.175
	rs1609379	4	170420010	G	0.071	4.70E-07	0.917	0.175
	rs74527054	2	173412629	T	0.126	4.14E-07	0.741	0.141
	rs76536316	2	173411957	T	0.123	7.30E-07	0.731	0.142
alpha-tocopherol	rs12942941	17	80129214	A	0.361	8.98E-07	0.522	0.102
	rs2361718	17	80129667	T	0.328	9.08E-07	0.535	0.105
	rs4471729	17	49350803	T	0.085	3.67E-07	0.929	0.176
	rs8066452	17	80130281	G	0.361	8.98E-07	0.522	0.102
	rs8077849	17	80130338	T	0.361	8.98E-07	0.522	0.102
	rs8081698	17	80130413	A	0.361	8.98E-07	0.522	0.102
urate	rs112362780	17	37723113	A	0.06	2.58E-07	-1.06	0.197
	rs12002545	9	101282604	A	0.011	7.43E-07	2.234	0.435
	rs12492339	3	86262622	A	0.287	7.89E-07	-0.54	0.105
	rs13065946	3	86268251	T	0.287	7.89E-07	-0.54	0.105
	rs13092675	3	86260212	A	0.287	7.89E-07	-0.54	0.105
	rs1512919	3	86270519	G	0.29	6.83E-07	-0.54	0.105
	rs1512920	3	86274343	C	0.29	6.83E-07	-0.54	0.105
	rs1816709	2	153327843	C	0.2	5.22E-07	-0.6	0.115
	rs2351752	5	154588462	T	0.131	9.86E-08	-0.79	0.142
	rs35961500	3	86273813	C	0.29	6.83E-07	-0.54	0.105
	rs6763450	3	86257501	G	0.287	7.89E-07	-0.54	0.105
	rs6774344	3	86257109	C	0.287	7.89E-07	-0.54	0.105
	rs6788794	3	86254303	T	0.287	7.89E-07	-0.54	0.105
	rs6791998	3	86260961	A	0.287	7.89E-07	-0.54	0.105
	rs6793146	3	86255938	A	0.287	7.89E-07	-0.54	0.105
	rs7319837	13	24657152	T	0.221	9.00E-07	0.566	0.111
	rs7338915	13	24657497	A	0.221	9.00E-07	0.566	0.111
	rs77780840	14	63422807	C	0.016	9.19E-07	-1.88	0.369
	rs77817750	3	66424342	G	0.019	8.27E-07	1.769	0.346
	rs78232398	3	66418844	C	0.019	8.27E-07	1.769	0.346
	rs79282949	3	66418398	A	0.019	8.27E-07	1.769	0.346
	rs9511396	13	24657064	A	0.224	6.38E-07	0.572	0.111
N-acetylneuraminate	rs116246100	13	53865305	C	0.098	4.95E-07	-0.91	0.174

	rs148846446	3	65997183	C	0.019	4.66E-07	-1.96	0.374
	rs17047802	2	56325870	C	0.235	4.70E-07	-0.63	0.121
	rs2077681	6	3085866	T	0.342	7.04E-07	-0.58	0.113
	rs28661076	7	19504200	G	0.2	7.29E-07	0.672	0.131
	rs4795778	17	33403050	T	0.063	4.38E-07	-0.96	0.182
	rs59257726	13	53868921	C	0.098	4.95E-07	-0.91	0.174
	rs6597167	6	5817232	G	0.21	9.48E-07	-0.61	0.119
	rs76840346	13	53885705	C	0.101	3.50E-07	-0.91	0.172
	rs77504374	13	53886153	G	0.101	3.50E-07	-0.91	0.172
	rs9391981	6	3086772	G	0.342	7.04E-07	-0.58	0.113
	rs9405191	6	3086719	G	0.342	7.04E-07	-0.58	0.113
	rs9914451	17	33403363	C	0.063	4.38E-07	-0.96	0.182
1,2-dioleoyl-GPG (18:1/18:1)	rs10110025	8	14516874	G	0.495	5.70E-07	-0.49	0.094
	rs12058444	1	101650249	C	0.257	6.78E-07	-0.57	0.11
	rs12090003	1	101672044	T	0.246	7.73E-07	-0.58	0.112
	rs13374704	1	101648076	A	0.268	8.14E-07	-0.56	0.11
	rs145099041	1	101658118	G	0.24	3.79E-07	-0.59	0.112
	rs146232470	4	167659452	G	0.019	9.20E-07	1.713	0.336
	rs2453331	5	38042699	A	0.23	1.60E-08	0.619	0.104
	rs28403869	1	101667559	T	0.246	7.73E-07	-0.58	0.112
	rs55657103	1	101675298	G	0.246	7.73E-07	-0.58	0.112
	rs61534578	1	101674230	G	0.246	7.73E-07	-0.58	0.112
	rs72984795	1	101671248	A	0.246	7.73E-07	-0.58	0.112
	rs72984796	1	101671584	T	0.246	7.73E-07	-0.58	0.112
	rs7550042	1	101665207	C	0.246	7.73E-07	-0.58	0.112
	rs7555247	1	101648743	T	0.268	8.14E-07	-0.56	0.11
	rs76968348	4	91057405	A	0.019	8.73E-07	1.48	0.29
taurochenodeoxycholate	rs58248404	6	33997991	A	0.128	2.16E-07	0.706	0.131
	rs6477421	9	993546	G	0.358	1.43E-07	0.513	0.094
	rs72816230	17	1219933	G	0.052	6.15E-07	1.061	0.205
	rs76403141	6	33997776	C	0.128	2.16E-07	0.706	0.131
carnosine	rs112362780	17	37723113	A	0.06	4.15E-07	-1	0.19
	rs11679159	2	217763071	T	0.24	5.20E-07	-0.54	0.104
	rs59692785	2	217757789	G	0.137	4.80E-07	-0.64	0.122
phosphocholine	rs10649	1	201134866	A	0.074	4.59E-08	-1.21	0.212
	rs116023248	17	4569482	C	0.011	2.44E-07	-2.69	0.5
	rs11633493	15	59543398	G	0.016	4.39E-08	-2.57	0.448
	rs34206618	15	59551359	A	0.016	4.39E-08	-2.57	0.448
	rs4609784	15	59543966	T	0.016	4.39E-08	-2.57	0.448
	rs62066370	17	4568942	G	0.011	2.44E-07	-2.69	0.5
	rs62066371	17	4572224	C	0.011	2.44E-07	-2.69	0.5

	rs62066372	17	4572305	T	0.011	2.44E-07	-2.69	0.5
	rs6696361	1	201137187	A	0.074	4.59E-08	-1.21	0.212
	rs73081051	1	201138326	T	0.071	1.35E-07	-1.19	0.217
	rs73960527	18	56138709	A	0.068	2.77E-07	-1.17	0.218
	rs76048308	17	4574158	C	0.011	2.44E-07	-2.69	0.5
	rs78250794	17	4568774	T	0.011	2.44E-07	-2.69	0.5
glutarate (C5-DC)	rs113655051	10	43236475	A	0.118	6.76E-07	-0.69	0.134
	rs12651261	4	154049599	G	0.145	1.73E-07	0.722	0.133
	rs1845656	5	97278401	C	0.213	9.66E-07	0.591	0.116
	rs7524634	1	27313372	T	0.06	3.40E-07	0.984	0.185
	rs8073779	17	4773788	A	0.189	9.40E-08	-0.65	0.117
mevalonolactone	rs61894457	11	97870174	G	0.022	9.65E-08	-1.8	0.324
	rs7031683	9	7594949	A	0.09	1.78E-07	0.869	0.16
	rs7811174	7	17425006	C	0.492	9.13E-07	-0.5	0.098
malate	rs10824481	10	76987458	C	0.306	9.42E-07	0.475	0.093
	rs3824716	10	76991052	A	0.303	8.17E-07	0.476	0.093
palmitoylcarnitine (C16)	rs10427249	2	105990117	T	0.068	8.10E-07	0.993	0.194
	rs77666895	1	234584691	A	0.06	4.81E-07	1.009	0.193
	rs78340409	1	234553708	G	0.044	8.19E-08	1.262	0.225
	rs79141561	2	105989161	G	0.068	8.10E-07	0.993	0.194
	rs9484844	6	144102979	G	0.15	6.78E-07	0.749	0.145
	rs9534251	13	45926160	T	0.159	3.25E-07	-0.77	0.145
nicotinate	rs114540933	3	33816896	A	0.03	9.38E-07	1.434	0.282
	rs114696228	3	33824840	A	0.033	6.80E-07	1.395	0.27
	rs1194648	10	52410437	G	0.238	9.72E-07	0.616	0.121
	rs1205603	10	52405013	G	0.227	8.07E-07	0.618	0.121
	rs17855475	1	180178877	C	0.109	8.06E-07	0.745	0.145
	rs62242629	3	8735624	A	0.096	5.88E-07	-0.86	0.165
	rs74450908	3	33865245	C	0.033	6.80E-07	1.395	0.27
dimethylglycine	rs12186076	3	41793147	T	0.459	8.94E-07	0.491	0.096
	rs16948404	17	13746314	A	0.112	5.54E-07	0.776	0.149
	rs4466711	10	67601733	T	0.298	2.21E-07	0.574	0.106
	rs8052562	16	3491830	T	0.126	7.21E-07	0.798	0.155
N-acetylaspartate (NAA)	rs114109678	3	27609130	C	0.186	7.01E-07	0.571	0.111
	rs115590964	3	16806848	C	0.049	4.81E-07	1.062	0.203
	rs11714340	3	27610676	C	0.156	5.01E-07	0.661	0.127
	rs12635190	3	27615001	T	0.156	5.01E-07	0.661	0.127
	rs13317121	3	27610219	T	0.186	7.01E-07	0.571	0.111
	rs13317270	3	27610461	A	0.186	7.01E-07	0.571	0.111
	rs13320787	3	27610144	C	0.186	7.01E-07	0.571	0.111
	rs13324107	3	27610142	C	0.186	7.01E-07	0.571	0.111

	rs13324211	3	27610471	C	0.186	7.01E-07	0.571	0.111
	rs28607843	3	16805586	T	0.049	4.81E-07	1.062	0.203
	rs55672224	3	27609623	G	0.186	7.01E-07	0.571	0.111
	rs55937808	3	27609560	A	0.186	7.01E-07	0.571	0.111
	rs6783349	3	27609235	G	0.186	7.01E-07	0.571	0.111
	rs73824214	3	27618890	A	0.159	7.10E-07	0.653	0.127
	rs74341935	3	16723792	T	0.063	3.66E-07	0.864	0.163
	rs7636988	3	27612360	T	0.156	5.01E-07	0.661	0.127
	rs78310529	17	53196962	A	0.107	6.24E-07	0.734	0.142
	rs9824374	3	16766078	T	0.052	7.26E-07	1.04	0.202
	rs9824467	3	16757105	A	0.052	7.26E-07	1.04	0.202
	rs9844053	3	16766083	C	0.052	7.26E-07	1.04	0.202
3-methyl-2-oxobutyrate	rs10966219	9	24109010	C	0.011	6.02E-07	-2.41	0.465
	rs115652568	6	12441062	C	0.011	8.01E-07	-2.33	0.455
	rs13121902	4	171167531	A	0.052	8.88E-07	1.176	0.231
	rs28775870	3	102740527	A	0.036	4.75E-07	-1.33	0.254
	rs34045937	4	171154743	T	0.049	6.83E-07	1.225	0.237
	rs66943105	4	171200734	G	0.052	8.88E-07	1.176	0.231
	rs67407149	4	171205399	T	0.049	6.83E-07	1.225	0.237
	rs73863972	3	102683264	C	0.033	7.70E-07	-1.35	0.263
	rs7612648	3	102728965	G	0.036	4.75E-07	-1.33	0.254
	rs76625879	11	124076797	C	0.019	1.60E-07	-2	0.366
	rs872070	4	171168423	C	0.052	8.88E-07	1.176	0.231
	rs9496833	6	144003395	T	0.082	4.30E-07	0.942	0.179
	rs9496835	6	144004166	A	0.093	1.83E-07	0.859	0.158
	rs9496838	6	144018802	G	0.101	3.32E-08	0.884	0.153
	rs9496841	6	144023140	G	0.098	8.25E-08	0.866	0.155
	rs9496844	6	144034976	G	0.104	1.17E-07	0.844	0.153
	rs9496847	6	144058415	A	0.09	4.65E-07	0.918	0.175
	rs9496850	6	144069372	G	0.079	7.61E-08	1.026	0.183
	rs9822912	3	102739949	T	0.036	4.75E-07	-1.33	0.254
	rs9825362	3	102692907	A	0.036	4.75E-07	-1.33	0.254
	rs9961696	18	28289208	T	0.402	9.59E-07	-0.49	0.096
docosapentaenoate (DPA; 22:5n3)	rs10399747	1	33021334	T	0.046	7.80E-08	1.184	0.211
	rs10914648	1	33030696	T	0.046	7.80E-08	1.184	0.211
	rs10914658	1	33065149	A	0.049	4.52E-07	1.091	0.208
	rs12082671	1	33018104	C	0.049	4.82E-07	1.083	0.207
	rs12095142	1	33031776	C	0.046	7.80E-08	1.184	0.211
	rs12541487	8	88985505	A	0.038	3.88E-07	1.306	0.247
	rs150733842	2	226684477	A	0.036	3.84E-07	1.202	0.228
	rs17102245	1	78787933	T	0.022	7.52E-07	1.509	0.294

	rs17102251	1	78788471	G	0.022	7.52E-07	1.509	0.294
	rs17543749	8	31870692	A	0.057	5.23E-07	1.015	0.195
	rs28621571	10	43716054	T	0.041	7.29E-09	1.376	0.226
	rs59931175	15	46028036	A	0.153	5.97E-07	0.583	0.112
	rs72943450	1	78770792	A	0.022	7.52E-07	1.509	0.294
	rs73501411	16	8766907	A	0.161	4.17E-07	0.611	0.116
	rs73578505	8	31863824	C	0.057	5.23E-07	1.015	0.195
	rs73578508	8	31864205	G	0.057	5.23E-07	1.015	0.195
	rs73578512	8	31864592	A	0.057	5.23E-07	1.015	0.195
	rs73578520	8	31871276	G	0.057	5.23E-07	1.015	0.195
	rs868952478	10	50076899	T	0.022	5.21E-07	1.771	0.34
octanoylcarnitine (C8)	rs113717140	6	144091996	T	0.079	4.32E-07	0.962	0.183
	rs113887828	5	37959362	A	0.06	6.01E-07	-1.08	0.207
	rs114124202	6	144093151	C	0.079	4.32E-07	0.962	0.183
	rs115552151	14	72671043	C	0.063	1.18E-07	1.05	0.19
	rs116409163	14	72665457	C	0.066	7.39E-08	1.051	0.187
	rs1741325	20	4181543	G	0.246	2.92E-07	-0.58	0.108
	rs55920039	3	42047516	T	0.101	5.98E-07	0.816	0.157
	rs58819299	14	72664447	T	0.087	5.50E-09	0.981	0.16
	rs6650552	15	101086762	G	0.074	6.81E-07	0.938	0.182
	rs73075268	3	42048707	A	0.101	5.98E-07	0.816	0.157
	rs9484835	6	143974948	A	0.087	6.83E-07	0.885	0.172
	rs9496850	6	144069372	G	0.079	7.31E-07	0.936	0.182
	rs9496853	6	144078903	G	0.079	4.32E-07	0.962	0.183
	rs9496855	6	144086200	A	0.079	4.32E-07	0.962	0.183
	rs975580	3	42049196	T	0.101	5.98E-07	0.816	0.157
N-acetylarginine	rs16914296	10	59870871	G	0.336	7.36E-07	0.596	0.116
	rs199593963	19	52710930	T	0.087	8.62E-07	-0.98	0.191
	rs61333185	10	59865672	C	0.21	5.26E-07	0.709	0.136
	rs67498166	10	59868216	G	0.336	6.87E-07	0.585	0.113
erythronate	rs11194146	10	108525183	C	0.052	8.82E-07	0.939	0.184
	rs62079267	18	5614610	C	0.208	8.16E-07	-0.63	0.123
aconitate [cis or trans]	rs1173589	1	53707271	A	0.281	1.46E-07	0.602	0.11
	rs1173590	1	53707428	T	0.281	1.46E-07	0.602	0.11
	rs12632521	3	32682166	A	0.161	1.30E-07	0.774	0.14
	rs1780391	1	53712035	G	0.366	9.61E-07	0.542	0.107
	rs1780392	1	53709629	T	0.366	9.61E-07	0.542	0.107
	rs2374666	12	107134951	G	0.429	8.39E-08	-0.54	0.096
	rs4595640	12	107133235	A	0.404	5.69E-09	-0.59	0.096
	rs8013826	14	25784166	T	0.15	3.96E-07	-0.75	0.141
1-linoleoyl-GPE	rs10922987	1	88016091	A	0.213	6.94E-07	0.565	0.11

(18:2)	rs12402427	1	87809464	T	0.057	5.95E-07	0.998	0.192
	rs9600435	13	75138763	A	0.301	4.10E-07	0.526	0.1
glycerophosphoinositol	rs11932086	4	97272985	T	0.391	9.20E-07	-0.57	0.112
	rs11942165	4	97273225	T	0.391	9.20E-07	-0.57	0.112
	rs13044395	20	4634333	G	0.046	7.63E-07	-1.21	0.236
	rs17026596	4	97276332	A	0.391	9.20E-07	-0.57	0.112
	rs2123467	5	114966890	G	0.082	4.70E-07	-1.07	0.204
	rs56126228	4	97234743	G	0.191	3.56E-07	-0.72	0.137
	rs72809658	5	114989394	A	0.2	1.64E-07	-0.68	0.124
	rs9356791	6	22018352	A	0.424	4.49E-07	-0.55	0.105
	rs9366421	6	22017809	C	0.424	4.49E-07	-0.55	0.105
	rs9715234	4	97271772	T	0.391	9.20E-07	-0.57	0.112
**Hispanic samples**								
13-HODE + 9-HODE	rs2036184	4	28344153	T	0.304	7.28E-07	-0.52	0.101
	rs367705986	6	17015501	A	0.02	4.41E-07	1.626	0.312
	rs79412944	11	131282274	C	0.028	8.46E-07	1.337	0.264
2-stearoyl-GPE (18:0)	rs111228844	5	172870793	G	0.02	3.13E-07	1.61	0.305
	rs111815842	7	35499860	T	0.031	4.01E-07	1.245	0.238
	rs112072443	7	35506710	A	0.031	4.01E-07	1.245	0.238
	rs143011682	8	125819539	G	0.05	7.00E-07	0.968	0.189
	rs1550702	8	125793575	T	0.05	7.00E-07	0.968	0.189
	rs553908847	11	36144085	T	0.004	8.91E-07	-3.1	0.612
	rs59571924	4	76815268	G	0.085	1.02E-07	0.841	0.153
	rs77822914	7	35502746	A	0.031	4.01E-07	1.245	0.238
	rs77940216	8	125844003	G	0.05	7.00E-07	0.968	0.189
arabonate/xylonate	rs10917161	1	22133715	C	0.026	2.44E-07	-1.57	0.294
	rs12038516	1	22144389	C	0.026	2.44E-07	-1.57	0.294
	rs2235528	1	22127147	T	0.026	2.44E-07	-1.57	0.294
	rs4682083	3	112089263	C	0.127	5.79E-07	-0.72	0.139
sphingomyelin (d17:1/16:0, d18:1/15:0, d16:1/17:0)	rs112808745	9	31003287	T	0.09	3.30E-07	-0.81	0.153
	rs13289892	9	30995409	A	0.092	4.63E-07	-0.79	0.153
	rs34305344	9	31007281	G	0.094	1.94E-08	-0.88	0.15
	rs55784498	2	99577270	T	0.017	9.78E-07	1.871	0.371
	rs6822497	4	28789794	T	0.183	5.27E-07	-0.59	0.114
	rs77501054	9	30999473	C	0.09	3.30E-07	-0.81	0.153
arabitol	rs114185525	16	64662105	G	0.011	6.75E-07	2.001	0.391
	rs114678531	2	234139032	A	0.009	3.28E-08	2.452	0.428
	rs116344631	2	234141982	A	0.009	3.28E-08	2.452	0.428
	rs116526786	16	64660924	C	0.011	6.75E-07	2.001	0.391
	rs117607952	14	62757867	A	0.017	9.19E-07	1.568	0.31

	rs12987450	2	17163692	C	0.251	7.76E-07	-0.5	0.097
	rs181892639	2	234140513	A	0.009	3.28E-08	2.452	0.428
	rs287307	2	17169974	C	0.251	7.76E-07	-0.5	0.097
	rs2946773	2	17173320	A	0.245	8.05E-07	-0.5	0.099
	rs34051137	2	17165113	C	0.251	7.76E-07	-0.5	0.097
	rs6741107	2	38425430	A	0.114	8.45E-07	0.742	0.146
	rs75000784	16	64678668	G	0.011	6.75E-07	2.001	0.391
	rs77141377	2	234140176	A	0.009	3.28E-08	2.452	0.428
	rs77933572	16	64642752	G	0.011	6.75E-07	2.001	0.391
	rs78031444	2	234141058	C	0.009	3.28E-08	2.452	0.428
	rs78700985	16	64684147	C	0.011	6.75E-07	2.001	0.391
	rs79035655	2	234156873	C	0.009	3.28E-08	2.452	0.428
	rs79607794	12	128583749	T	0.011	7.43E-07	2.042	0.401
	rs79972035	2	234156579	T	0.009	3.28E-08	2.452	0.428
guanosine	rs494367	1	14209320	T	0.05	8.59E-07	1.055	0.208
alpha-tocopherol	rs187868016	1	57010337	G	0.085	8.15E-07	-0.85	0.167
	rs3131643	6	31475005	A	0.079	6.85E-07	-0.85	0.166
	rs56085832	18	2823279	A	0.203	6.57E-07	0.607	0.118
	rs7047158	9	126060097	A	0.391	5.41E-07	-0.44	0.085
urate	rs73106062	5	73294746	C	0.085	5.82E-07	-0.91	0.176
N-acetylneuraminate	rs4680275	3	151252370	A	0.199	5.65E-07	0.564	0.109
	rs56300419	12	99418551	C	0.083	4.87E-07	0.879	0.169
	rs78648300	12	44718595	A	0.035	9.49E-07	-1.25	0.248
	rs79076460	12	44719090	C	0.035	9.49E-07	-1.25	0.248
1,2-dioleoyl-GPG (18:1/18:1)	rs72952414	18	66454088	G	0.024	5.33E-07	1.47	0.284
	rs77707953	3	77252161	T	0.02	7.14E-07	1.58	0.309
taurochenodeoxycholate	rs7813257	8	133987675	T	0.074	8.78E-07	0.847	0.167
carnosine	rs151119306	11	31775873	A	0.015	4.57E-07	1.748	0.336
	rs2402353	7	118385709	A	0.496	3.03E-07	0.41	0.078
	rs6132364	20	2168749	C	0.452	9.47E-07	0.423	0.084
	rs9602501	13	84551177	G	0.153	8.82E-07	0.583	0.115
phosphocholine	rs73330786	8	121950682	A	0.1	8.97E-07	0.94	0.186
	rs73330788	8	121950686	T	0.1	8.97E-07	0.94	0.186
glutarate (C5-DC)	rs2123356	10	4786104	T	0.463	9.91E-07	-0.44	0.087
mevalonolactone	rs13331682	16	81186989	A	0.181	6.47E-08	0.654	0.117
malate	rs1450613	5	166474697	G	0.041	1.92E-07	1.245	0.231
	rs2065390	13	103720618	T	0.111	2.77E-07	0.864	0.163
	rs2065391	13	103720841	C	0.299	4.41E-07	0.569	0.109
	rs61970044	13	103711532	T	0.107	2.73E-07	0.893	0.168
	rs6822497	4	28789794	T	0.183	2.94E-07	-0.67	0.126
	rs7339054	13	103139610	C	0.238	8.40E-07	-0.6	0.119

	rs74773533	5	166474379	A	0.035	3.17E-07	1.314	0.249
	rs74935686	13	103711753	G	0.107	2.73E-07	0.893	0.168
palmitoylcarnitine (C16)	rs10915818	1	225388827	T	0.201	9.33E-07	-0.6	0.118
	rs12073468	1	225378844	T	0.218	3.21E-07	-0.61	0.115
	rs12081971	1	225384562	A	0.201	9.33E-07	-0.6	0.118
	rs6656932	1	225386008	T	0.201	9.33E-07	-0.6	0.118
	rs6663101	1	225384419	G	0.201	9.33E-07	-0.6	0.118
	rs8034787	15	72856004	T	0.107	1.13E-07	-0.76	0.139
nicotinate	rs10959592	9	11185626	T	0.022	8.42E-07	1.267	0.25
	rs112252987	22	31888427	C	0.017	4.05E-07	1.608	0.308
	rs12380473	9	11133835	C	0.022	4.05E-07	1.296	0.248
	rs148743352	1	247428724	A	0.011	1.03E-07	-2.13	0.386
	rs151244608	1	247428963	A	0.011	1.03E-07	-2.13	0.386
	rs6679474	1	4271686	A	0.031	1.83E-07	1.405	0.261
	rs71314757	22	21002830	A	0.044	4.67E-07	1.056	0.203
	rs78257259	9	7064185	A	0.017	5.16E-07	1.587	0.307
	rs78362268	1	4271955	C	0.031	1.83E-07	1.405	0.261
dimethylglycine	rs1611121	9	133642514	T	0.059	2.62E-07	-0.97	0.183
N-acetylaspartate (NAA)	rs10925401	1	237393830	T	0.406	5.12E-07	0.525	0.101
	rs111231541	1	165291947	G	0.024	7.71E-07	-1.42	0.28
	rs13058923	3	118880181	T	0.371	5.63E-07	0.529	0.102
	rs16842265	1	165300718	T	0.024	4.34E-07	-1.58	0.303
	rs56121373	1	165297771	T	0.022	5.39E-07	-1.63	0.316
	rs74118550	1	165299582	T	0.022	5.39E-07	-1.63	0.316
	rs991097	4	155268801	T	0.439	9.68E-08	0.529	0.096
docosapentaenoate (DPA; 22:5n3)	rs115081888	17	49949473	A	0.022	9.10E-08	-1.39	0.252
	rs149793817	17	49947386	T	0.02	4.91E-08	-1.48	0.262
	rs290660	15	95190280	G	0.179	6.91E-07	0.576	0.113
octanoylcarnitine (C8)	rs12520495	5	5802458	G	0.262	6.26E-07	-0.51	0.1
	rs62426461	6	78517273	G	0.203	8.61E-07	-0.53	0.105
	rs6822497	4	28789794	T	0.183	2.84E-07	-0.61	0.115
N-acetylarginine	rs115091720	8	9682860	T	0.013	4.16E-07	2.239	0.429
	rs183427130	11	76105594	A	0.057	2.79E-07	1.106	0.209
	rs6592619	11	76003867	G	0.066	1.84E-07	1.117	0.207
	rs7107438	11	75998789	C	0.066	1.84E-07	1.117	0.207
	rs7108353	11	76105193	T	0.059	2.79E-07	1.106	0.209
	rs7114160	11	75957154	A	0.055	2.42E-07	1.122	0.21
	rs73493952	11	75943132	G	0.05	9.62E-08	1.206	0.219
	rs73496123	11	76005393	T	0.068	1.84E-07	1.117	0.207
	rs76296602	11	76109925	C	0.059	2.79E-07	1.106	0.209
	rs77435082	11	75941421	T	0.039	6.67E-07	1.199	0.234

	rs77624317	5	165118661	G	0.013	4.53E-07	2.075	0.399
	rs77779262	18	23248067	A	0.026	1.12E-08	1.7	0.286
	rs78071389	11	75941419	A	0.039	6.67E-07	1.199	0.234
	rs7941207	11	75942212	G	0.037	1.65E-07	1.289	0.238
erythronate	rs4358579	5	145745270	G	0.164	8.17E-07	-0.66	0.129
	rs7686496	4	112420432	A	0.111	9.30E-07	-0.77	0.153
	rs848503	2	36430521	G	0.365	8.11E-07	-0.44	0.087
1-linoleoyl-GPE (18:2)	rs73259353	12	5478963	T	0.057	9.55E-08	0.993	0.18

Abbreviations: BP, base pair; Chr, chromosome; SNP, single nucleotide polymorphisms

Table S5. Summary of the 281 genetic loci associated with the candidate metabolites in non-Hispanic white and non-Hispanic black samples

Metabolite		Locus										
**Metabolite class**	**Metabolite name**	**Index SNP***	**N†**	**Chr**	**Index SNP* position**	**Alt allele**	**Overlap‡**	**P value**		**PP.H4§**		**Known asthma genes within index SNP* ± 500KB**
								**white**	**black**	**UKB white**	**CAPPA black**	
**Significant in Non-Hispanic white sample**												
Lipid	1,2-dioleoyl-GPG (18:1/18:1)	rs116459436	1	1	7227920	T		4.96E-07	0.009	0.513	0.267	
		rs16826053	37	1	21929191	C		3.21E-07	0.515	0.204	0.251	
		rs142997609	2	1	166499198	T	T	4.38E-07	0.983	0.189	0.244	
		rs3935288	48	1	178565682	T		1.90E-08	0.294	0.148	0.283	
		rs11805717	3	1	244020635	A		4.81E-07	0.545	0.185	0.257	*C1orf100*
		rs113612090	2	2	149966077	A		3.66E-07	NA	0.220	0.251	
		rs147298806	2	3	126306723	T		6.37E-08	0.303	0.185	0.246	
		rs76632518	1	3	188632276	G		2.64E-07	0.649	0.392	0.211	*LPP,FLJ42393, LPP-AS1,LPP-AS2,MIR28*
		rs56311661	1	6	78642444	T		2.56E-07	0.380	0.139	0.201	
		rs1637750	16	7	2188176	G		9.35E-07	0.740	0.656	0.259	*AMZ1*
		rs117541335	26	7	51330112	G		4.80E-07	0.780	0.197	0.268	
		rs142746752	51	7	97787347	A		3.64E-07	0.759	0.235	0.204	
		rs1587046	29	7	146695626	T		1.36E-07	0.720	0.184	0.292	
		rs146635511	2	10	124772492	T		2.77E-09	NA	0.651	0.235	*LHPP*
		rs7090719	30	10	125270520	T		6.55E-07	0.995	0.242	0.252	

		rs11831054	59	12	56630330	C		1.99E-07	0.661	0.180	0.406	*STAT6,NAB2,LRP1,GPR182,MYO1A,RDH16,SDR9C7,TAC3,TMEM194A,ZBTB39,HSD17B6,NACA,PRIM1,PTGES3,ESYT1,MYL6,MYL6B,RNF41,SMARCC2*
		rs142277549	3	14	88122695	T		2.07E-07	0.109	0.691	0.695	
		rs150871583	8	15	51492980	A		2.86E-07	0.117	0.218	0.185	*SCG3*
		rs57881068	2	16	85523695	A		3.46E-07	0.377	0.251	0.309	
		rs113043905	1	19	8717141	G		5.95E-07	0.453	0.580	0.251	*ADAMTS10,ACTL9,MUC16,OR1M1*
Lipid	13-HODE + 9-HODE	rs56146952	17	6	21487688	A	T	7.40E-07	0.507	0.328	0.282	*CASC15*
		rs1384643	37	11	18884376	C		5.41E-07	0.739	0.102	0.374	*NAV2*
Lipid	1-linoleoyl-GPE (18:2)	rs2149502	1	1	14478988	T		1.00E-06	0.771	0.244	0.242	
		rs76038954	74	1	213796838	G		7.09E-09	0.573	0.268	0.200	
		rs141131810	40	4	9138391	C		9.48E-07	0.520	0.312	0.252	
		rs147696464	1	4	54554080	A		4.48E-07	0.668	0.216	0.234	*PDGFRA*
		rs73544036	98	8	22401462	G		4.85E-09	0.822	0.222	0.235	
		rs11235012	3	11	87206512	T		2.77E-07	0.294	0.294	0.354	
Lipid	2-stearoyl-GPE (18:0)	rs12133637	15	1	68182171	A		5.51E-08	0.355	0.184	0.275	
		rs113582564	20	2	32343406	A		5.93E-07	0.497	0.185	0.235	
		rs76707409	9	3	70250566	T		5.45E-07	0.611	0.287	0.324	
		rs147696464	1	4	54554080	A		5.12E-08	0.251	0.154	0.260	*PDGFRA*
		rs138709795	3	4	115974344	T		6.48E-07	0.332	0.231	0.241	
		rs72756725	11	5	62984752	C		7.14E-07	0.286	0.169	0.234	
		rs62378780	4	5	123314348	C		8.48E-07	0.103	0.154	0.258	*CSNK1G3*
		rs141616663	64	7	69010200	G		2.85E-07	0.603	0.229	0.223	

		rs79802815	3	8	29611345	T		8.43E-07	0.479	0.384	0.298	
		rs117587778	6	8	124131546	C		3.90E-07	0.011	0.217	0.240	*TATDN1*
		rs112467416	2	8	124264806	A		1.25E-08	0.012	0.185	0.364	*TATDN1*
		rs16912839	25	9	108282566	T		9.97E-07	0.901	0.256	0.247	
		rs148416765	17	16	74456834	C		1.15E-07	0.910	0.160	0.231	
		rs7198425	3	16	86781553	C		8.88E-07	0.568	0.155	0.238	
Lipid	docosapentaenoate (DPA; 22:5n3)	rs112068670	8	1	60249374	C		4.68E-07	NA	0.154	0.240	
		rs11579040	10	1	239009594	T		7.85E-07	0.680	0.159	0.232	
		rs78388829	5	2	15120216	G		5.11E-07	0.784	0.695	0.278	*FAM84A,DDX1*
		rs7580440	13	2	29754943	T		5.75E-07	0.185	0.215	0.280	*LBH*
		rs6800326	8	3	130569097	A		5.84E-07	0.222	0.171	0.266	
		rs10512800	5	3	131144157	C		5.19E-08	0.400	0.268	0.357	
		rs137949601	1	4	81851732	G		7.72E-07	0.837	0.404	0.473	
		rs113667790	1	4	122486051	G		5.56E-07	0.842	0.149	0.178	*ADAD1,IL2,IL21,KIAA1109,IL21-AS1,BBS12*
		rs138219310	30	4	177916559	C		5.09E-07	0.302	0.154	0.277	
		rs13176764	21	5	29568581	T		4.75E-07	0.378	0.348	0.230	
		rs72756725	10	5	62984752	C		6.07E-10	0.526	0.172	0.275	
		rs76625733	19	5	180816549	A		7.29E-09	0.687	0.203	0.345	
		rs12524087	12	6	163619816	G		1.32E-08	0.574	0.841	0.287	*QKI*
		rs141616663	43	7	69010200	G		5.78E-07	0.472	0.415	0.332	
		rs77989946	43	8	89697155	C	T	9.03E-07	0.121	0.168	0.310	
		rs112467416	1	8	124264806	A		1.03E-07	0.452	0.165	0.233	*TATDN1*
		rs144267578	5	11	67290254	G		6.54E-08		0.258	0.241	
		rs45533837	3	11	119359048	C		1.87E-07	0.320	0.163	0.224	*CXCR5,BCL9L,MIR4492,UPK2,FOXR1,HYOU1*
		rs150656348	5	12	64394726	T		5.64E-09	0.531	0.139	0.340	
		rs7198425	1	16	86781553	C		1.99E-07	0.528	0.239	0.244	
		rs62090744	4	18	32980924	G		2.37E-07	NA	0.368	0.253	

		rs142002493	6	19	2683786	C		1.93E-07	0.571	0.156	0.205	*GNA15,LOC100996351,GNA11,S1PR4,AES*
		rs143843271	2	20	55369802	A		8.55E-07	0.501	0.127	0.226	
Lipid	glutarate (C5-DC: glutarylcarnitine)	rs2232187	3	1	147759651	A		6.48E-07	0.656	0.768	0.277	
		rs78575271	57	5	22221275	A		7.90E-07	0.919	0.201	0.231	
		rs4631442	30	8	133328812	G		2.92E-07	0.428	0.227	0.239	*TG*
		rs76134657	12	14	23880000	T		7.93E-07	0.790	0.187	0.260	
Lipid	glycerophosphoinositol	rs2342810	4	7	132784249	A		7.97E-07	0.071	0.171	0.410	
		rs10267851	174	7	133006774	A		6.12E-08	0.780	0.099	0.297	
		rs13290694	19	9	130169549	T		7.84E-07	0.602	0.099	0.311	*PTGES*
		rs11014515	4	10	18499351	G		7.17E-07	0.856	0.268	0.250	
		rs6508149	25	18	24103708	T		3.17E-07	0.754	0.140	0.268	
Lipid	mevalonolactone	rs13427590	292	2	197652097	T		6.32E-07	0.097	0.283	0.186	*BOLL,PLCL1*
		rs17356099	12	5	153763488	C	T	8.74E-07	0.162	0.190	0.286	
		rs4752744	11	11	1697036	G		2.71E-07	0.869	0.967	0.237	*MUC5B*
Lipid	octanoylcarnitine (C8)	rs73243695	3	4	24246161	C		2.97E-08	0.061	0.184	0.320	*PPARGC1A*
		rs9385987	21	6	142105107	T		1.73E-07	0.559	0.325	0.238	
Lipid	palmitoylcarnitine (C16)	rs142997609	3	1	166499198	T	T	4.33E-07	0.944	0.073	0.277	
		rs6585209	5	10	113127429	G		3.12E-07	0.193	0.192	0.226	
		rs2019758	22	21	27287894	T		2.52E-07	0.848	0.153	0.189	
Lipid	sphingomyelin (d17:1/16:0, d18:1/15:0, d16:1/17:0)	rs142997609	5	1	166499198	T	T	9.66E-09	0.171	0.149	0.304	
		rs146020378	3	2	161888149	A		9.80E-07		0.331	0.276	*DPP4*
		rs138929727	1	3	36867300	T		6.08E-07	0.391	0.259	0.333	*STAC*
		rs141454309	13	7	51320188	T		1.25E-07	0.839	0.204	0.275	
		rs10906537	13	10	13903714	A		8.31E-08	0.994	0.213	0.258	
		rs4776813	8	15	66654768	T		2.88E-07	0.239	0.142	0.191	*SMAD3,SMAD6,LINC01169*
Lipid	taurochenodeoxycholate	rs3003432	70	1	17272654	G		1.07E-07	0.019	0.074	0.291	
		rs10120987	38	9	83220251	T		6.83E-08	0.121	0.160	0.293	
		rs73606754	11	19	54420809	G		4.87E-07	0.415	0.165	0.414	
Amino	carnosine	rs34798713	211	1	240021314	A		9.40E-08	0.628	0.271	0.220	

acid		rs35659437	8	2	149221140	G		9.68E-07	0.179	0.200	0.305	
		rs1489955	3	4	177293157	T		5.90E-07	0.214	0.098	0.278	
Amino acid	dimethylglycine	rs10915298	9	1	4952241	A		9.92E-07	0.471	0.113	0.236	
		rs17732220	2	10	115992822	C		1.34E-07	0.642	0.218	0.233	
Amino acid	N-acetylarginine	rs9363451	29	6	65822620	A		2.93E-07	0.001	0.145	0.556	
		rs4901529	5	14	54685333	A		7.85E-07	0.256	0.169	0.254	
		rs117045903	2	15	66627247	A		2.19E-08	0.294	0.698	0.289	*SMAD3,SMAD6,LINC01169*
		rs8028010	8	15	94184482	A		9.14E-07	0.887	0.122	0.383	
Amino acid	N-acetylaspartate	rs28675771	1	4	670119	A		2.76E-07	0.891	0.099	0.285	
Carbohydrate	arabitol	rs2298110	34	1	19906558	G		1.14E-11	0.077	0.936	0.398	
		rs12406639	1	1	160467623	A		1.42E-07		0.319	0.206	
		rs72727620	3	1	175744164	G		8.76E-08	0.907	0.193	0.256	
		rs113812800	1	2	3901708	C		2.38E-07	0.053	0.738	0.383	*ALLC*
		rs192005306	10	2	32321366	T		1.17E-07	0.458	0.312	0.290	
		rs114592831	1	2	97791822	A		1.03E-07	0.698	0.170	0.289	
		rs112953992	3	2	101585155	C		4.89E-07	0.532	0.171	0.288	*IL1R1,IL1R2,LINC01127,MAP4K4*
		rs150822141	78	3	51647972	T		1.22E-07	0.638	0.227	0.194	*VPRBP,DOCK3*
		rs2679227	2	3	102139808	G	T	1.30E-07	0.043	0.358	0.350	*LOC152225*
		rs79408798	3	4	20951073	G		7.13E-07	0.801	0.177	0.261	
		rs114424030	9	4	87418455	T		6.42E-07	0.448	0.209	0.247	
		rs736577	4	4	138443464	A		7.98E-07	NA	0.224	0.350	
		rs143270861	2	4	182064534	A	T	2.61E-08	0.707	0.698	0.258	*MGC45800*
		rs78278314	7	5	8380739	G		1.50E-09	0.879	0.200	0.416	
		rs151089927	3	5	39162522	C		7.34E-07	0.363	0.141	0.215	
		rs540292551	5	5	45241358	A		4.42E-07	NA	0.159	0.245	
		rs114278957	12	5	50657115	G		1.07E-07	NA	0.331	0.247	
		rs72756725	11	5	62984752	C		6.77E-09	0.915	0.292	0.342	
		rs35864749	2	5	77411613	T		1.93E-07	0.164	0.145	0.315	

		rs146986595	3	5	137006796	T		4.24E-07	0.497	0.346	0.332	
		rs1629996	2	5	166339322	A		6.73E-08	0.098	0.145	0.230	
		rs185153229	7	6	34748997	C	T	7.02E-08	0.767	0.625	0.203	*TCP11,SCUBE3*
		rs76203979	3	6	168985885	C		8.26E-07	NA	0.188	0.231	
		rs76379349	16	8	11888466	C		1.99E-07	0.049	0.143	0.246	
		rs11786235	25	8	15760604	C		3.61E-07	0.348	0.489	0.238	
		rs142807487	2	8	86517435	G		1.72E-08	0.756	0.347	0.247	
		rs184036761	1	9	1933947	G	T	8.11E-08	0.437	0.184	0.211	*SMARCA2*
		rs12555626	5	9	38598373	T		2.53E-07	0.907	0.156	0.259	
		rs117086652	3	9	124602927	A		6.62E-07	NA	0.184	0.258	*NEK6*
		rs57579391	29	9	134280592	C		6.75E-07	0.256	0.238	0.218	
		rs77060617	4	12	4630227	G		3.59E-07	0.808	0.250	0.273	
		rs77838113	13	12	111527721	G		1.33E-07	0.682	0.271	0.322	*ATXN2,SH2B3, BRAP*
		rs112060328	28	12	112366683	T		5.92E-07	0.682	0.154	0.255	
		rs78908125	11	13	37460454	G		2.16E-07	0.774	0.191	0.247	
		rs115777460	1	13	52988125	A	T	6.56E-10	0.817	0.728	0.381	
		rs74105457	1	13	95195987	G		1.22E-07	0.976	0.220	0.243	
		rs77244206	7	15	31337658	G		7.57E-07	0.610	0.957	0.377	
		rs140445141	1	16	58342404	A		3.85E-07	0.734	0.139	0.258	
		rs138603619	1	17	78945892	T		7.93E-07	0.056	0.219	0.230	
		rs117099074	2	18	11041900	T		6.18E-07	0.769	0.155	0.451	
		rs122755652	2	18	73606273	T		1.27E-07	0.886	0.197	0.191	
Carbohydrate	arabonate/xylonate	rs6506040	6	18	3007418	A		2.74E-07	0.499	0.173	0.314	*LPIN2*
Carbohydrate	erythronate	rs7296992	28	12	100671461	G		8.79E-07	0.460	0.129	0.335	
		rs9526531	152	13	49239474	A		1.18E-07	0.706	0.124	0.468	
Carbohydrate	N-acetylneuraminate	rs13240649	7	7	3217341	A		8.70E-07	0.108	0.368	0.245	*CARD11*
		rs10101380	119	8	5147537	C		9.05E-07	0.760	0.586	0.187	*CSMD1*
		rs2463076	130	19	24285479	T		6.93E-07	0.670	0.134	0.262	
Cofactors and vitamins	alpha-tocopherol	rs142997609	3	1	166499198	T	T	3.47E-07	0.508	0.199	0.189	
		rs6585209	6	10	113127429	G		5.33E-07	0.350	0.074	0.271	

Cofactors and vitamins	nicotinate	rs17692499	1	2	211291536	A	9.20E-07	NA	0.169	0.314	*ERBB4*
Energy	aconitate [cis or trans]	rs148027659	4	10	74609570	G	5.80E-07	0.633	0.514	0.214	
Energy	malate	rs111269517	63	8	102494130	A	6.66E-07	0.285	0.216	0.236	
		rs2987533	138	13	48986378	A	3.93E-07	0.600	0.430	0.297	*CYSLTR2*
		rs9806917	1	16	23379535	T	2.64E-07	0.328	0.198	0.229	
Nucleotide	guanosine	rs10857843	21	1	110998913	C	5.07E-07	0.790	0.313	0.221	
		rs76439278	2	7	9104576	A	1.34E-07	0.036	0.119	0.370	
		rs11072489	257	15	74497063	A	7.17E-07	0.038	0.246	0.222	*CCDC33,CYP11A1*
Nucleotide	urate	rs649053	138	9	21387419	G	3.01E-08	0.572	0.133	0.273	*IFNA14,IFNA22P*
		rs3904254	122	13	69729260	A	9.83E-07	0.596	0.166	0.257	*KLHL1*
		rs8047421	198	16	50095354	G	5.91E-07	0.121	0.119	0.240	
**Significant in Non-Hispanic black sample**											
Lipid	1,2-dioleoyl-GPG (18:1/18:1)	rs145099041	50	1	101658118	G	0.776	3.79E-07	0.301	0.173	
		rs76968348	4	4	91057405	A	0.114	8.73E-07	0.242	0.125	
		rs146232470	1	4	167659452	G	0.023	9.20E-07	0.169	0.231	
		rs2453331	2	5	38042699	A	0.210	1.60E-08	0.164	0.176	*GDNF,EGFLAM*
		rs10110025	24	8	14516874	G	0.769	5.70E-07	0.202	0.214	
Lipid	13-HODE + 9-HODE	rs11576652	10	1	226098419	C	0.587	8.09E-07	0.479	0.262	
		rs1438301	22	5	12931189	C	0.383	7.65E-08	0.171	0.199	
		rs73658708	12	9	116883635	T	0.482	2.08E-08	0.132	0.213	
Lipid	1-linoleoyl-GPE (18:2)	rs12402427	7	1	87809464	T	0.828	5.95E-07	0.155	0.223	
		rs10922987	78	1	88016091	A	0.113	6.94E-07	0.218	0.439	
		rs9600435	97	13	75138763	G	0.361	4.10E-07	0.141	0.222	
Lipid	2-stearoyl-GPE (18:0)	rs3127565	32	1	48900036	C	0.429	5.27E-07	0.350	0.275	
		rs8192673	1	2	164497202	C	0.520	3.97E-09	0.149	0.232	
		rs12494581	36	3	55257122	C	0.524	7.25E-07	0.812	0.506	
		rs183022724	2	3	88153092	A	0.679	4.43E-07	0.155	0.421	

		rs55916950	2	4	181855793	T	T	0.460	5.09E-07	0.141	0.127	*MGC45800*
		rs36072781	8	6	23972206	A		0.158	9.67E-07	0.111	0.382	
		rs73419214	23	15	60265218	T		NA	6.59E-07	0.296	0.289	*RORA,ANXA2, FOXB1,NARG2*
		rs113420860	24	15	78959141	C		0.567	1.27E-07	0.301	0.162	
Lipid	docosapentaenoate (DPA; 22:5n3)	rs10399747	7	1	33021334	T		0.931	7.80E-08	0.191	0.245	
		rs72943450	5	1	78770792	A		0.793	7.52E-07	0.172	0.311	*IFI44,ELTD1*
		rs150733842	5	2	226684477	A		NA	3.84E-07	0.180	0.238	
		rs73578505	48	8	31863824	C		0.084	5.23E-07	0.198	0.495	
		rs12541487	7	8	88985505	A	T	0.195	3.88E-07	0.346	0.352	
		rs28621571	3	10	43716054	T		NA	7.29E-09	0.219	0.410	*RASGEF1A*
		rs868952478	2	10	50076899	T		0.174	5.21E-07	0.128	0.215	*SLC18A3*
		rs59931175	14	15	46028036	A		0.816	5.97E-07	0.165	0.226	
		rs73501411	7	16	8766907	A		NA	4.17E-07	0.166	0.345	*SQRDL*
Lipid	glutarate (C5-DC: glutarylcarnitine)	rs7524634	21	1	27313372	T		0.404	3.40E-07	0.231	0.200	
		rs12651261	2	4	154049599	G		0.296	1.73E-07	0.201	0.334	
		rs1845656	56	5	97278401	C		0.749	9.66E-07	0.206	0.178	
		rs113655051	28	10	43236475	A		0.456	6.76E-07	0.283	0.248	*RASGEF1A*
		rs8073779	6	17	4773788	A		0.142	9.40E-08	0.390	0.366	
Lipid	glycerophosphoinositol	rs56126228	96	4	97234743	G		0.399	3.56E-07	0.137	0.158	
		rs72809658	47	5	114989394	A		0.073	1.64E-07	0.343	0.308	
		rs9366421	5	6	22017809	G	T	0.741	4.49E-07	0.143	0.248	*CASC15*
		rs13044395	5	20	4634333	G		0.022	7.63E-07	0.305	0.132	*SMOX*
Lipid	mevalonolactone	rs7811174	70	7	17425006	A		0.135	9.13E-07	0.218	0.132	
		rs7031683	22	9	7594949	A		0.665	1.78E-07	0.160	0.281	
		rs61894457	11	11	97870174	G		0.212	9.65E-08	0.197	0.226	
Lipid	octanoylcarnitine (C8)	rs55920039	7	3	42047516	T		0.415	5.98E-07	0.246	0.319	
		rs113887828	22	5	37959362	A		0.323	6.01E-07	0.201	0.268	*GDNF,EGFLAM*
		rs9496853	29	6	144078903	G		NA	4.32E-07	0.182	0.121	*UTRN*
		rs58819299	11	14	72664447	T		0.338	5.50E-09	0.223	0.244	
		rs6650552	2	15	101086762	G		NA	6.81E-07	0.136	0.290	
		rs1741325	4	20	4181543	G		0.599	2.92E-07	0.152	0.177	*SMOX*

Lipid	palmitoylcarnitine (C16)	rs78340409	31	1	234553708	G		0.943	8.19E-08	0.169	0.245	
		rs79141561	4	2	105989161	G		NA	8.10E-07	0.550	0.472	
		rs9484844	22	6	144102979	G		0.461	6.78E-07	0.142	0.407	*UTRN*
		rs9534251	6	13	45926160	T		0.193	3.25E-07	0.236	0.256	
Lipid	phosphocholine	rs10649	6	1	201134866	A		0.547	4.59E-08	0.182	0.222	*TMEM9,IGFN1*
		rs11633493	13	15	59543398	G		0.209	4.39E-08	0.262	0.299	*FOXB1*
		rs78250794	6	17	4568774	T		0.151	2.44E-07	0.218	0.251	
		rs73960527	11	18	56138709	A		0.749	2.77E-07	0.213	0.316	
Lipid	sphingomyelin (d17:1/16:0, d18:1/15:0, d16:1/17:0)	rs12752641	8	1	203053298	C		0.475	5.04E-07	0.688	0.226	*ADORA1,PPFIA4,MYOG,MYBPH,CHI3L1,CHIT1*
		rs78340409	29	1	234553708	G		0.852	1.32E-07	0.659	0.164	
		rs11153989	49	6	121017082	C		0.947	1.19E-07	0.245	0.485	
		rs77499805	11	13	98753079	A		0.563	4.09E-07	0.183	0.468	*UBAC2,DOCK9,UBAC2-AS1*
		rs8107764	1	19	7722032	A	T	0.437	6.54E-07	0.178	0.254	*INSR*
		rs13047185	8	21	34126118	C		0.520	7.04E-07	0.401	0.253	
Lipid	taurochenodeoxycholate	rs76403141	2	6	33997776	C	T	0.799	2.16E-07	0.283	0.254	*GRM4,IP6K3,ITPR3,LEMD2,MLN,UQCC2*
		rs6477421	6	9	993546	G	T	0.823	1.43E-07	0.263	0.138	
		rs72816230	7	17	1219933	G		0.023	6.15E-07	0.626	0.249	
Amino acid	3-methyl-2-oxobutyrate	rs9825362	110	3	102692907	A	T	0.977	4.75E-07	0.168	0.292	
		rs34045937	25	4	171154743	T		0.915	6.83E-07	0.135	0.288	
		rs115652568	1	6	12441062	C		0.659	8.01E-07	0.166	0.258	*HIVEP1*
		rs9496838	29	6	144018802	G		NA	3.32E-08	0.214	0.302	*UTRN*
		rs10966219	2	9	24109010	C		0.730	6.02E-07	0.190	0.223	*ELAVL2*
		rs76625879	10	11	124076797	C		0.410	1.60E-07	0.204	0.289	*OR6X1*
		rs9961696	3	18	28289208	C		0.240	9.59E-07	0.151	0.170	
Amino acid	carnosine	rs59692785	32	2	217757789	G		0.567	4.80E-07	0.202	0.132	*TNS1*

		rs112362780	1	17	37723113	A		NA	4.15E-07	0.346	0.203	
Amino acid	dimethylglycine	rs12186076	299	3	41793147	C		0.462	8.94E-07	0.184	0.196	
		rs4466711	22	10	67601733	C		0.457	2.21E-07	0.142	0.123	*CTNNA3*
		rs8052562	1	16	3491830	T		0.436	7.21E-07	0.686	0.268	*TRAP1*
		rs16948404	26	17	13746314	A		0.022	5.54E-07	0.182	0.223	
Amino acid	N-acetylarginine	rs61333185	29	10	59865672	C		0.875	5.26E-07	0.184	0.252	
		rs199593963	7	19	52710930	T		0.878	8.62E-07	0.229	0.176	*ZNF665*
Amino acid	N-acetylaspartate	rs74341935	24	3	16723792	T		0.761	3.66E-07	0.130	0.179	*PLCL2*
		rs11714340	64	3	27610676	C		0.211	5.01E-07	0.581	0.220	
		rs78310529	36	17	53196962	A		0.472	6.24E-07	0.235	0.365	
Carbohydrate	arabitol	rs2216946	8	2	217271551	C		0.624	4.66E-07	0.157	0.121	
		rs1400275	218	3	145351415	A		0.444	3.68E-07	0.170	0.208	
		rs111672193	13	10	34060697	A		NA	6.12E-07	0.161	0.194	
		rs12436940	151	14	31741232	T		0.253	9.79E-07	0.237	0.183	*ARHGAP5*
Carbohydrate	arabonate/xylonate	rs12123801	33	1	166592258	G	T	0.029	8.04E-07	0.249	0.266	
		rs35514775	13	1	183573732	G		NA	7.36E-07	0.170	0.226	
		rs58973984	5	3	60129706	C		0.782	3.52E-07	0.195	0.296	
		rs546847929	6	5	25368397	G		0.350	7.71E-07	0.142	0.265	
Carbohydrate	erythronate	rs11194146	7	10	108525183	C		0.142	8.82E-07	0.307	0.233	
		rs62079267	2	18	5614610	C		0.227	8.16E-07	0.203	0.252	
Carbohydrate	N-acetylneuraminate	rs17047802	10	2	56325870	C		0.176	4.70E-07	0.206	0.231	
		rs148846446	7	3	65997183	C		0.508	4.66E-07	0.250	0.145	*MAGI1*
		rs2077681	28	6	3085866	C		0.747	7.04E-07	0.201	0.302	*MYLK4*
		rs6597167	19	6	5817232	C		0.554	9.48E-07	0.238	0.247	*FARS2*
		rs28661076	3	7	19504200	G		0.645	7.29E-07	0.192	0.251	*TWIST1,FERD3L,LOC101927668*
		rs76840346	84	13	53885705	C		0.063	3.50E-07	0.511	0.272	
		rs4795778	10	17	33403050	T		0.766	4.38E-07	0.215	0.254	
Cofactors and vitamins	nicotinate	rs17855475	33	1	180178877	C		0.580	8.06E-07	0.441	0.458	*XPR1*
		rs62242629	3	3	8735624	A		0.932	5.88E-07	0.175	0.304	
		rs114696228	5	3	33824840	A		NA	6.80E-07	0.125	0.140	

		rs1205603	36	10	52405013	A	0.125	8.07E-07	0.208	0.223	*PRKG1*
Cofactors and vitamins	alpha-tocopherol	rs4471729	37	17	49350803	T	0.296	3.67E-07	0.215	0.131	*ZNF652,PHB,ABI3,FLJ40194, GNGT2,LOC102724596,MIR6129,PHOSPHO1,LOC101927207*
		rs12942941	13	17	80129214	G	0.476	8.98E-07	0.652	0.239	
Energy	aconitate [cis or trans]	rs1173589	57	1	53707271	G	0.496	1.46E-07	0.157	0.116	
		rs12632521	29	3	32682166	A	0.920	1.30E-07	0.667	0.219	*GLB1,TRIM71, TMPPE,CRTAP,SUSD5,CCR4*
		rs4595640	24	12	107133235	A	0.082	5.69E-09	0.148	0.284	
		rs8013826	83	14	25784166	T	0.113	3.96E-07	0.129	0.180	
Energy	malate	rs3824716	8	10	76991052	A	0.018	8.17E-07	0.195	0.261	
Nucleotide	guanosine	rs74527054	3	2	173412629	T	0.840	4.14E-07	0.227	0.236	*CDCA7*
		rs112308097	27	2	230821995	T	NA	8.67E-08	0.576	0.301	
		rs1534656	35	4	170416250	A	NA	4.70E-07	0.254	0.239	
Nucleotide	urate	rs1816709	7	2	153327843	C	0.454	5.22E-07	0.141	0.160	
		rs79282949	35	3	66418398	A	0.091	8.27E-07	0.492	0.251	*MAGI1*
		rs1512919	86	3	86270519	A	0.725	6.83E-07	0.184	0.177	
		rs2351752	20	5	154588462	T	0.894	9.86E-08	0.361	0.205	
		rs12002545	3	9	101282604	A	0.905	7.43E-07	0.177	0.273	*GRIN3A*
		rs9511396	12	13	24657064	A	0.614	6.38E-07	0.186	0.244	
		rs77780840	16	14	63422807	C	0.412	9.19E-07	0.142	0.156	
		rs112362780	4	17	37723113	A	NA	2.58E-07	0.401	0.203	

* The SNP with the smallest (i.e., most significant) P value in each clumped region in the mtQTL analysis.

† The number of SNPs with a P value of < 1 × 10-3 in each clumped region in the mtQTL analysis.

‡ Whether the locus region overlapped with the other loci showed significance in another racial/ethnic sample (i.e., Non-Hispanic white or Non-Hispanic black). The word "T" means TRUE, and the blank means FALSE.

§ The genetic loci with PP.H4 ≥0.5 were considered to colocalize with the external asthma GWAS dataset.

Abbreviations: Alt, alternative; Chr, chromosome; CAAPA, consortium on asthma among African-ancestry populations in the Americas; DPA, docosapentaenoate; GPE, glycerophosphorylethanolamine; GPG, glycerophosphoglycerol; HODE, hydroxyoctadecadienoic acid; KB, kilobyte; PP.H4, the posterior probability of H4; SNP, single nucleotide polymorphism; UKB, UK Biobank

Table S6. Summary of the genetic loci associated with the candidate metabolites in Hispanic sample

Metabolite		Locus						
**Metabolite class**	**Metabolite name**	**Index SNP***	**N**	**Chr**	**Index SNP* position**	**Alt allele**	**P value**	**Known asthma genes within index SNP* ± 500KB**
Lipid	1,2-dioleoyl-GPG	rs77707953	79	3	77252161	T	7.14E-07	
		rs72952414	5	18	66454088	G	5.33E-07	
Lipid	13-HODE + 9-HODE	rs2036184	161	4	28344153	T	7.28E-07	
		rs367705986	5	6	17015501	A	4.41E-07	
		rs79412944	11	11	131282274	C	8.46E-07	
Lipid	1-linoleoyl-GPE	rs73259353	9	12	5478963	T	9.55E-08	
Lipid	2-stearoyl-GPE	rs59571924	6	4	76815268	G	1.02E-07	
		rs111228844	9	5	172870793	G	3.13E-07	
		rs111815842	8	7	35499860	T	4.01E-07	
		rs1550702	3	8	125793575	T	7.00E-07	
		rs553908847	1	11	36144085	T	8.91E-07	
Lipid	docosapentaenoate	rs290660	44	15	95190280	G	6.91E-07	
		rs149793817	7	17	49947386	T	4.91E-08	
Lipid	glutarate	rs2123356	18	10	4786104	T	9.91E-07	
Lipid	mevalonolactone	rs13331682	143	16	81186989	A	6.47E-08	
Lipid	octanoylcarnitine	rs6822497	93	4	28789794	T	2.84E-07	
		rs12520495	6	5	5802458	G	6.26E-07	
		rs62426461	81	6	78517273	G	8.61E-07	
Lipid	palmitoylcarnitine	rs12073468	35	1	225378844	T	3.21E-07	
		rs8034787	14	15	72856004	T	1.13E-07	
Lipid	phosphocholine	rs73330786	4	8	121950682	A	8.97E-07	
Lipid	sphingomyelin	rs55784498	20	2	99577270	T	9.78E-07	
		rs6822497	87	4	28789794	T	5.27E-07	
		rs34305344	31	9	31007281	G	1.94E-08	

Lipid	taurochenodeoxycholate	rs7813257	21	8	133987675	T	8.78E-07	
Amino acid	carnosine	rs2402353	171	7	118385709	A	3.03E-07	
		rs151119306	2	11	31775873	A	4.57E-07	
		rs9602501	1	13	84551177	G	8.82E-07	
		rs6132364	37	20	2168749	C	9.47E-07	
Amino acid	dimethylglycine	rs1611121	12	9	133642514	T	2.62E-07	
Amino acid	N-acetylarginine	rs77624317	4	5	165118661	G	4.53E-07	
		rs115091720	63	8	9682860	T	4.16E-07	*MSRA,TNKS*
		rs73493952	186	11	75943132	G	9.62E-08	
		rs77779262	1	18	23248067	A	1.12E-08	
Amino acid	N-acetylaspartate	rs10925401	18	1	237393830	T	5.12E-07	
		rs16842265	13	1	165300718	T	4.34E-07	
		rs13058923	19	3	118880181	T	5.63E-07	
		rs991097	43	4	155268801	T	9.68E-08	
Carbohydrate	arabitol	rs114678531	35	2	234139032	A	3.28E-08	
		rs12987450	19	2	17163692	C	7.76E-07	
		rs6741107	88	2	38425430	A	8.45E-07	
		rs79607794	1	12	128583749	T	7.43E-07	
		rs117607952	2	14	62757867	A	9.19E-07	
		rs77933572	10	16	64642752	G	6.75E-07	
Carbohydrate	arabonate/xylonate	rs2235528	4	1	22127147	T	2.44E-07	
		rs4682083	38	3	112089263	C	5.79E-07	
Carbohydrate	erythronate	rs848503	27	2	36430521	G	8.11E-07	*CRIM1*
		rs7686496	13	4	112420432	A	9.30E-07	
		rs4358579	2	5	145745270	G	8.17E-07	
Carbohydrate	N-acetylneuraminate	rs4680275	8	3	151252370	A	5.65E-07	
		rs56300419	74	12	99418551	C	4.87E-07	
		rs78648300	140	12	44718595	A	9.49E-07	
Cofactors and	alpha-tocopherol	rs187868016	9	1	57010337	G	8.15E-07	

Vitamins		rs3131643	28	6	31475005	A	6.85E-07	*HLA-B,HLA-C,MICA,ABHD16A,AIF1,APOM,ATP6V1G2,ATP6V1G2-DDX39B,BAG6,C6orf15,C6orf25,C6orf47,C6orf48,CCHCR1,CDSN,CLIC1,CSNK2B,DDAH2,DDX39B,GPANK1,HCG26,HCG27,HCP5,HSPA1A,HSPA1B,HSPA1L,LSM2,LST1,LTA,LTB,LY6G5B,LY6G5C,LY6G6C,LY6G6D,LY6G6E,LY6G6F,MCCD1,MICB,MIR4646,MIR6832,MIR6891,MSH5,MSH5-SAPCD1,NCR3,NEU1,NFKBIL1,POU5F1,PRRC2A,PSORS1C1,PSORS1C2,PSORS1C3,SAPCD1,SLC44A4,SNORA38,SNORD48,SNORD52,SNORD84,SNORD117,TCF19,TNF,VARS,VWA7,C2,EHMT2,ZBTB12*
		rs7047158	8	9	126060097	A	5.41E-07	
		rs56085832	8	18	2823279	A	6.57E-07	
Cofactors and vitamins	nicotinate	rs148743352	5	1	247428724	A	1.03E-07	
		rs6679474	10	1	4271686	A	1.83E-07	

		rs12380473	39	9	11133835	C	4.05E-07
		rs78257259	6	9	7064185	A	5.16E-07
		rs112252987	4	22	31888427	C	4.05E-07
		rs71314757	8	22	21002830	A	4.67E-07
Energy	malate	rs6822497	80	4	28789794	T	2.94E-07
		rs1450613	24	5	166474697	G	1.92E-07
		rs61970044	26	13	103711532	T	2.73E-07
		rs7339054	15	13	103139610	C	8.40E-07
Nucleotide	guanosine	rs494367	15	1	14209320	T	8.59E-07
Nucleotide	urate	rs73106062	17	5	73294746	C	5.82E-07

* The SNP with the smallest (i.e., most significant) P value in each clumped region in the mtQTL analysis.
Abbreviations: Alt, alternative; Chr, chromosome; DPA, docosapentaenoate; HODE, Hydroxyoctadecadienoic acid; GPE, glycerophosphorylethanolamine; GPG, glycerophosphoglycerol; KB, kilobyte; PP.H4, posterior probability of H4; SNP, single nucleotide polymorphism; UKB, UK Biobank

Figure S1. Manhattan plot and QQ plot of the mtQTL analysis for each of the 28 candidate metabolites

A. Non-Hispanic white sample

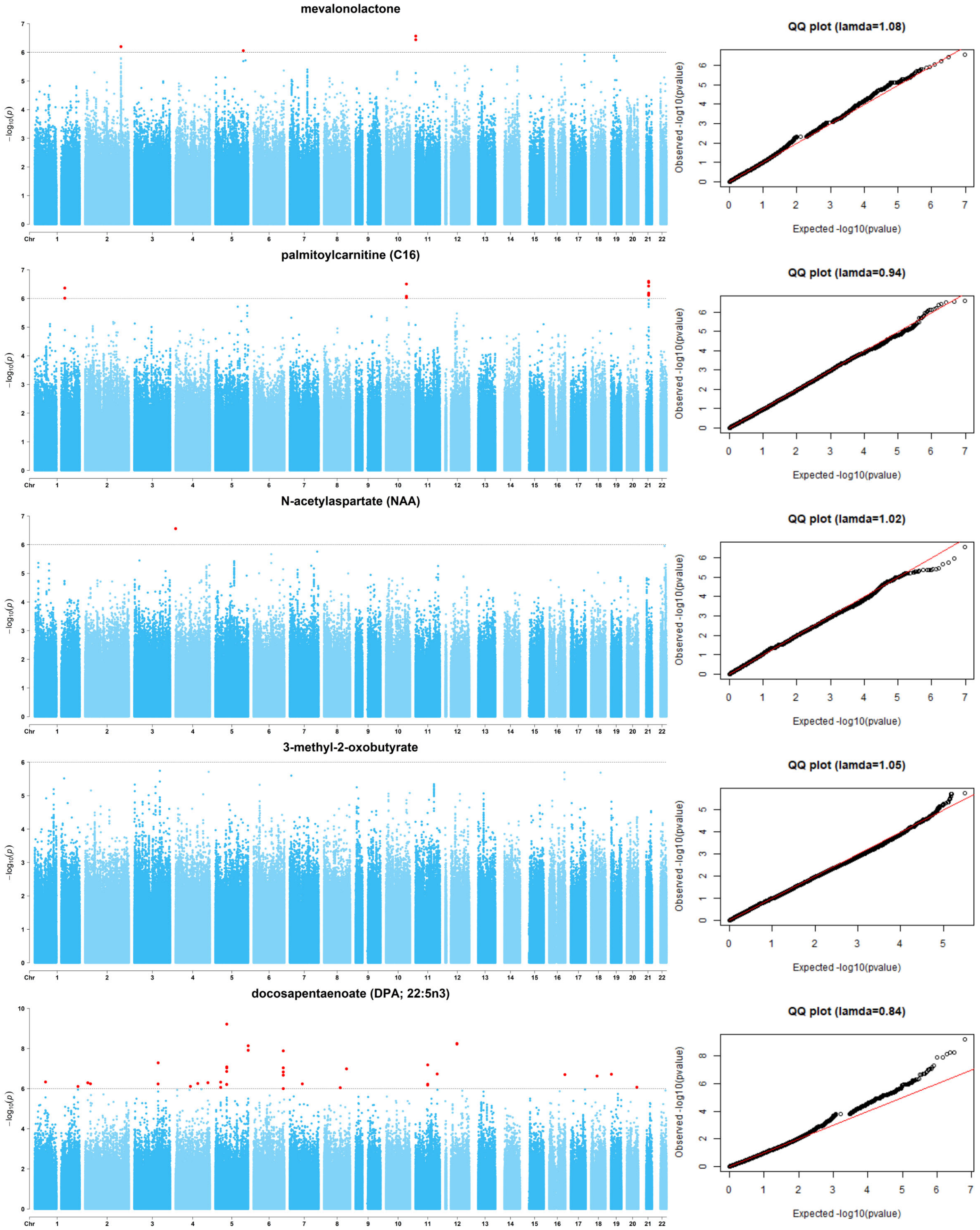

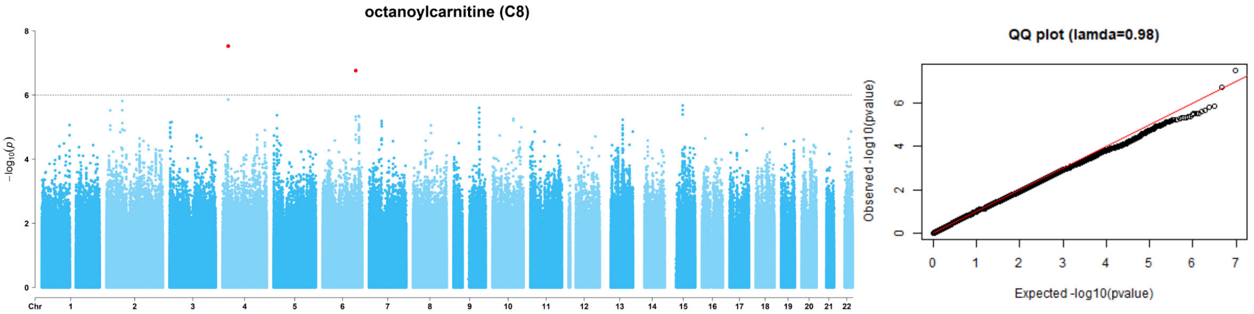
octanoylcarnitine (C8)
QQ plot (lamda=0.98)
Expected -log10(pvalue)
Observed -log10(pvalue)

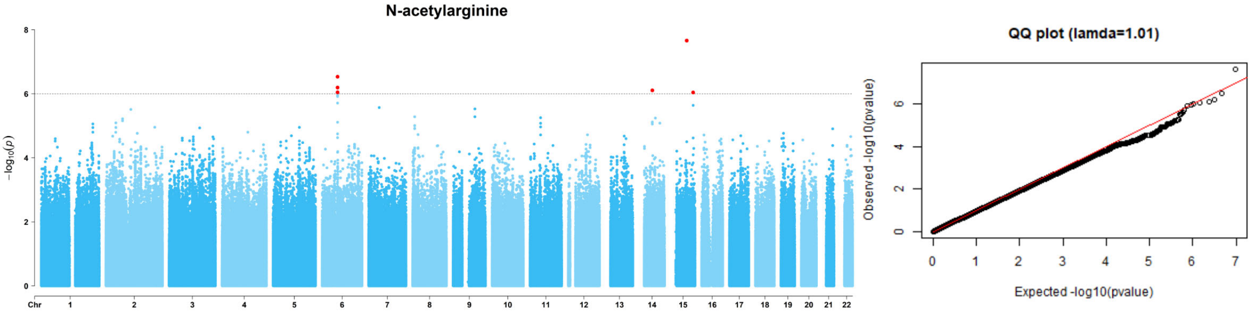
N-acetylarginine
QQ plot (lamda=1.01)
Expected -log10(pvalue)
Observed -log10(pvalue)

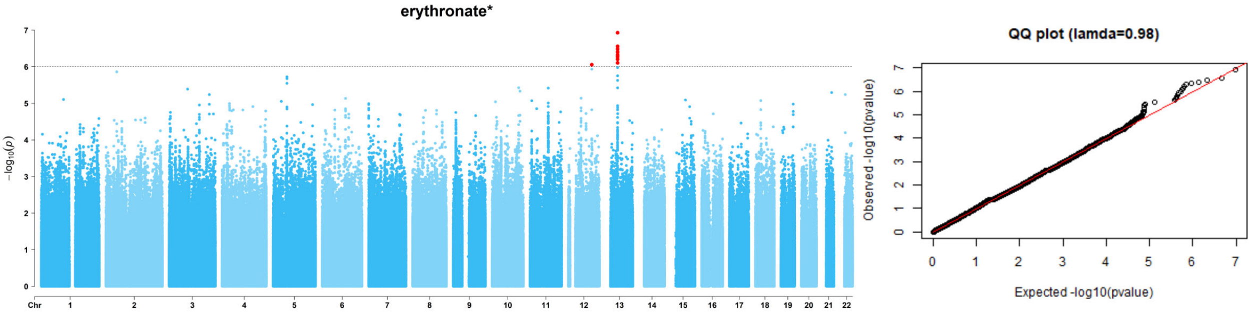
erythronate*
QQ plot (lamda=0.98)
Expected -log10(pvalue)
Observed -log10(pvalue)

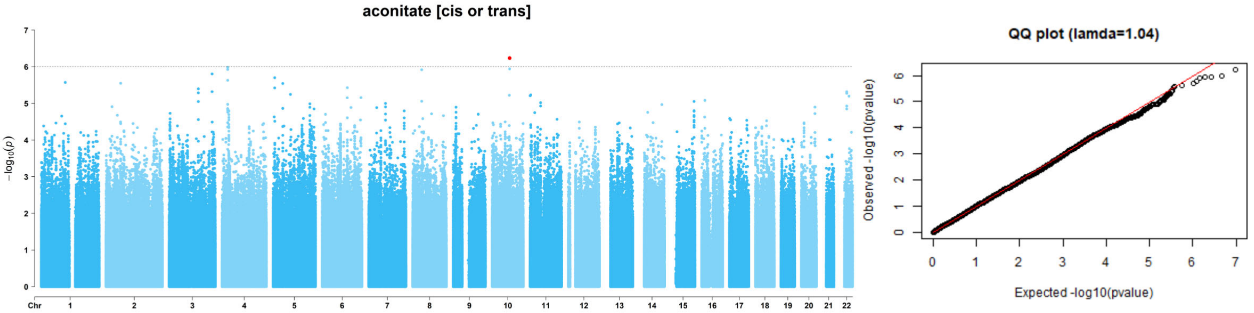
aconitate [cis or trans]
QQ plot (lamda=1.04)
Expected -log10(pvalue)
Observed -log10(pvalue)

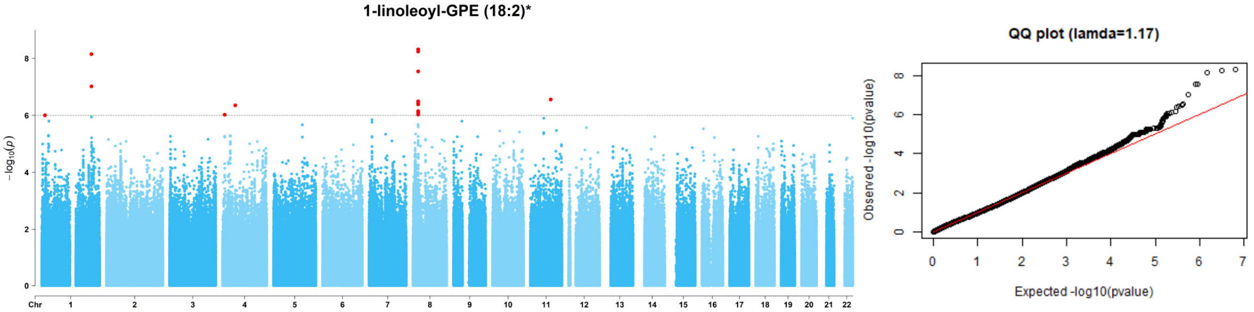
1-linoleoyl-GPE (18:2)*
QQ plot (lamda=1.17)
Expected -log10(pvalue)
Observed -log10(pvalue)

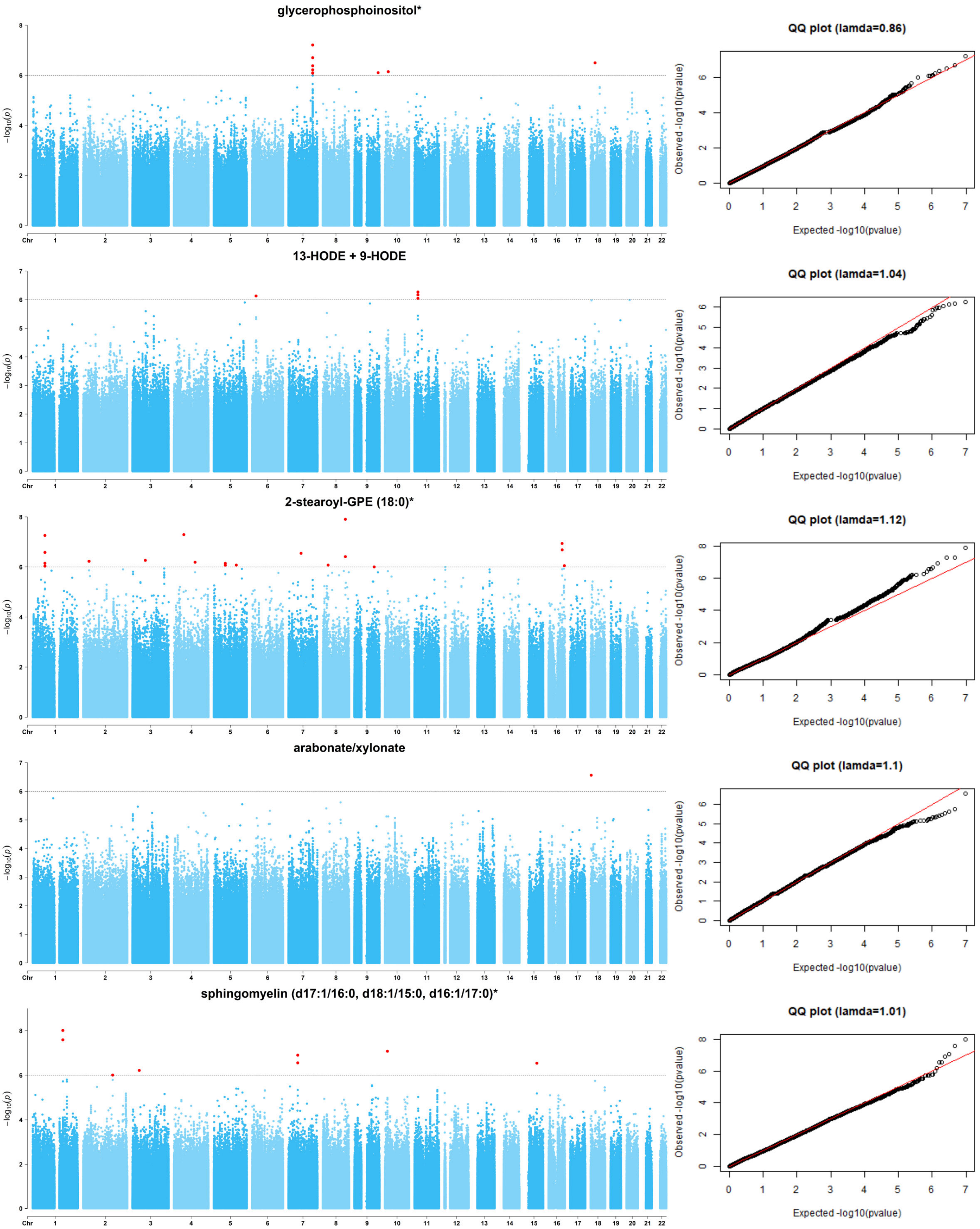
glycerophosphoinositol*
QQ plot (lamda=0.86)
13-HODE + 9-HODE
QQ plot (lamda=1.04)
2-stearoyl-GPE (18:0)*
QQ plot (lamda=1.12)
arabonate/xylonate
QQ plot (lamda=1.1)
sphingomyelin (d17:1/16:0, d18:1/15:0, d16:1/17:0)*
QQ plot (lamda=1.01)
$-\log_{10}(p)$
Chr
Observed -log10(pvalue)
Expected -log10(pvalue)

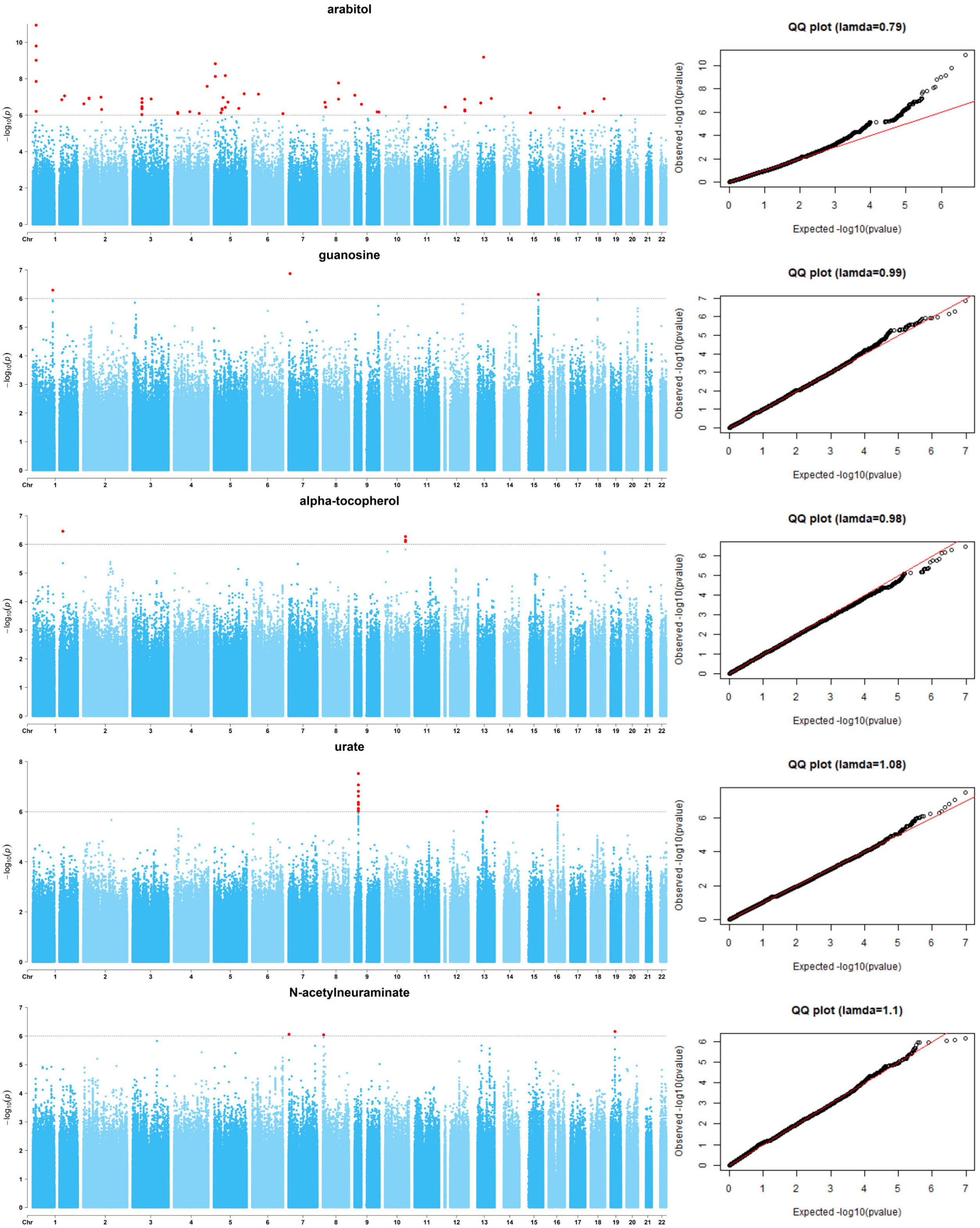
arabitol
QQ plot (lamda=0.79)
guanosine
QQ plot (lamda=0.99)
alpha-tocopherol
QQ plot (lamda=0.98)
urate
QQ plot (lamda=1.08)
N-acetylneuraminate
QQ plot (lamda=1.1)
$-\log_{10}(p)$
Chr
Observed -log10(pvalue)
Expected -log10(pvalue)

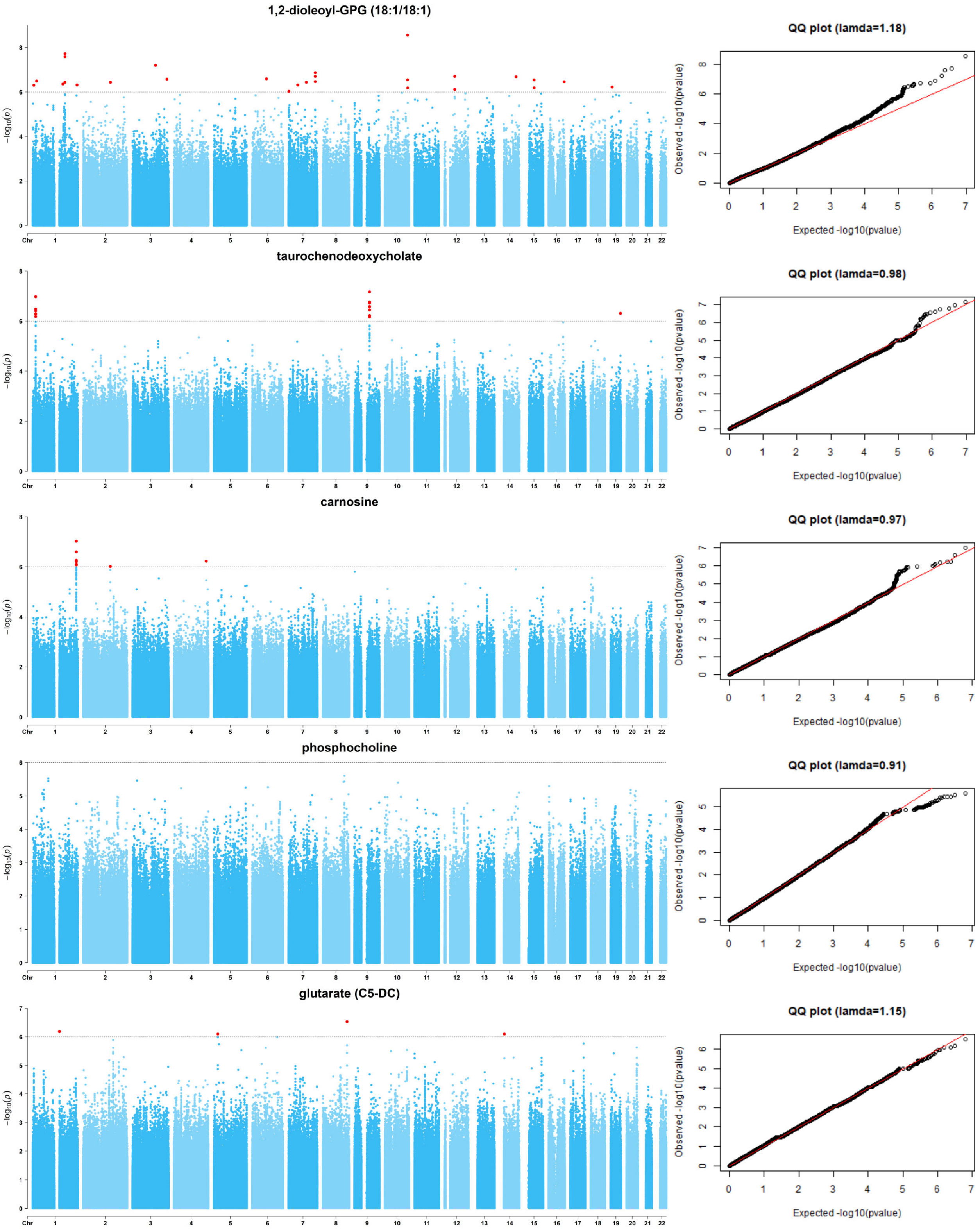
1,2-dioleoyl-GPG (18:1/18:1)
QQ plot (lamda=1.18)
taurochenodeoxycholate
QQ plot (lamda=0.98)
carnosine
QQ plot (lamda=0.97)
phosphocholine
QQ plot (lamda=0.91)
glutarate (C5-DC)
QQ plot (lamda=1.15)
$-\log_{10}(p)$
Chr
Observed -log10(pvalue)
Expected -log10(pvalue)

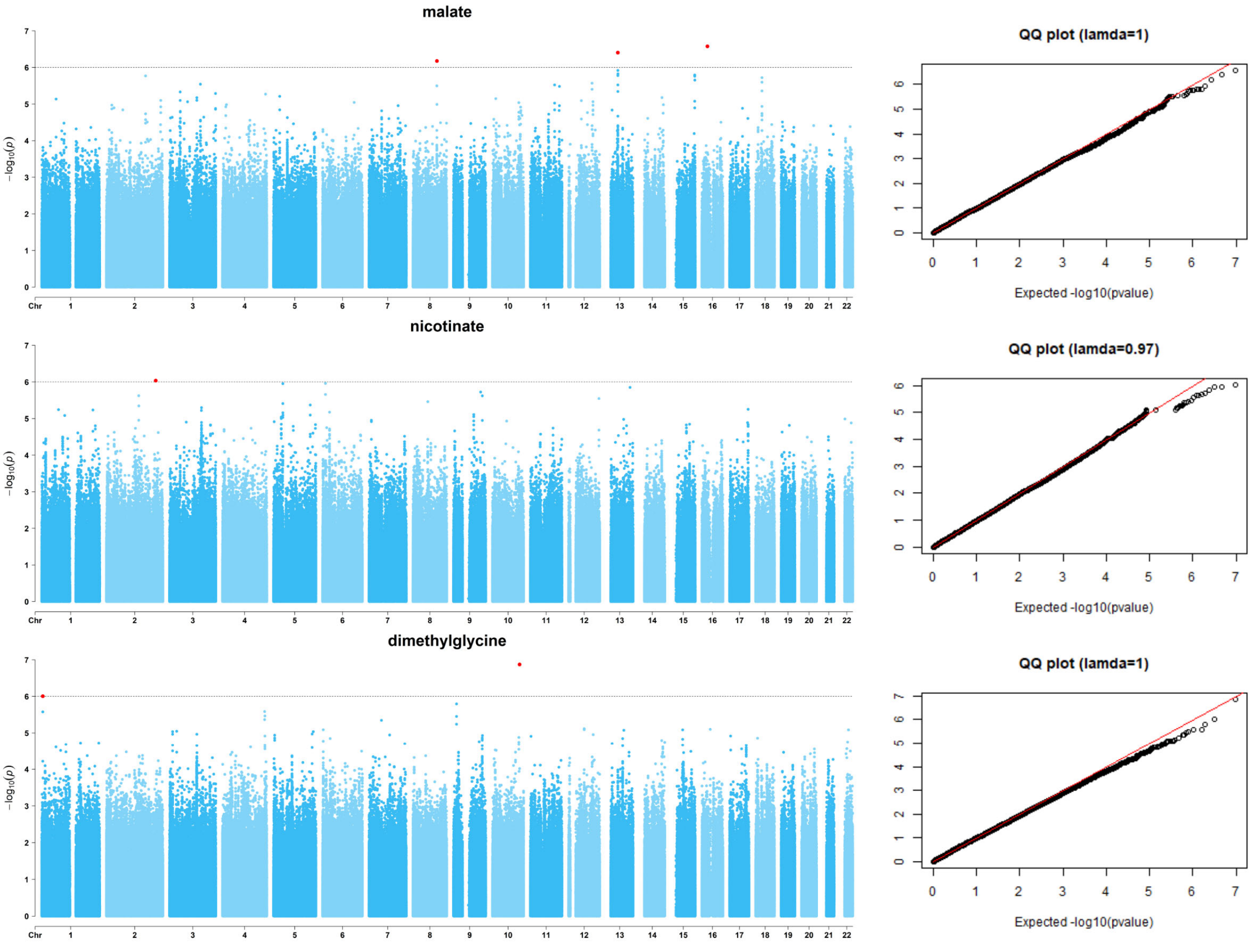
malate
QQ plot (lamda=1)
Expected -log10(pvalue)
nicotinate
QQ plot (lamda=0.97)
Expected -log10(pvalue)
dimethylglycine
QQ plot (lamda=1)
Expected -log10(pvalue)
−log10(p)
Chr

B. Non-Hispanic black sample

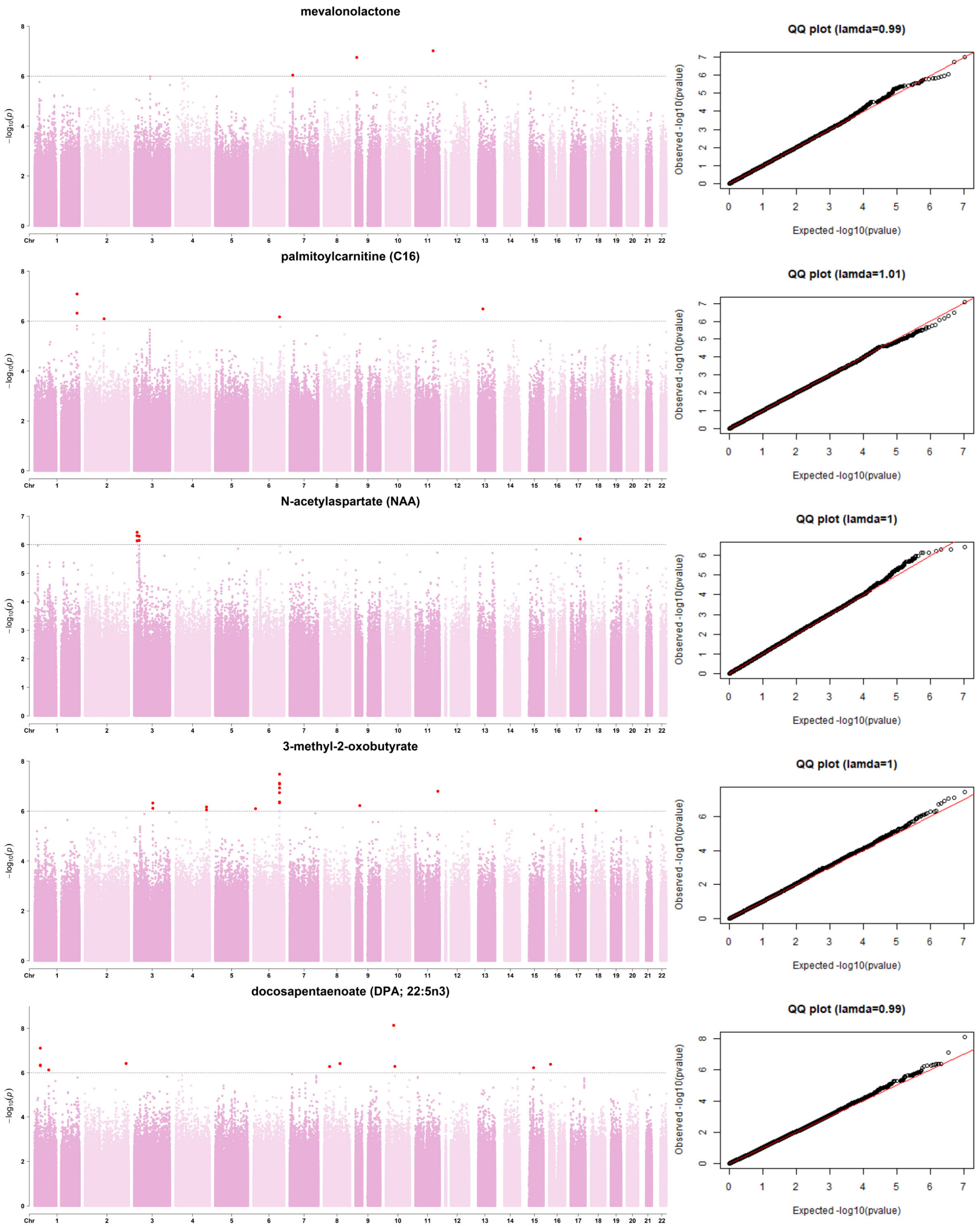

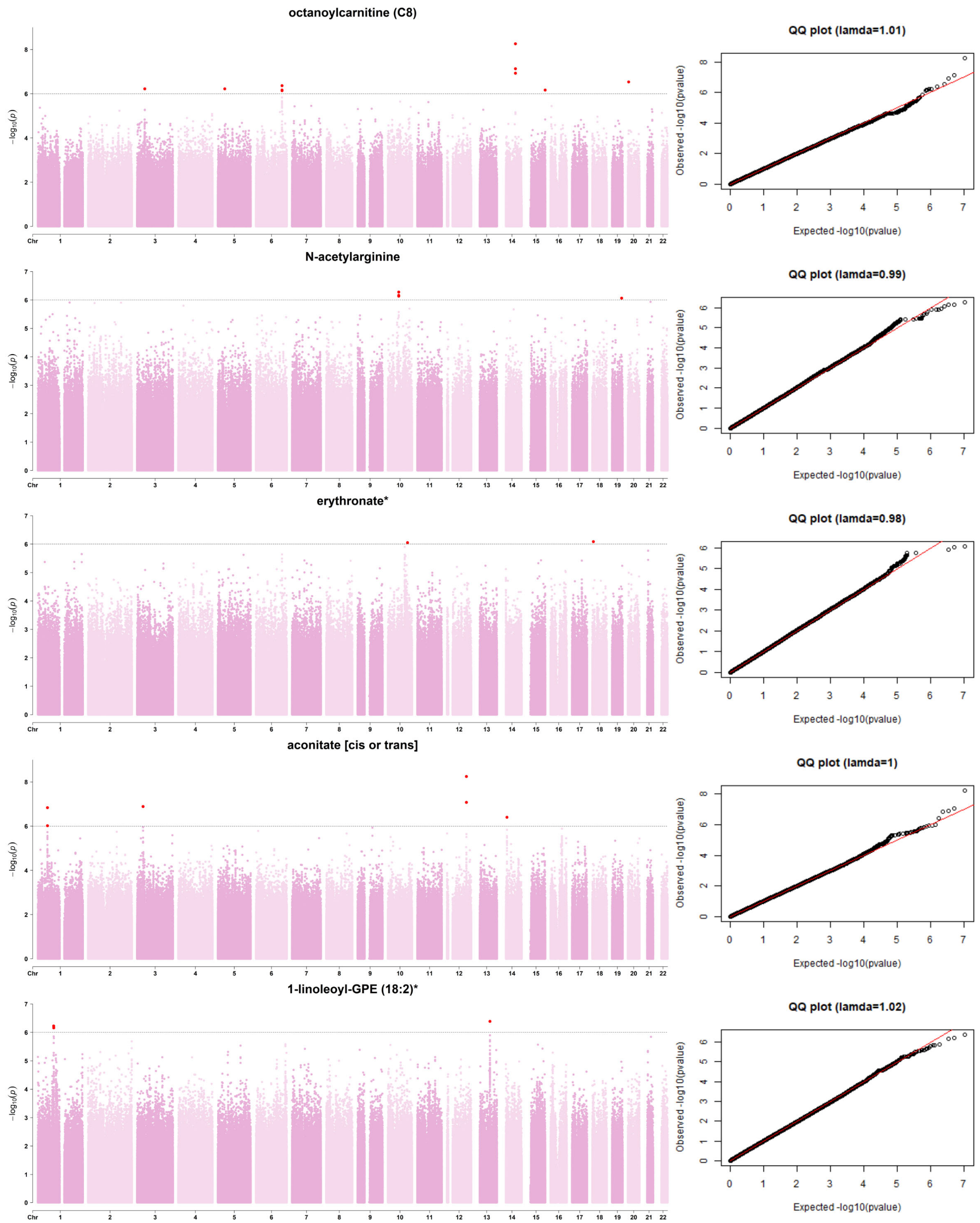
octanoylcarnitine (C8)
QQ plot (lamda=1.01)
N-acetylarginine
QQ plot (lamda=0.99)
erythronate*
QQ plot (lamda=0.98)
aconitate [cis or trans]
QQ plot (lamda=1)
1-linoleoyl-GPE (18:2)*
QQ plot (lamda=1.02)
$-\log_{10}(p)$
Chr
Observed -log10(pvalue)
Expected -log10(pvalue)

glycerophosphoinositol*

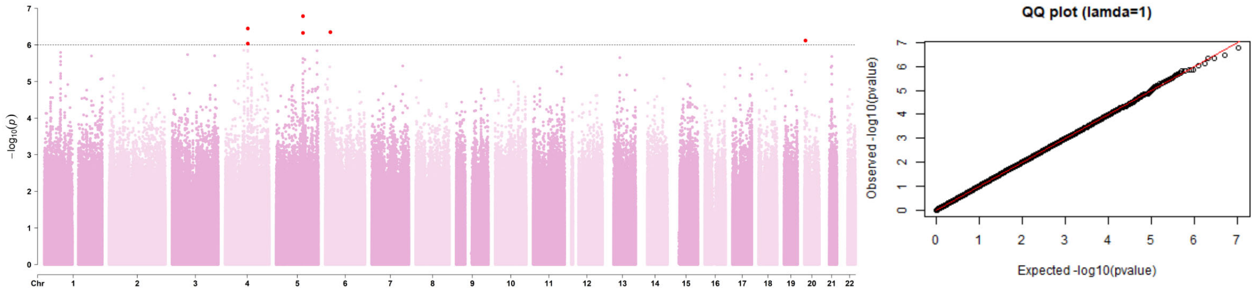

13-HODE + 9-HODE

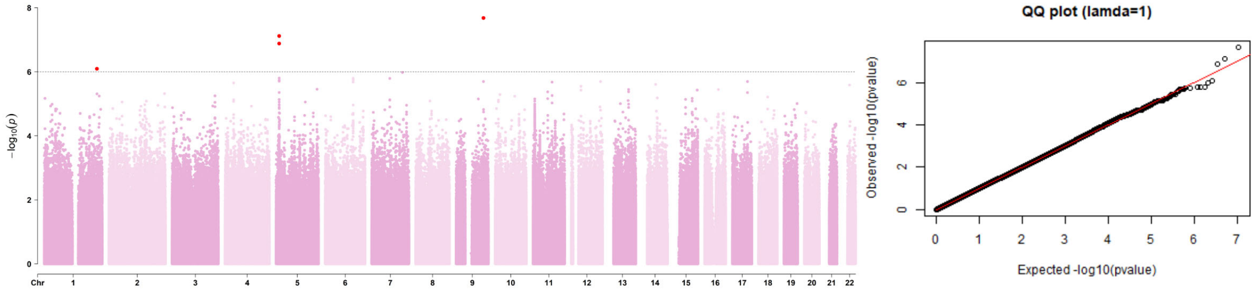

2-stearoyl-GPE (18:0)*

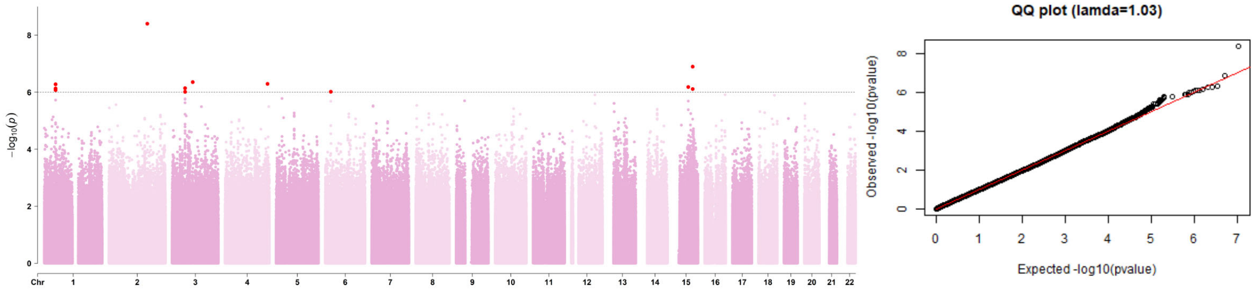

arabonate/xylonate

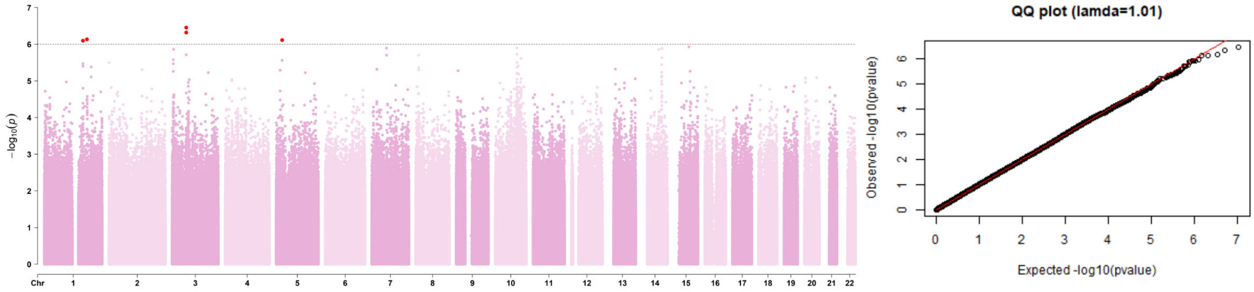

sphingomyelin (d17:1/16:0, d18:1/15:0, d16:1/17:0)*

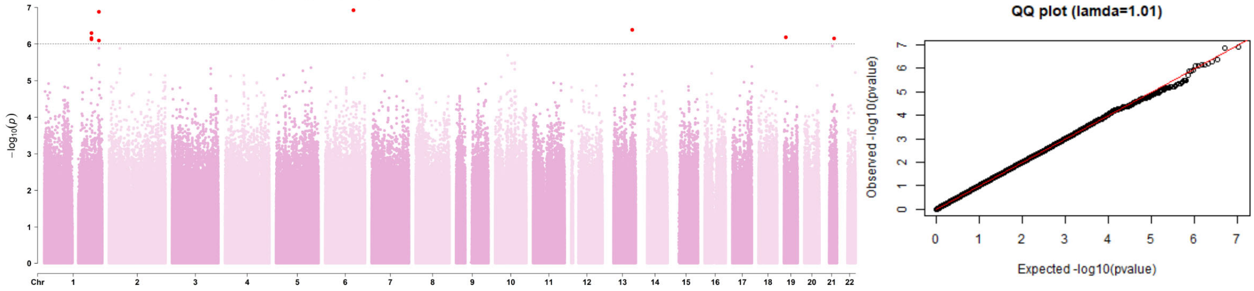

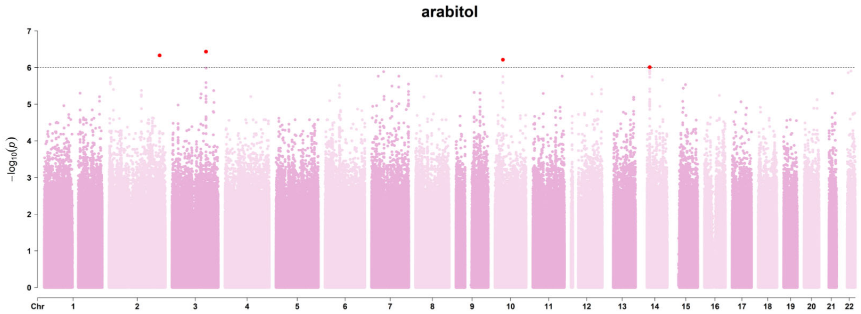
arabitol
$-\log_{10}(p)$
Chr

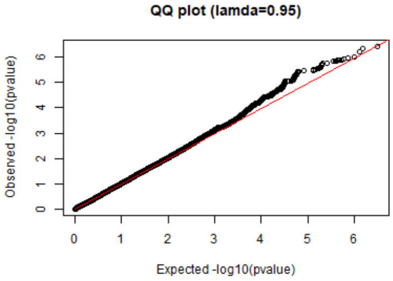
QQ plot (lamda=0.95)
Observed -log10(pvalue)
Expected -log10(pvalue)

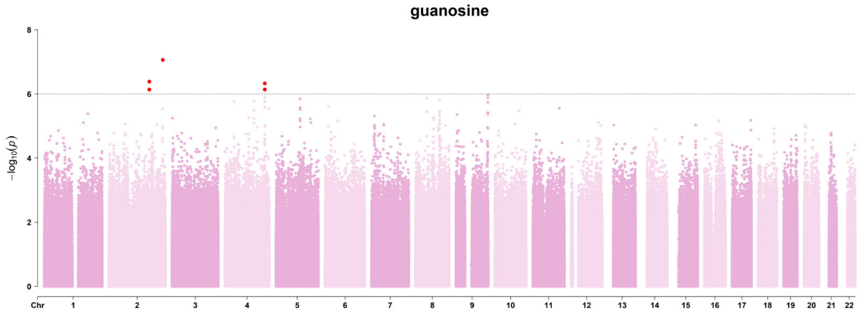
guanosine
$-\log_{10}(p)$
Chr

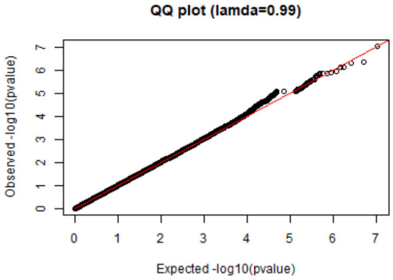
QQ plot (lamda=0.99)
Observed -log10(pvalue)
Expected -log10(pvalue)

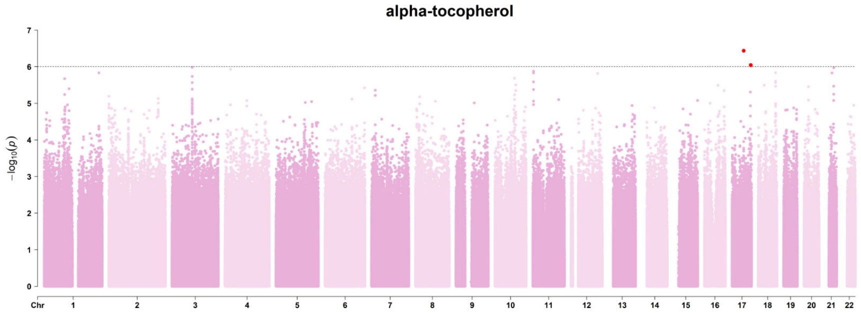
alpha-tocopherol
$-\log_{10}(p)$
Chr

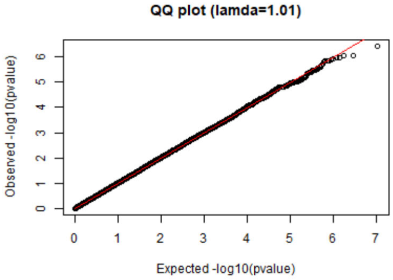
QQ plot (lamda=1.01)
Observed -log10(pvalue)
Expected -log10(pvalue)

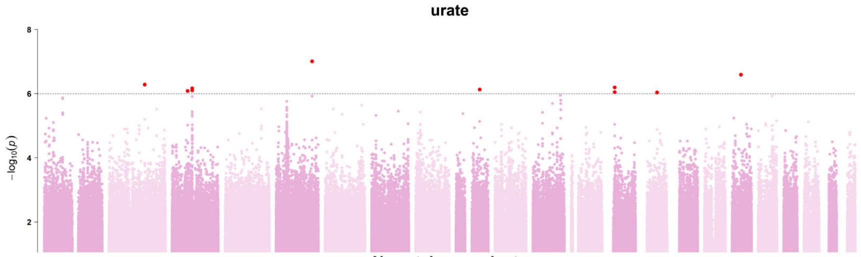
urate
$-\log_{10}(p)$

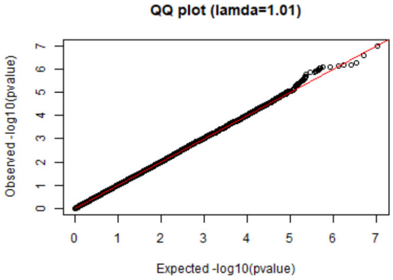
QQ plot (lamda=1.01)
Observed -log10(pvalue)
Expected -log10(pvalue)

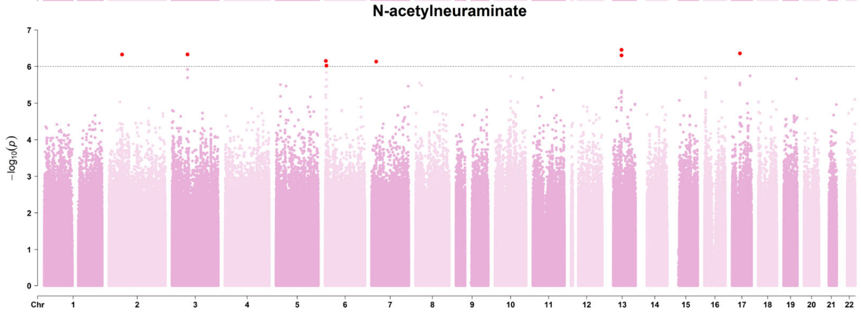
N-acetylneuraminate
$-\log_{10}(p)$
Chr

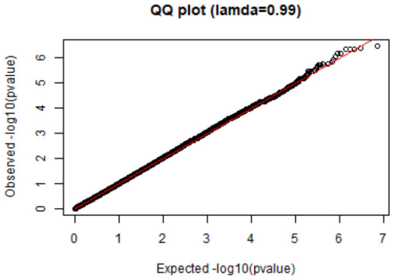
QQ plot (lamda=0.99)
Observed -log10(pvalue)
Expected -log10(pvalue)

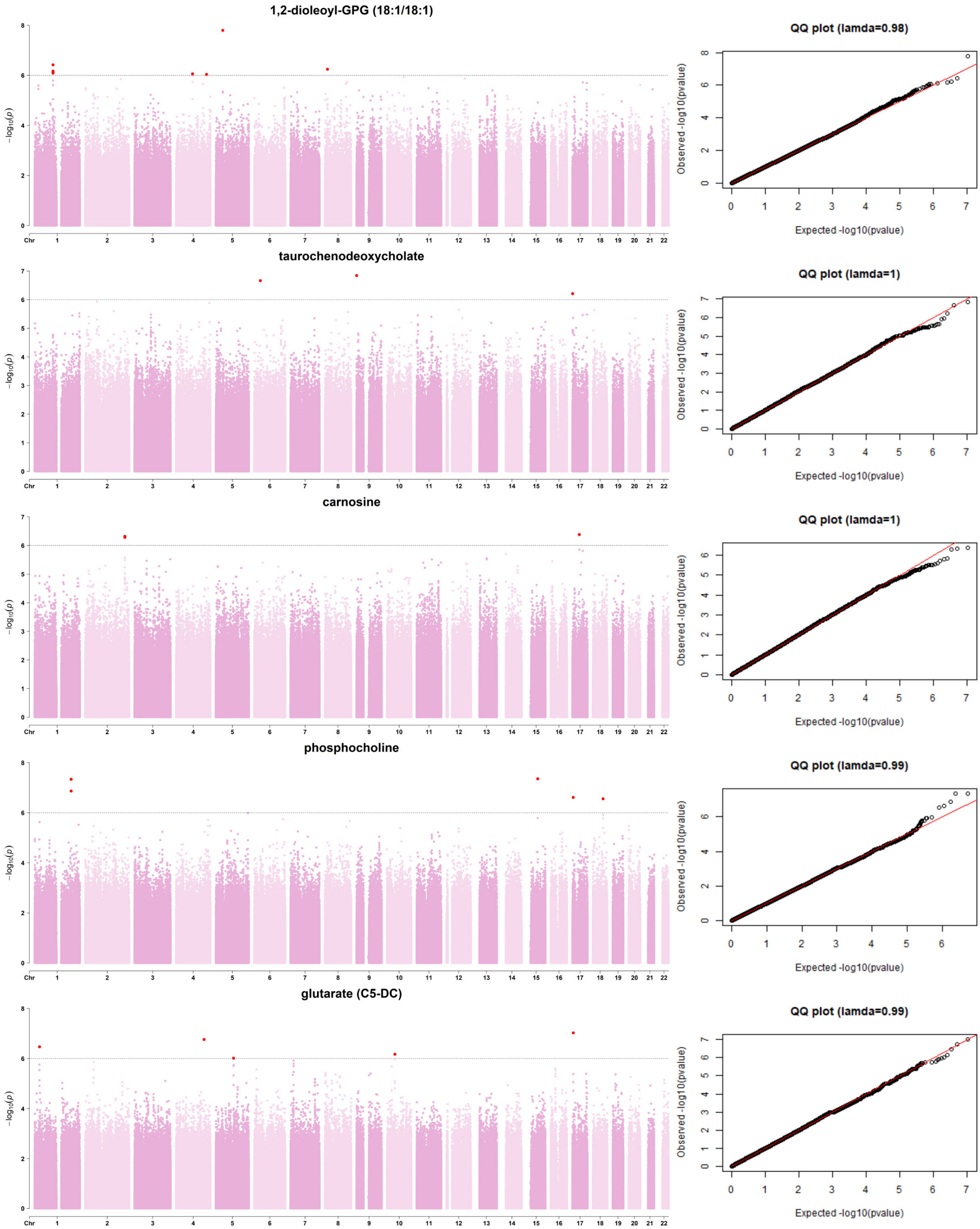
1,2-dioleoyl-GPG (18:1/18:1)
QQ plot (lamda=0.98)
taurochenodeoxycholate
QQ plot (lamda=1)
carnosine
QQ plot (lamda=1)
phosphocholine
QQ plot (lamda=0.99)
glutarate (C5-DC)
QQ plot (lamda=0.99)
$-\log_{10}(p)$
Chr 1 2 3 4 5 6 7 8 9 10 11 12 13 14 15 16 17 18 19 20 21 22
Observed -log10(pvalue)
Expected -log10(pvalue)

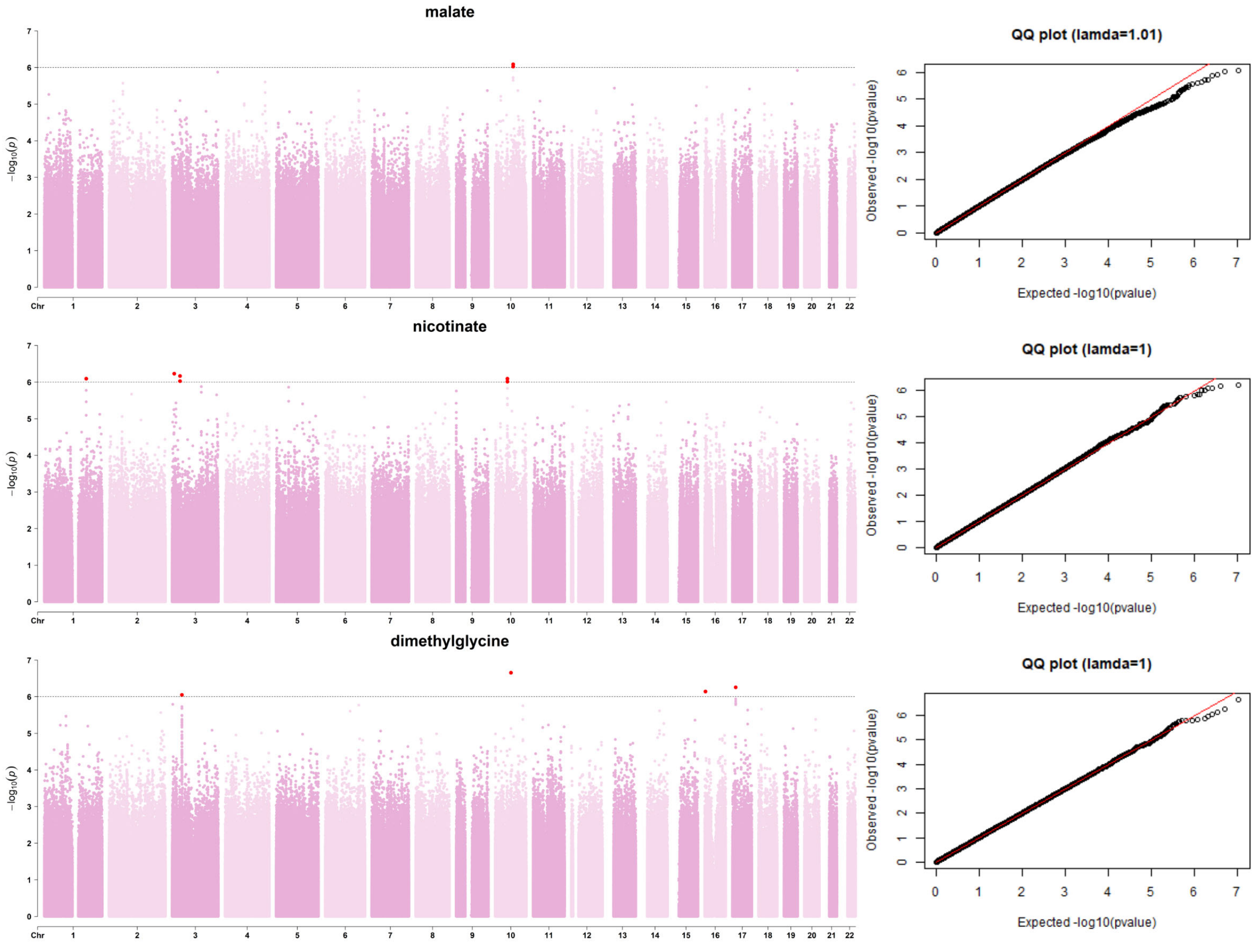
malate
QQ plot (lamda=1.01)
nicotinate
QQ plot (lamda=1)
dimethylglycine
QQ plot (lamda=1)
Observed -log10(pvalue)
Expected -log10(pvalue)
Chr

C. Hispanic sample

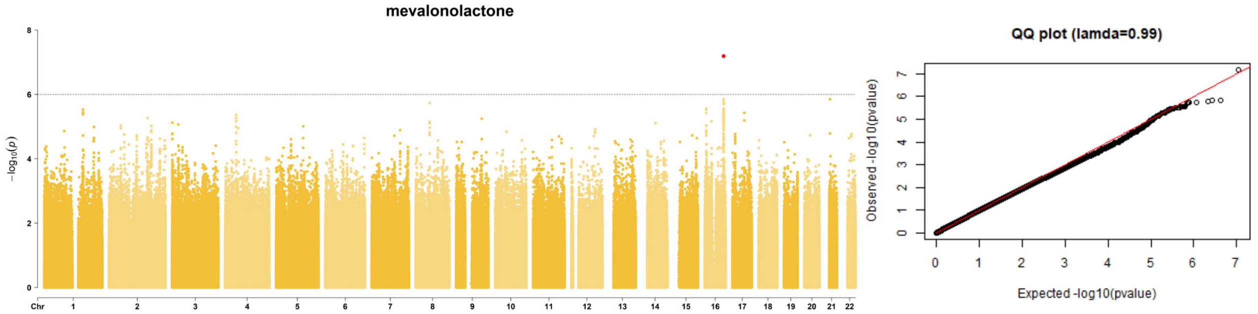

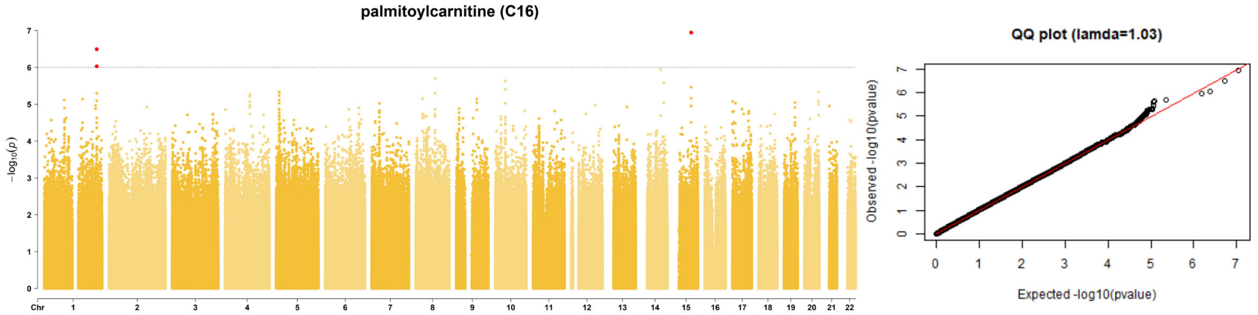

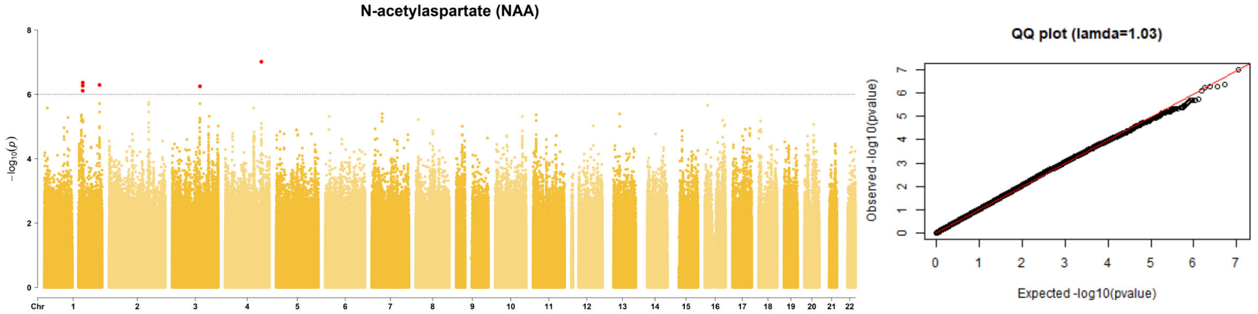

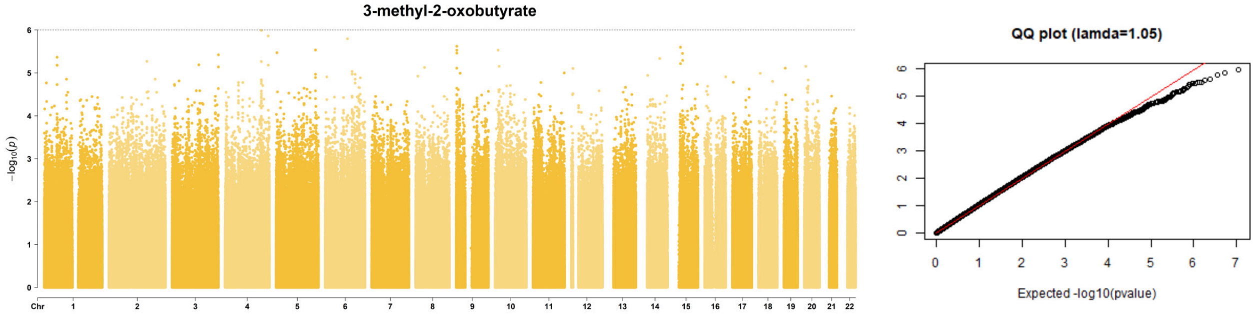

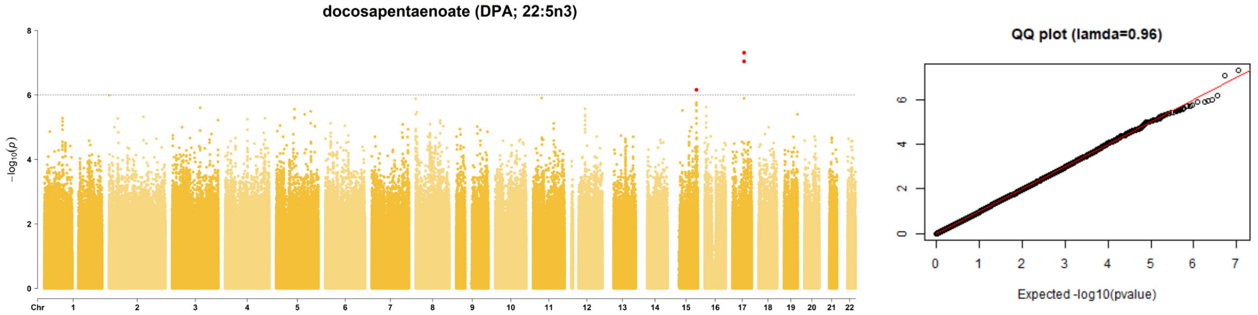

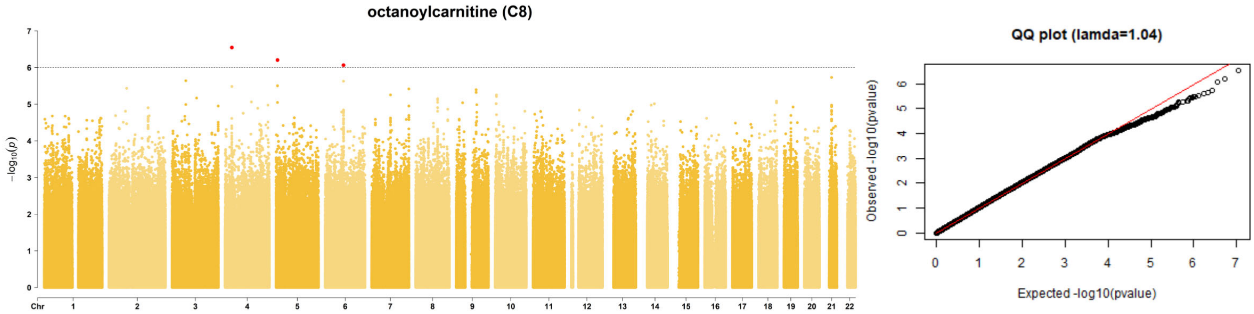

octanoylcarnitine (C8)
QQ plot (lamda=1.04)
Observed -log10(pvalue)
Expected -log10(pvalue)

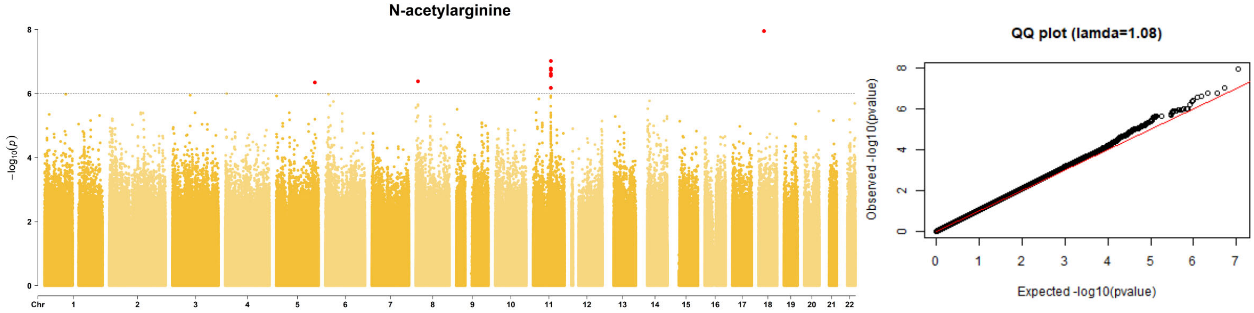

N-acetylarginine
QQ plot (lamda=1.08)
Observed -log10(pvalue)
Expected -log10(pvalue)

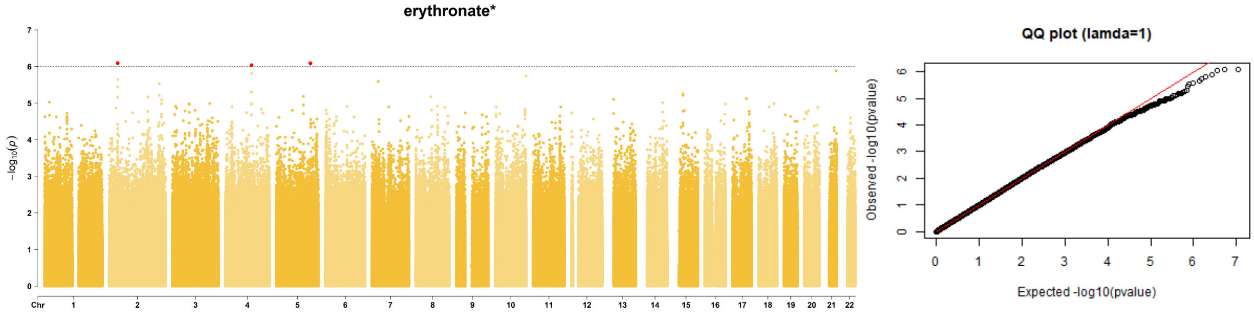

erythronate*
QQ plot (lamda=1)
Observed -log10(pvalue)
Expected -log10(pvalue)

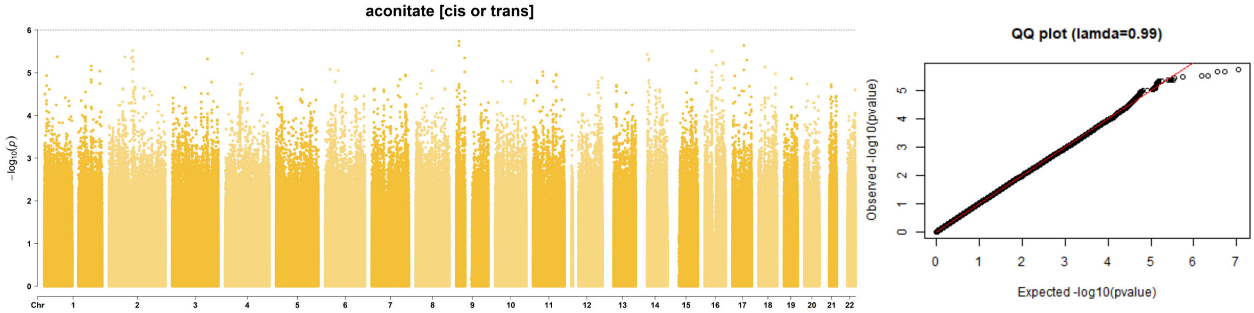

aconitate [cis or trans]
QQ plot (lamda=0.99)
Observed -log10(pvalue)
Expected -log10(pvalue)

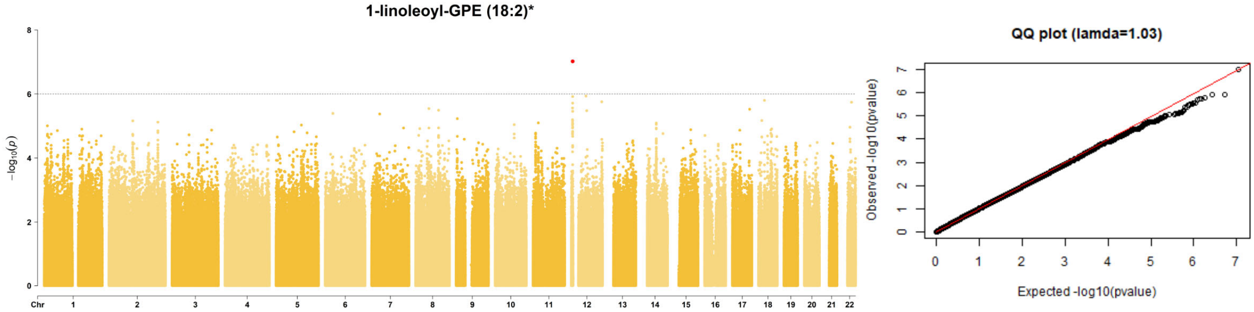

1-linoleoyl-GPE (18:2)*
QQ plot (lamda=1.03)
Observed -log10(pvalue)
Expected -log10(pvalue)

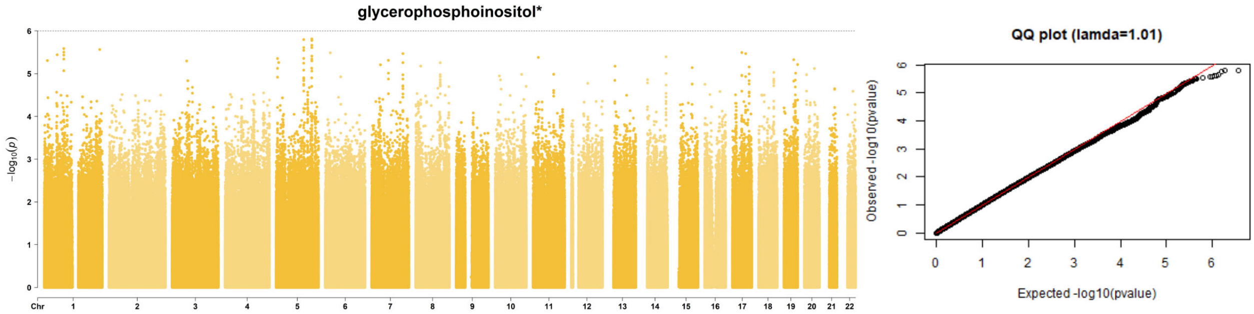
glycerophosphoinositol*
QQ plot (lamda=1.01)
Observed -log10(pvalue)
Expected -log10(pvalue)

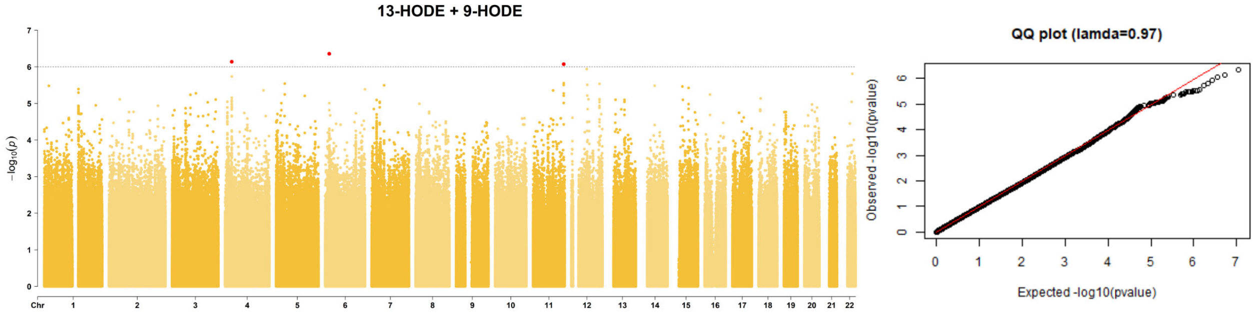
13-HODE + 9-HODE
QQ plot (lamda=0.97)
Observed -log10(pvalue)
Expected -log10(pvalue)

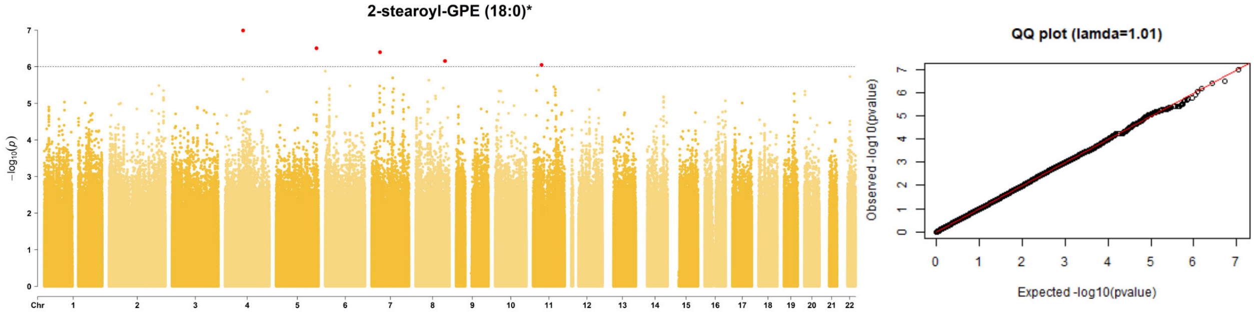
2-stearoyl-GPE (18:0)*
QQ plot (lamda=1.01)
Observed -log10(pvalue)
Expected -log10(pvalue)

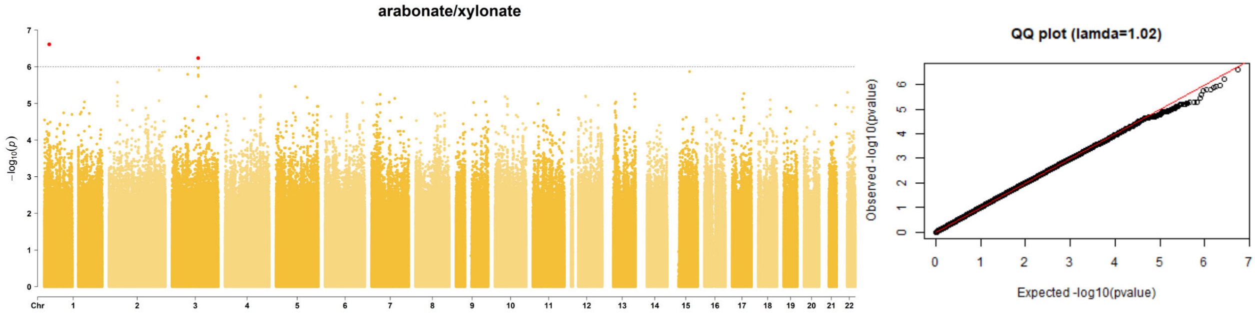
arabonate/xylonate
QQ plot (lamda=1.02)
Observed -log10(pvalue)
Expected -log10(pvalue)

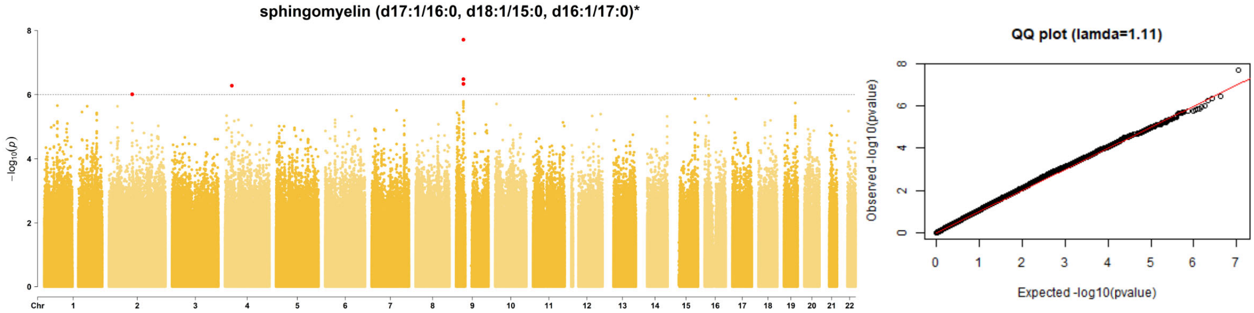
sphingomyelin (d17:1/16:0, d18:1/15:0, d16:1/17:0)*
QQ plot (lamda=1.11)
Observed -log10(pvalue)
Expected -log10(pvalue)

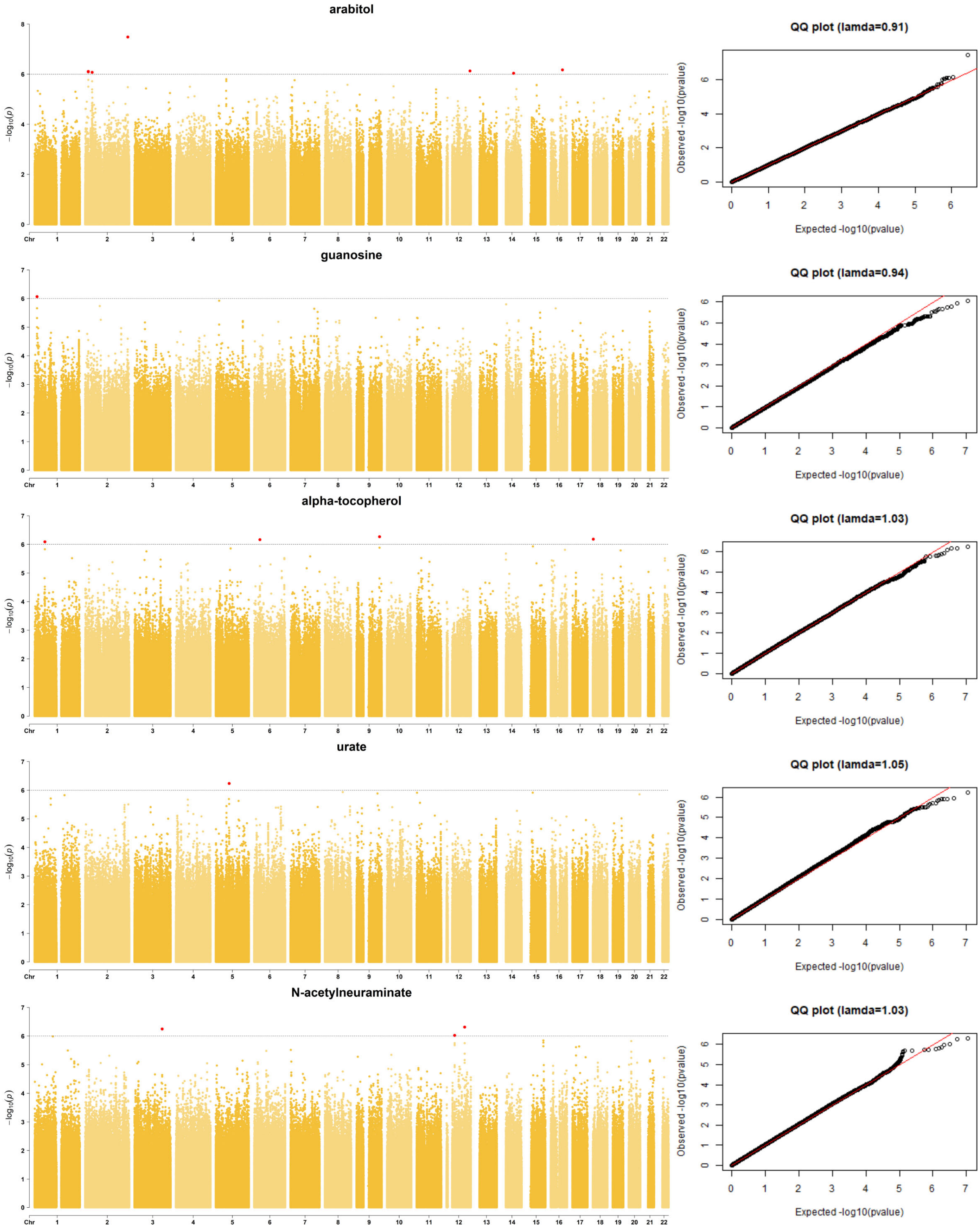
arabitol
QQ plot (lamda=0.91)
guanosine
QQ plot (lamda=0.94)
alpha-tocopherol
QQ plot (lamda=1.03)
urate
QQ plot (lamda=1.05)
N-acetylneuraminate
QQ plot (lamda=1.03)
$-\log_{10}(p)$
Chr
Observed -log10(pvalue)
Expected -log10(pvalue)

1,2-dioleoyl-GPG (18:1/18:1)

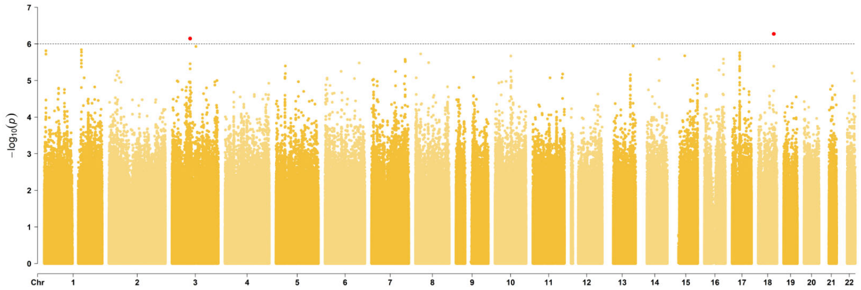

QQ plot (lamda=0.98)

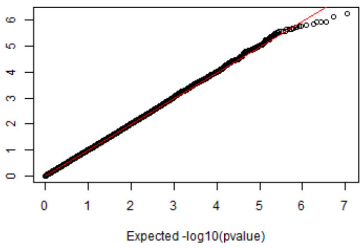

taurochenodeoxycholate

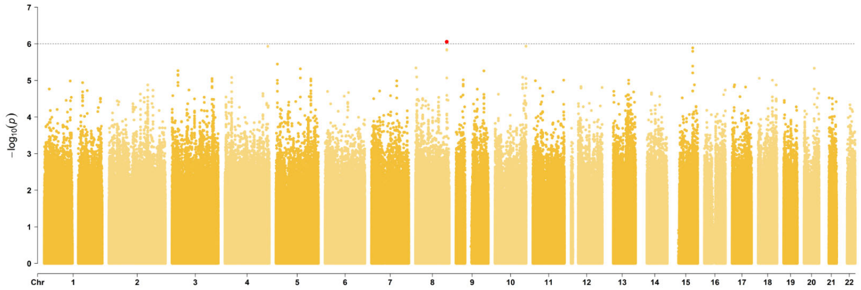

QQ plot (lamda=0.99)

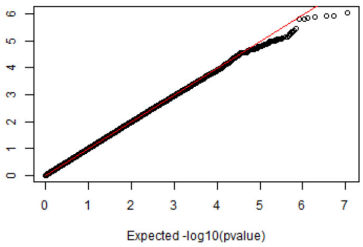

carnosine

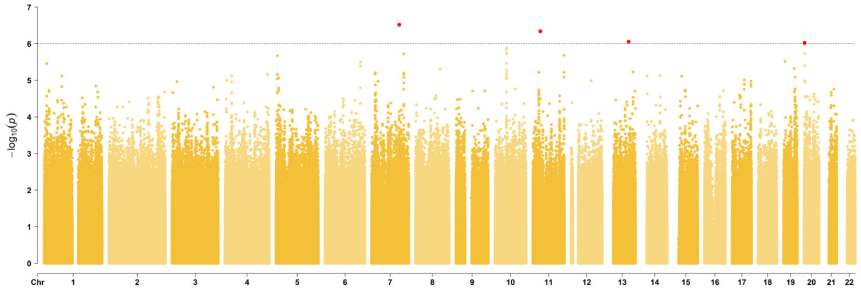

QQ plot (lamda=0.97)

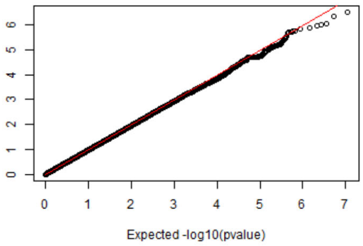

phosphocholine

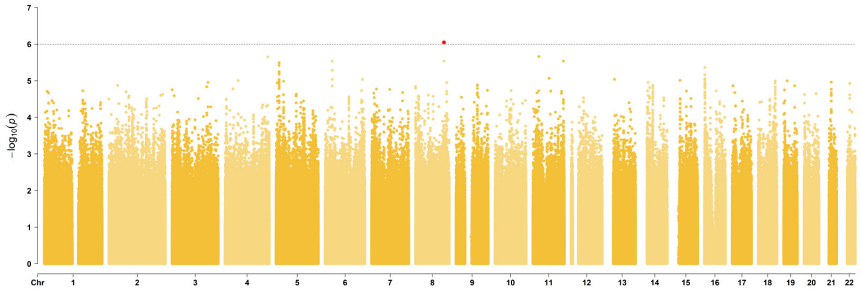

QQ plot (lamda=1.03)

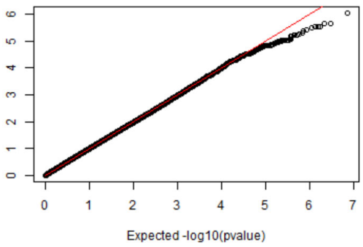

glutarate (C5-DC)

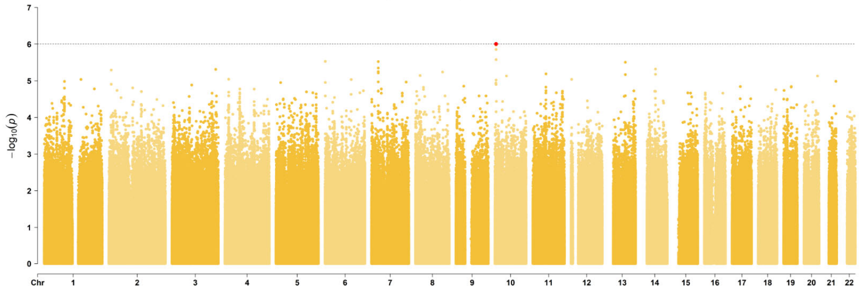

QQ plot (lamda=0.99)

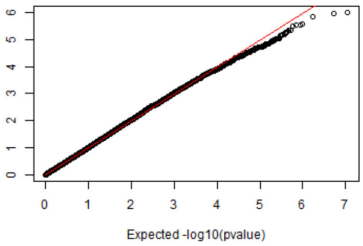

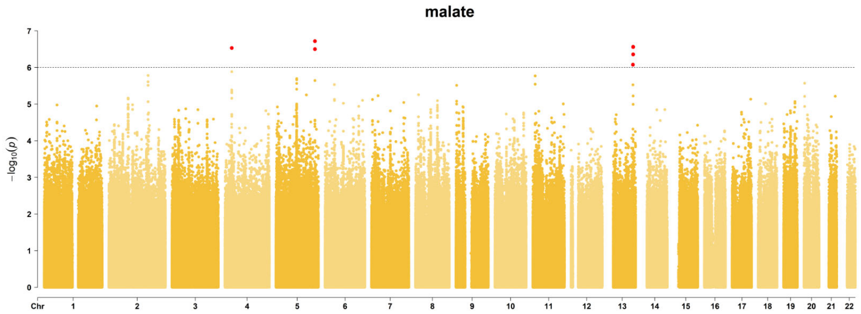
malate
−log10(p)
Chr 1 2 3 4 5 6 7 8 9 10 11 12 13 14 15 16 17 18 19 20 21 22
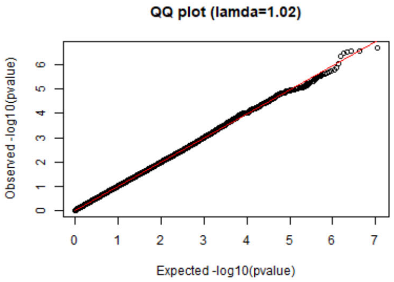
QQ plot (lamda=1.02)
Observed -log10(pvalue)
Expected -log10(pvalue)
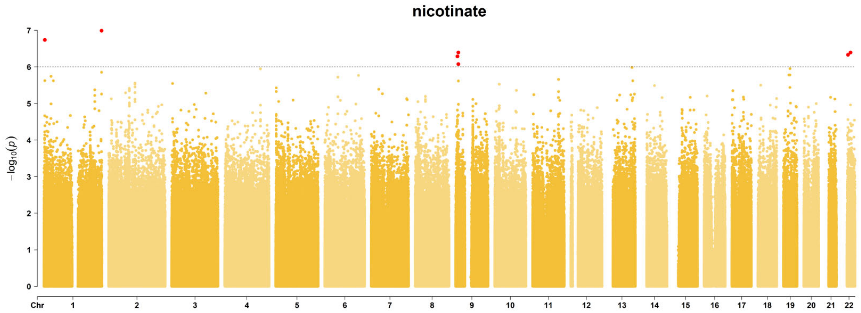
nicotinate
−log10(p)
Chr 1 2 3 4 5 6 7 8 9 10 11 12 13 14 15 16 17 18 19 20 21 22
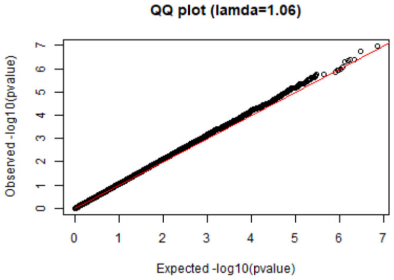
QQ plot (lamda=1.06)
Observed -log10(pvalue)
Expected -log10(pvalue)
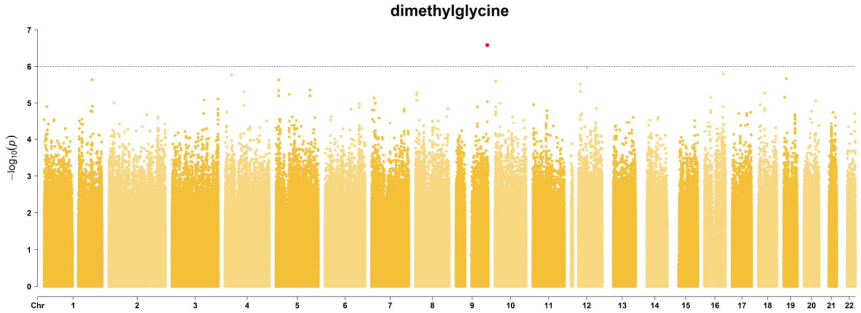
dimethylglycine
−log10(p)
Chr 1 2 3 4 5 6 7 8 9 10 11 12 13 14 15 16 17 18 19 20 21 22
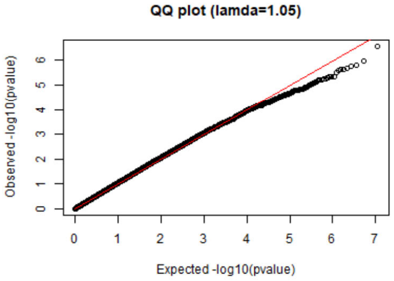
QQ plot (lamda=1.05)
Observed -log10(pvalue)
Expected -log10(pvalue)

Figure S2. Pathway analysis of genes within the clumped regions in the mtQTL analyses of each racial/ethnic sample
To examine the biological significance of the genetic loci identified in the mtQTL analyses, the over-representation pathway analyses were conducted using all genes within the clumped regions of each racial/ethnic sample. Gene sets with FDR of <0.05 were colored in a darker shade.
* The number of observed genes divided by the number of expected genes from the Reactome pathway category. The bar chart sorts result by enrichment ratio.

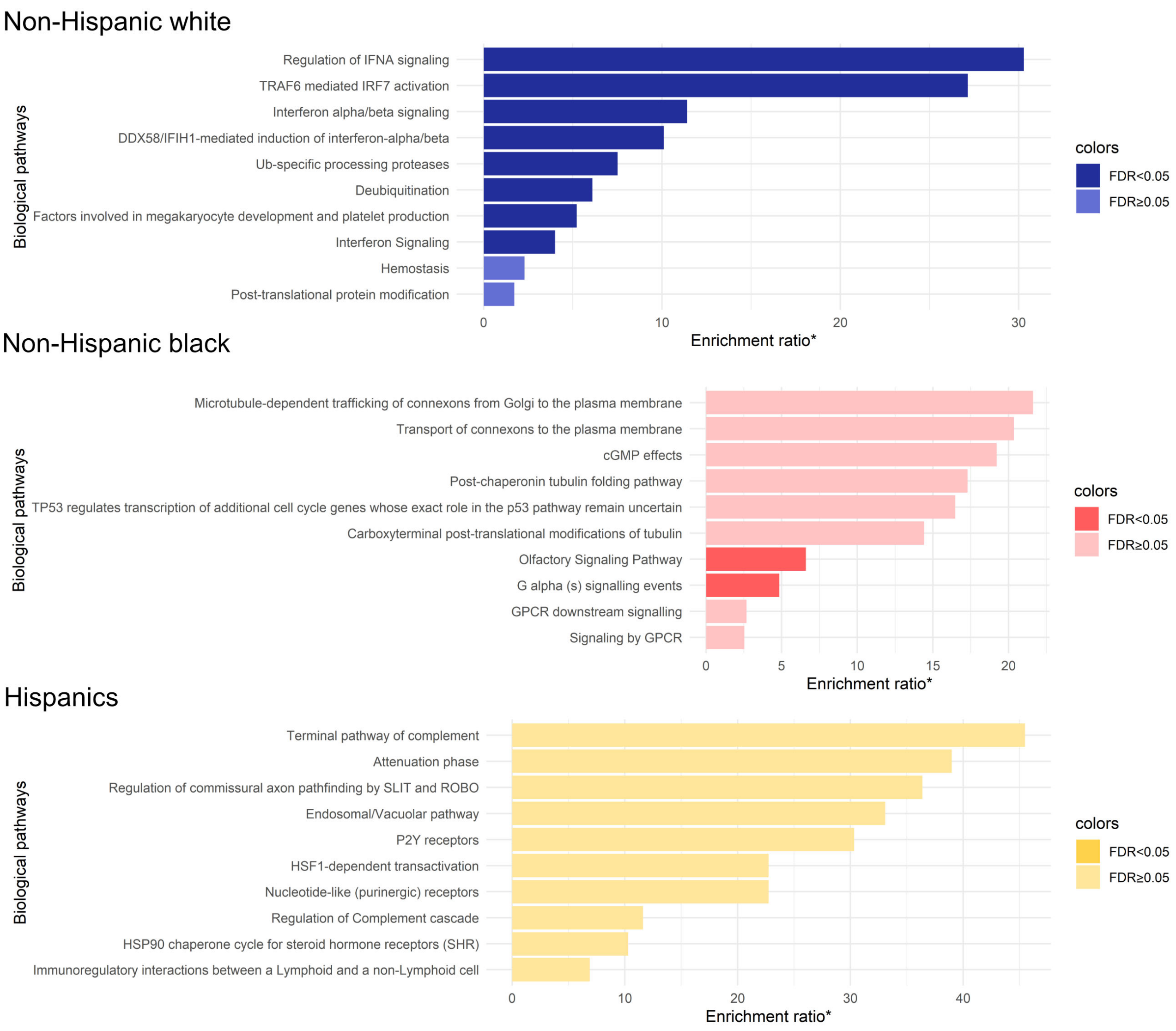

Figure S3. The PP.H4 values plot for the 281 genetic loci associated with the candidate metabolites in the mtQTL analyses

To test for causal variants that are in common between the candidate metabolites and asthma, colocalization analyses were performed for the 281 loci identified in the mtQTL analysis with a clumping procedure, and the PP.H4 values were calculated for each locus. Each dot represents a locus, the x-axis (PPH4_white) represents a PP.H4 value between the summary statistics of the mtQTL analysis in MARC-35 and the UK Biobank (white sample with childhood asthma), and the y-axis (PPH4_black) represents a PP.H4 value between the summary statistics of the mtQTL analysis and the CAAPA (African-admixed sample with asthma). The red dot with the "Overlap" label indicates that the genomic region of the locus overlapped with the other locus identified in another racial/ethnic sample.

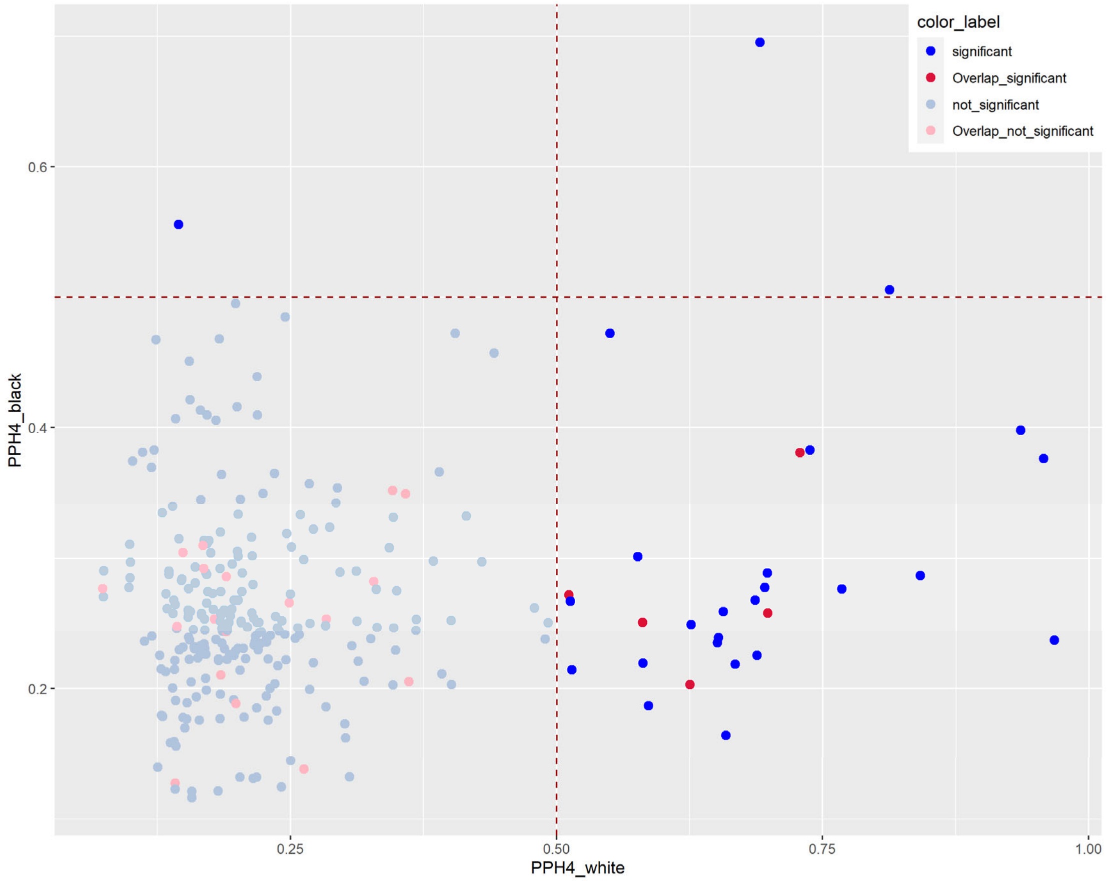

Figure S4. Comparison of the association peaks for the 30 loci with colocalization evidence between the mtQTL analyses and the UK Biobank statistics

To compare the association peaks for the 30 loci identified in the colocalization analysis between the mtQTL analysis of MARC-35 and the UK Biobank, the same genomic regions were visualized with the summary statistics of these two datasets.

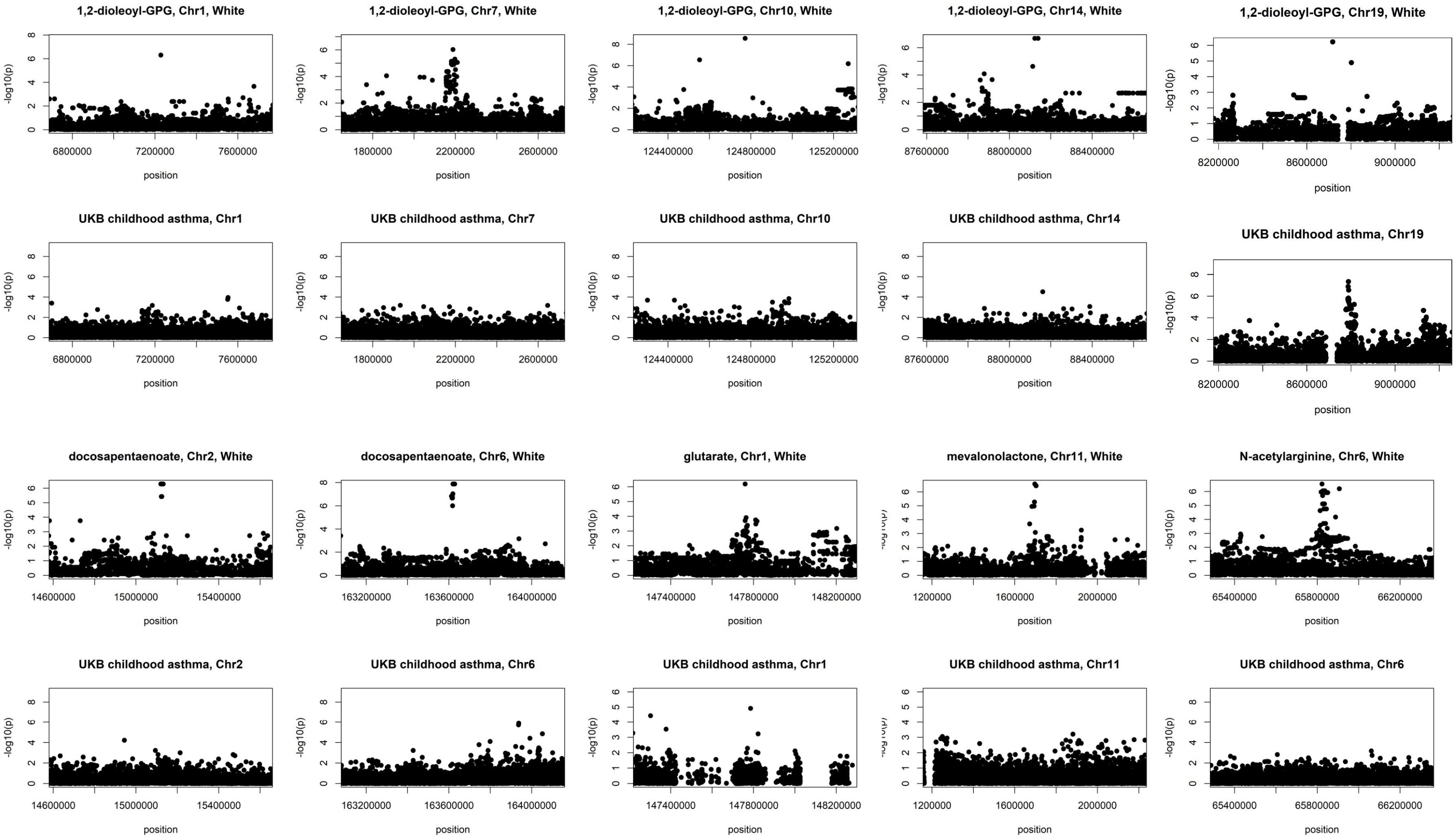

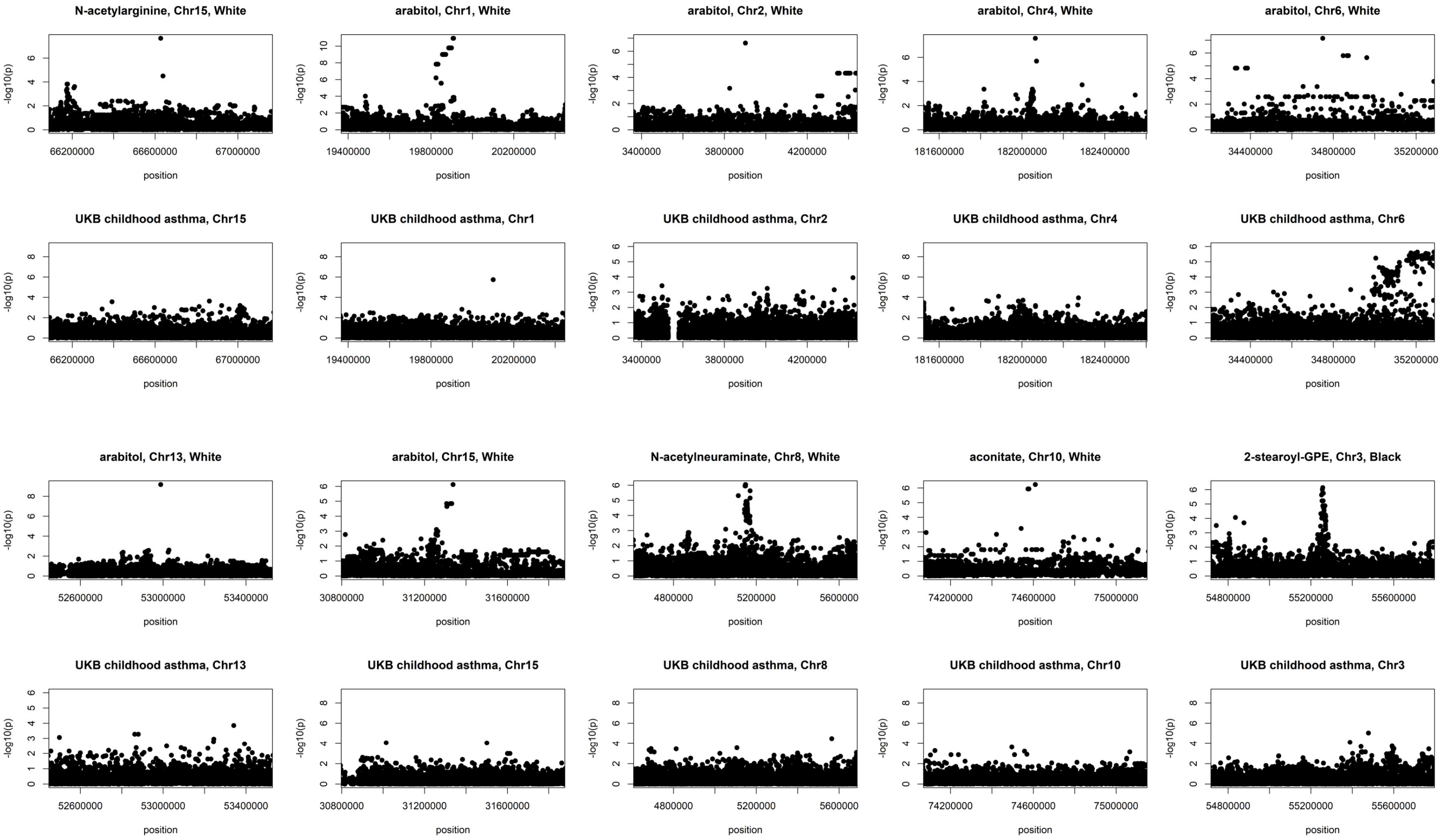
N-acetylarginine, Chr15, White
arabitol, Chr1, White
arabitol, Chr2, White
arabitol, Chr4, White
arabitol, Chr6, White
UKB childhood asthma, Chr15
UKB childhood asthma, Chr1
UKB childhood asthma, Chr2
UKB childhood asthma, Chr4
UKB childhood asthma, Chr6
arabitol, Chr13, White
arabitol, Chr15, White
N-acetylneuraminate, Chr8, White
aconitate, Chr10, White
2-stearoyl-GPE, Chr3, Black
UKB childhood asthma, Chr13
UKB childhood asthma, Chr15
UKB childhood asthma, Chr8
UKB childhood asthma, Chr10
UKB childhood asthma, Chr3
-log10(p)
position
66200000 66600000 67000000
19400000 19800000 20200000
3400000 3800000 4200000
181600000 182000000 182400000
34400000 34800000 35200000
52600000 53000000 53400000
30800000 31200000 31600000
4800000 5200000 5600000
74200000 74600000 75000000
54800000 55200000 55600000

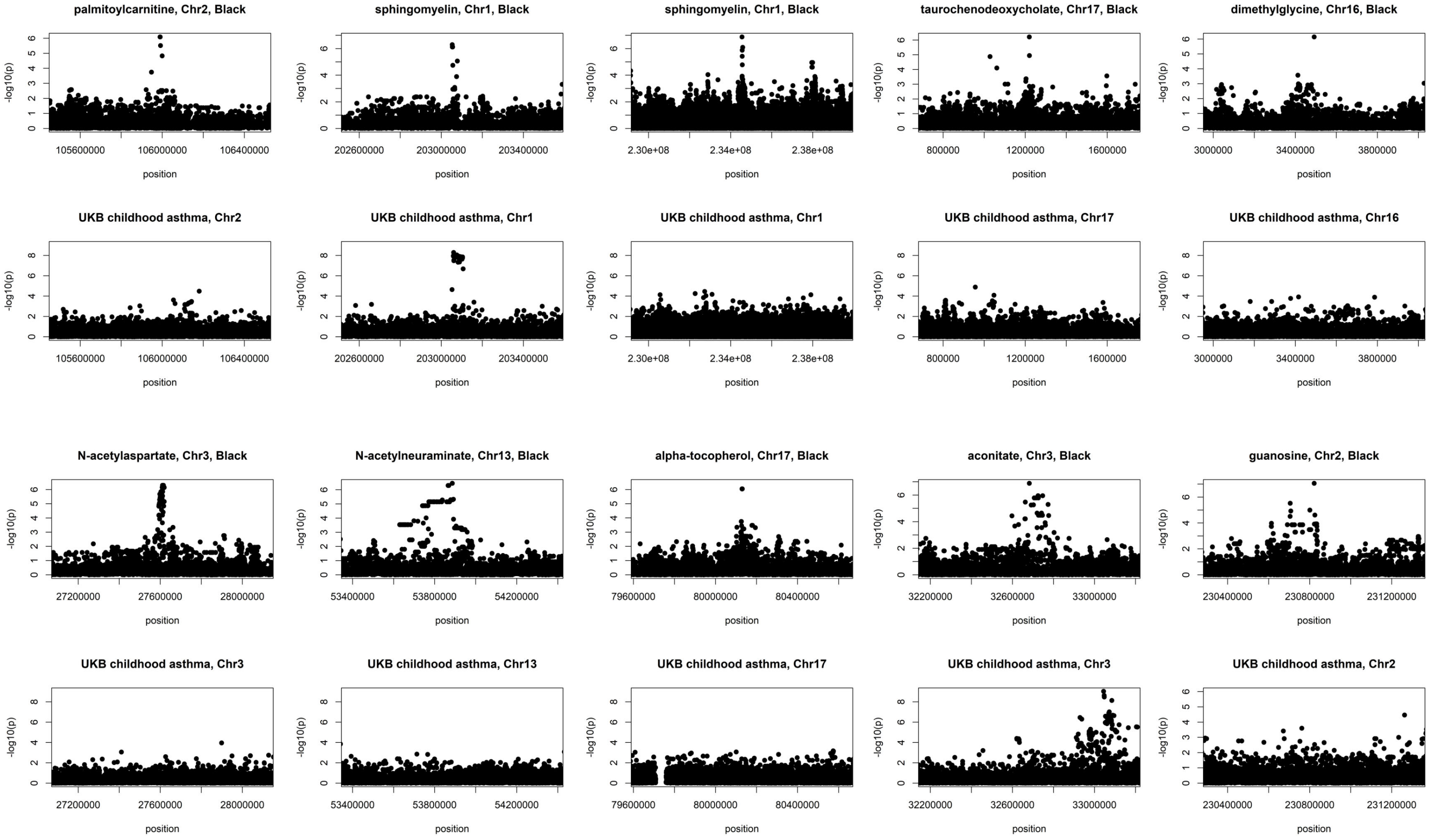
palmitoylcarnitine, Chr2, Black
sphingomyelin, Chr1, Black
sphingomyelin, Chr1, Black
taurochenodeoxycholate, Chr17, Black
dimethylglycine, Chr16, Black
UKB childhood asthma, Chr2
UKB childhood asthma, Chr1
UKB childhood asthma, Chr1
UKB childhood asthma, Chr17
UKB childhood asthma, Chr16
N-acetylaspartate, Chr3, Black
N-acetylneuraminate, Chr13, Black
alpha-tocopherol, Chr17, Black
aconitate, Chr3, Black
guanosine, Chr2, Black
UKB childhood asthma, Chr3
UKB childhood asthma, Chr13
UKB childhood asthma, Chr17
UKB childhood asthma, Chr3
UKB childhood asthma, Chr2
-log10(p)
position
105600000
106000000
106400000
202600000
203000000
203400000
2.30e+08
2.34e+08
2.38e+08
800000
1200000
1600000
3000000
3400000
3800000
27200000
27600000
28000000
53400000
53800000
54200000
79600000
80000000
80400000
32200000
32600000
33000000
230400000
230800000
231200000
